# Supplementary material for: A platform trial of neoadjuvant and adjuvant antitumor vaccination alone or in combination with PD-1 antagonist and CD137 agonist antibodies in patients with resectable pancreatic adenocarcinoma
Source: Nat Commun. 2023 Jun 20;14:3650. doi: 10.1038/s41467-023-39196-9 (PMC10281953; doi:10.1038/s41467-023-39196-9)
Supplement: Supplementary file 1 — Supplementary Information [file 41467_2023_39196_MOESM1_ESM.pdf]

<u>Supplementary Information</u>

Contents

Supplementary Figures

Supplementary Figure 1: Schema of Study Enrollment.

Supplementary Figure 2: (a) Disease-Free and (b) Overall Survival KM Curves Stratified by Median Density of CD8+CD137+ Tumor-Infiltrating T cells following one GVAX-based Treatment.

Supplementary Figure 3: Non-parametric Spearman correlation between CD3+CD8+CD137+ cell density and CD3+CD8+GZMB+ cell density in resected tumor specimens.

Supplementary Figure 4: (a) Disease-Free and (b) Overall Survival KM Curves Stratified by Median Density of CD8+GZMB+ Tumor-Infiltrating T cells following one GVAX-based Treatment.

Supplementary Figure 5: (a) Disease-Free and (b) Overall Survival KM Curves Stratified by Median Density of CD8+CD137+GZMB+ Tumor-Infiltrating T cells following one GVAX-based Treatment.

Supplementary Figure 6: (a) Disease-Free and (b) Overall Survival Kaplan Meier Curves Stratified SOC Adjuvant Chemotherapy Administered.

Supplementary Figure 7: Swimmer plot of disease-free and overall survival time of individual study participants.

Supplementary Figure 8: Disease-Free Survival Kaplan-Meier Curve comparing J1568 Arm C and Matched Historical Control Cohort.

Supplementary Figure 9: Comparison of the Densities of Multiple Immune Cell Subtypes between Treatment Arms.

Supplementary Tables

Supplementary Table 1: Cox Proportional Hazard Model for Disease-Free and Overall Survival for Tumor Tissue Covariates.

Supplementary Table 2: Cox Proportional Hazard Model for Disease-Free Survival for Clinical Covariates.

Supplementary Table 3: Cox Proportional Hazard Model for Overall Survival for Clinical Covariates.

Supplementary Table 4: J1568 Arm C and Historical Control Patient Baseline Characteristics.

Supplementary Table 5: Disease-Free Survival Comparisons Between J1568 Arm C and Matched Historical Control Cohort.

Supplementary Table 6: Summary of disease-free survival (DFS) and overall survival (OS) in the J1568 in context of previous vaccine, immunotherapy, and historical benchmark adjuvant therapy trials in resected pancreatic adenocarcinoma.

Supplementary Table 7: The multiplex IHC panel.

Supplementary Note

Study Protocol

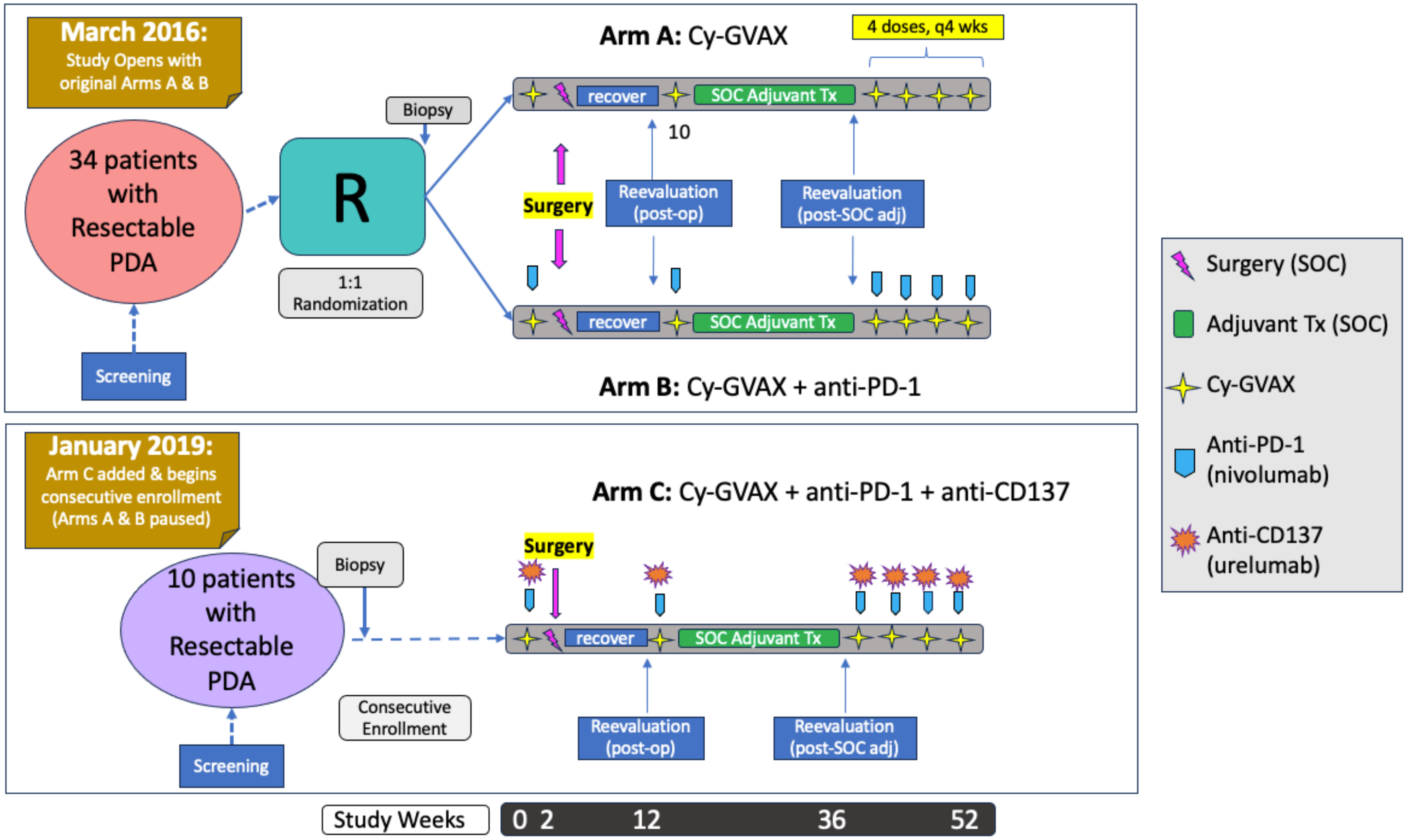

Supplementary Figure 1: Schema of Study Enrollment. Beginning in March 2016, eligible patients were enrolled and randomized 1:1 into the initial two treatment arms, Cy-GVAX (Arm A) or Cy-GVAX plus nivolumab (Arm B). Randomization was stratified by age of enrollment (≤65 and >65 years old). In October 2018, the study protocol was amended to include the following: 1) A 3rd treatment group (Arm C) was opened for patients to receive Cy-GVAX plus nivolumab and urelumab; 2) Nivolumab dose was changed from 3mg/kg to 480mg flat dose based on updated approved dosing guidelines; 3) An optional extended treatment phase was added following initial priming phase for pts who remained disease free. Due to the discontinuation of urelumab production, it was necessary to enrolling Arm C patients consecutively in order to meet this Arm C accrual goal. Following completion of Arm C accrual enrollment to Arm A and B resumed in a randomized fashion. Due to plans to add new treatment Arms for this patient population, Arms A & B were then closed and final analysis was conducted.

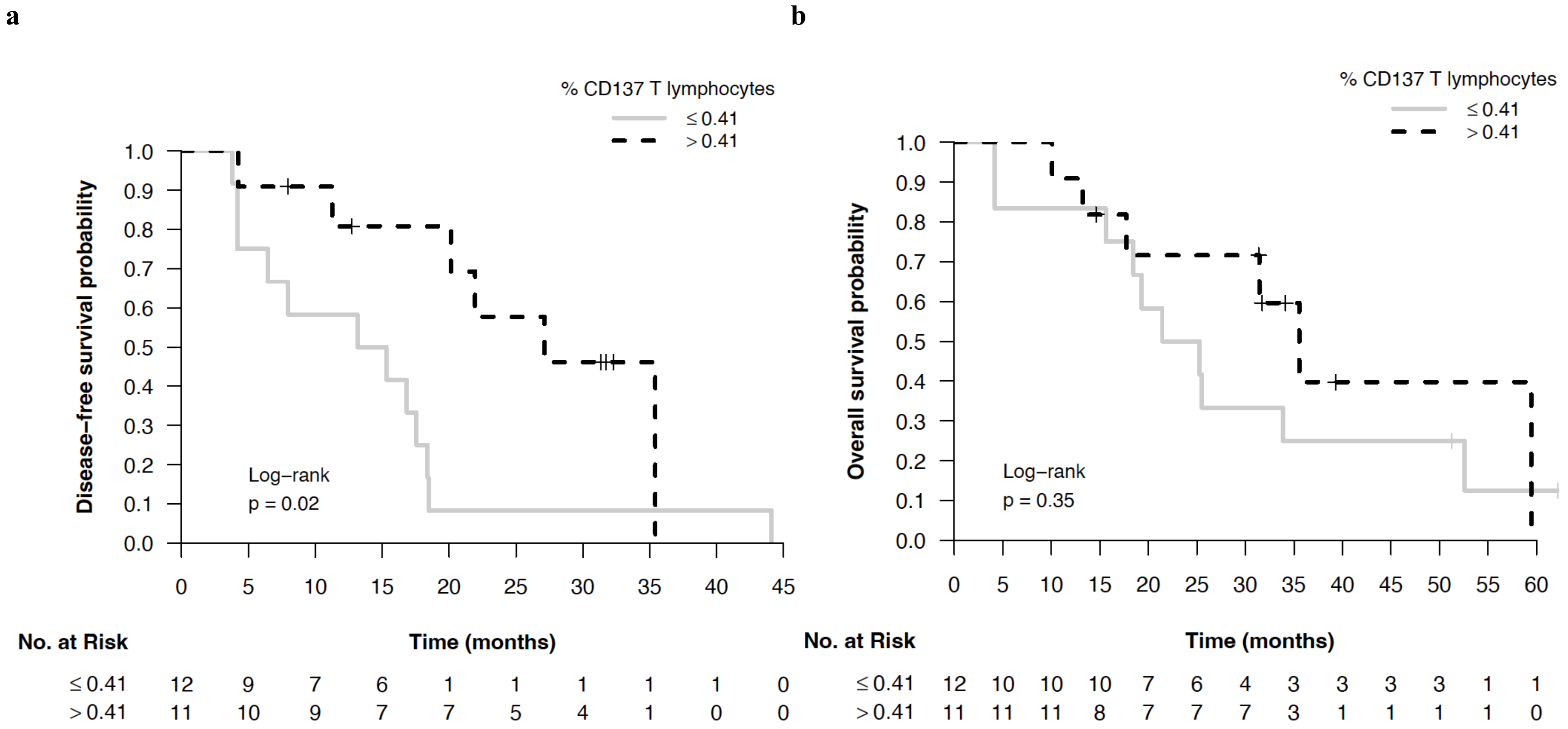

Supplementary Figure 2: (a) Disease-Free and (b)Overall Survival KM Curves Stratified by Median Percentage of CD3+CD8+CD137+ Tumor-Infiltrating T cells following one GVAX-based Treatment (All Arms, [n=23]). The multiplex immunohistochemistry (mIHC) workflow was able to be performed on surgical specimens from 23 study patient: n=7 (Arm A), n=8 (Arm B), n= 8 (Arm C). Reflects an averaged T cell subtype density within evaluated regions of interest (ROI) containing tertiary lymphoid aggregates (TLA) per specimen. This was chosen instead of absolute numbers to reflect the proportion of this cell type and to normalize comparisons between ROIs/TLAs within and across resected samples. Patients are grouped by median density across groups (above and below)

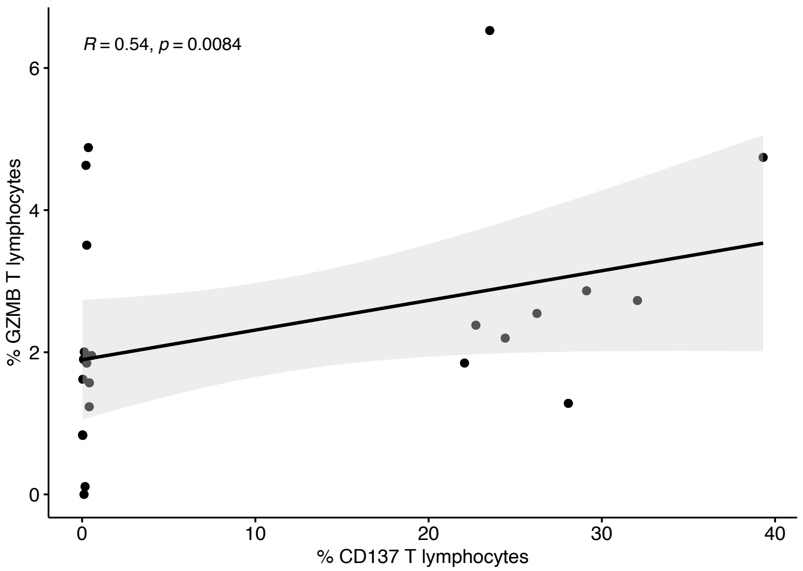

Supplementary Figure 3: Non-parametric, two-sided, Spearman correlation between CD3+CD8+CD137+ cell density and CD3+CD8+GZMB+ cell density in resected tumor specimens **(**N=28; n=10 [Arm A], n= 10 [Arm B], n= 8 [Arm C]**).**

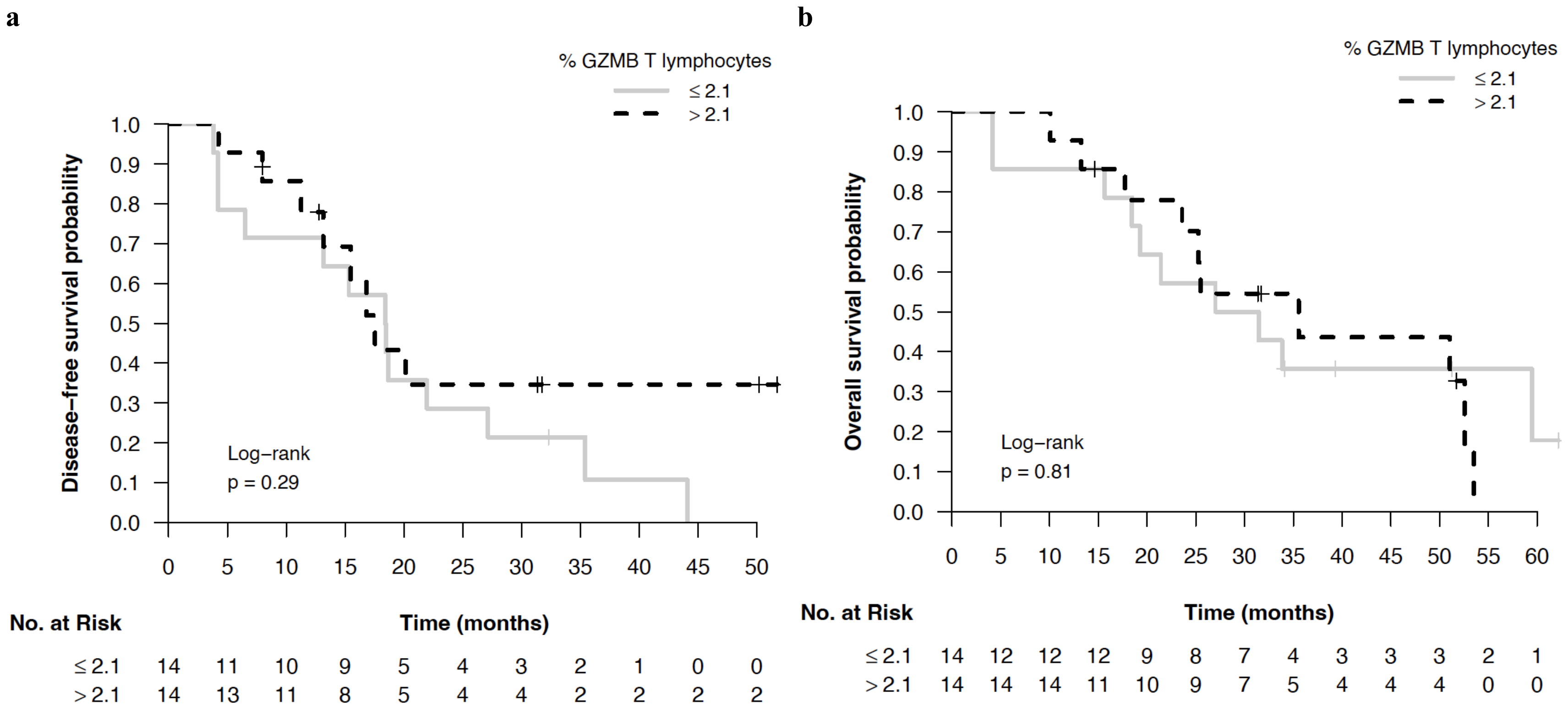

Supplementary Figure 4: (a) Disease-Free and (b)Overall Survival KM Curves Stratified by Median Density of CD3+CD8+GZMB+ Tumor-Infiltrating T cells following one GVAX-based Treatment (All Arms, [n=28]). The multiplex immunohistochemistry (mIHC) workflow was able to be performed on surgical specimens from 28 study patients: n= 10 (Arm A), n= 10 (Arm B), n= 8 (Arm C). Reflects an averaged T cell subtype density within evaluated regions of interest (ROI) containing tertiary lymphoid aggregates (TLA) per specimen. This was chosen instead of absolute numbers to reflect the proportion of this cell type and to normalize comparisons between ROIs/TLAs within and across resected samples. Patients are grouped by median density across groups (above and below).

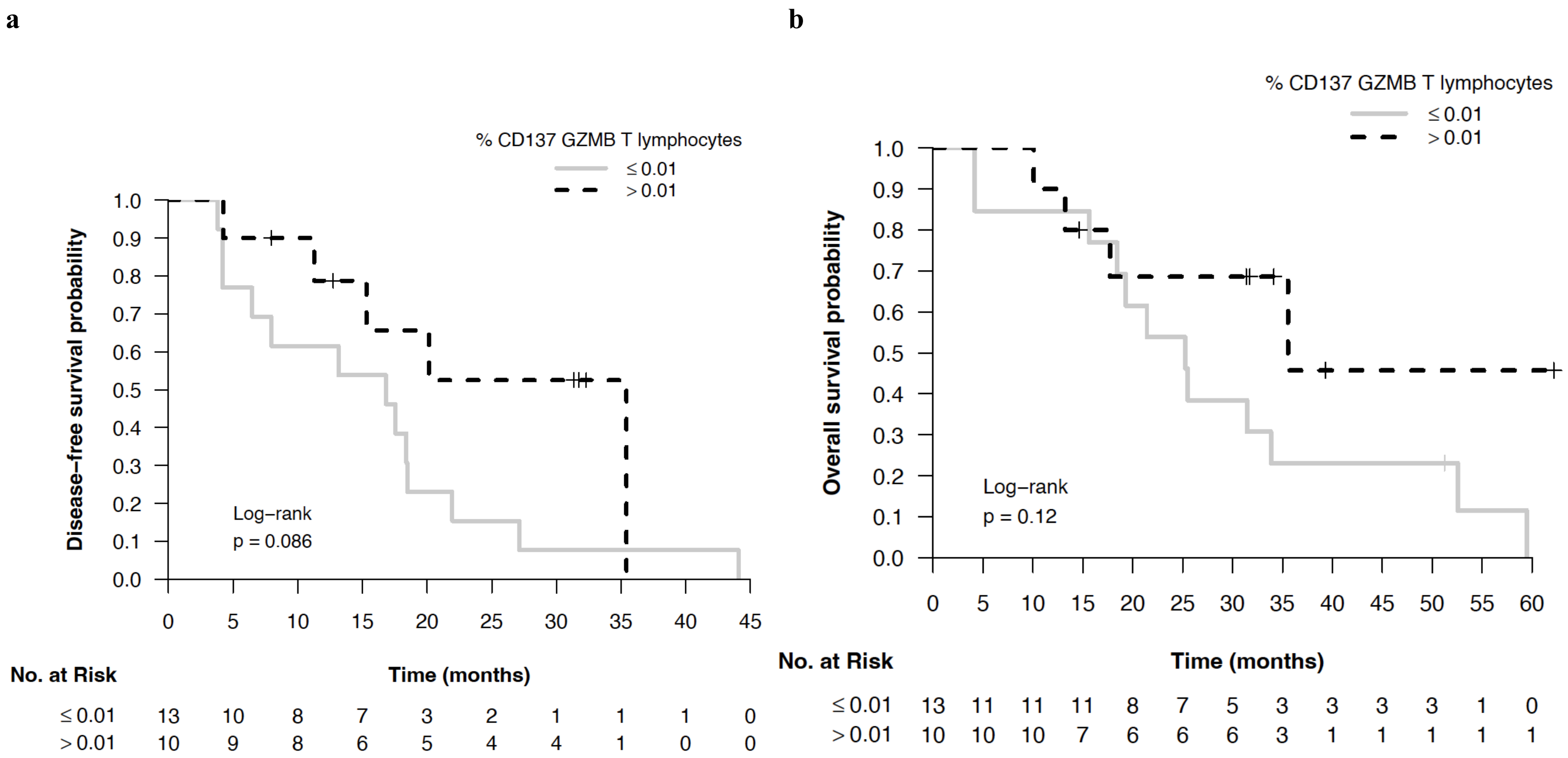

Supplementary Figure 5: (a) Disease-Free and (b)Overall Survival KM Curves Stratified by Median Density of CD3+CD8+CD137+GZMB+ Tumor-Infiltrating T cells following one GVAX-based Treatment (All Arms, [n=23]). The multiplex immunohistochemistry (mIHC) workflow was able to be performed on surgical specimens from 23 study patient: n=7 (Arm A), n=8 (Arm B), n= 8 (Arm C). Reflects an averaged T cell subtype density within evaluated regions of interest (ROI) containing tertiary lymphoid aggregates (TLA) per specimen. This was chosen instead of absolute numbers to reflect the proportion of this cell type and to normalize comparisons between ROIs/TLAs within and across resected samples. Patients are grouped by median density across groups (above and below).

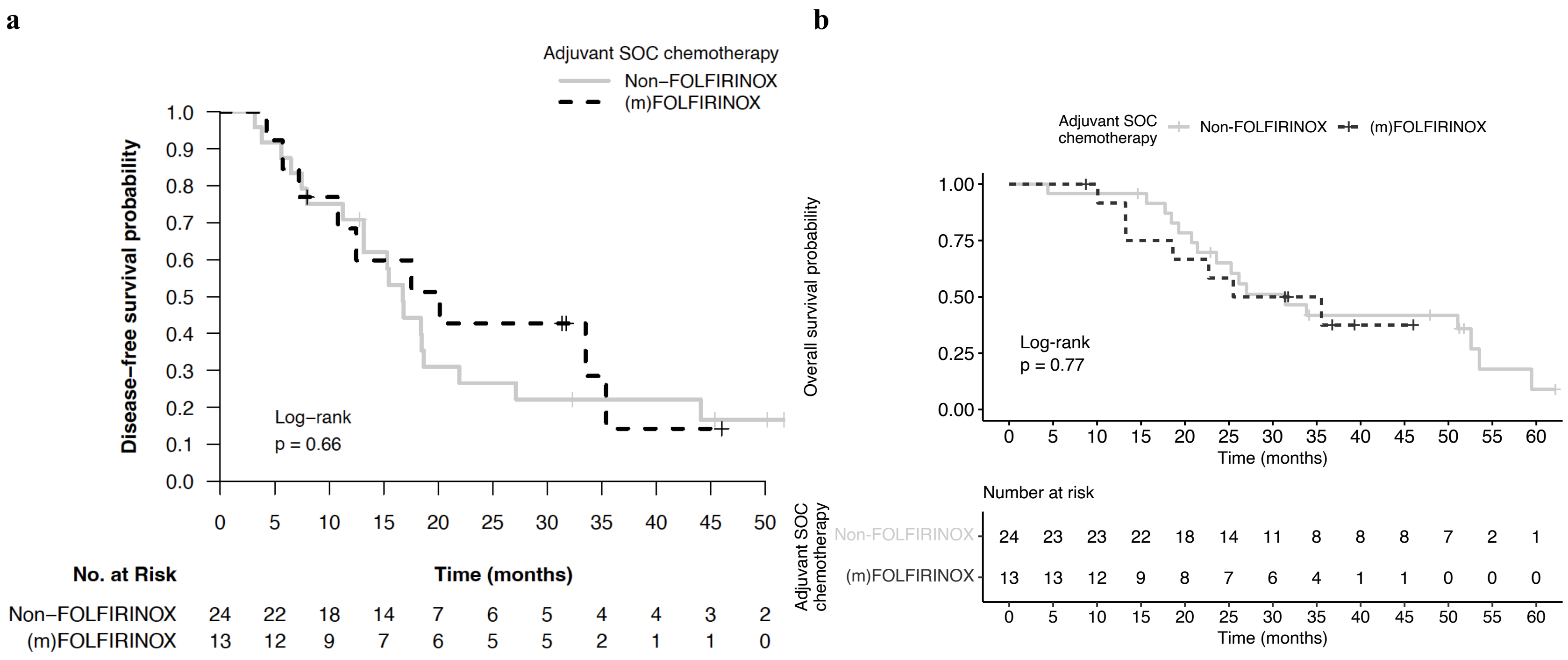

Supplementary Figure 6: (a) Disease-Free and (b) Overall Survival Kaplan Meier Curves Stratified SOC Adjuvant Chemotherapy Administered (Efficacy Cohort [n=40])**.** Non-FOLFIRINOX regimens included gemcitabine-capecitabine, gemcitabine-nabPaclitaxel, gemcitabine monotherapy.

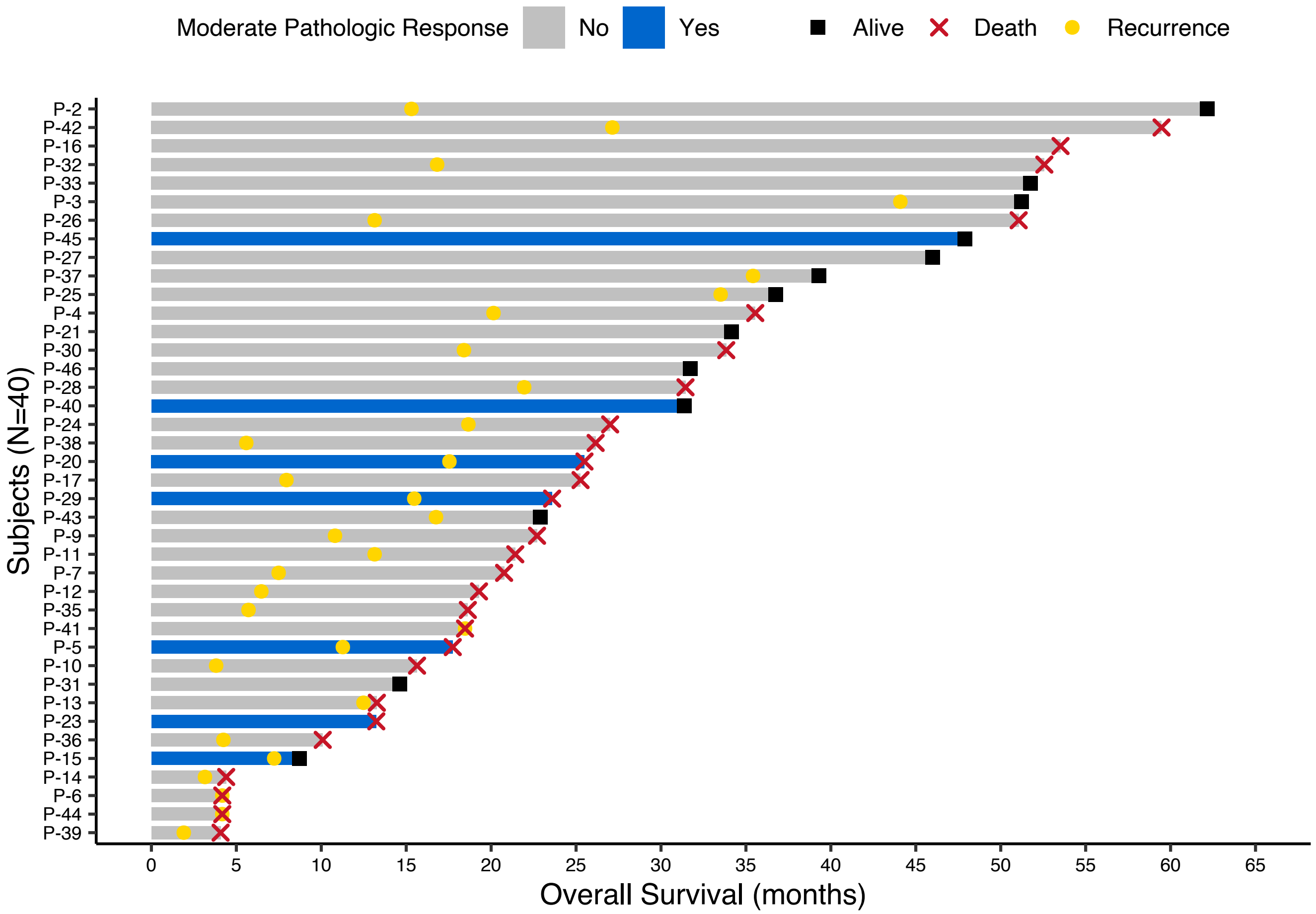

Supplementary Figure 7: Swimmer plot of disease-free and overall survival time of individual study participants. Moderate pathologic response is based on CAP grade 2 treatment response noted upon surgical resection following one neoadjuvant dose of study therapy (Efficacy Cohort [n=40]).

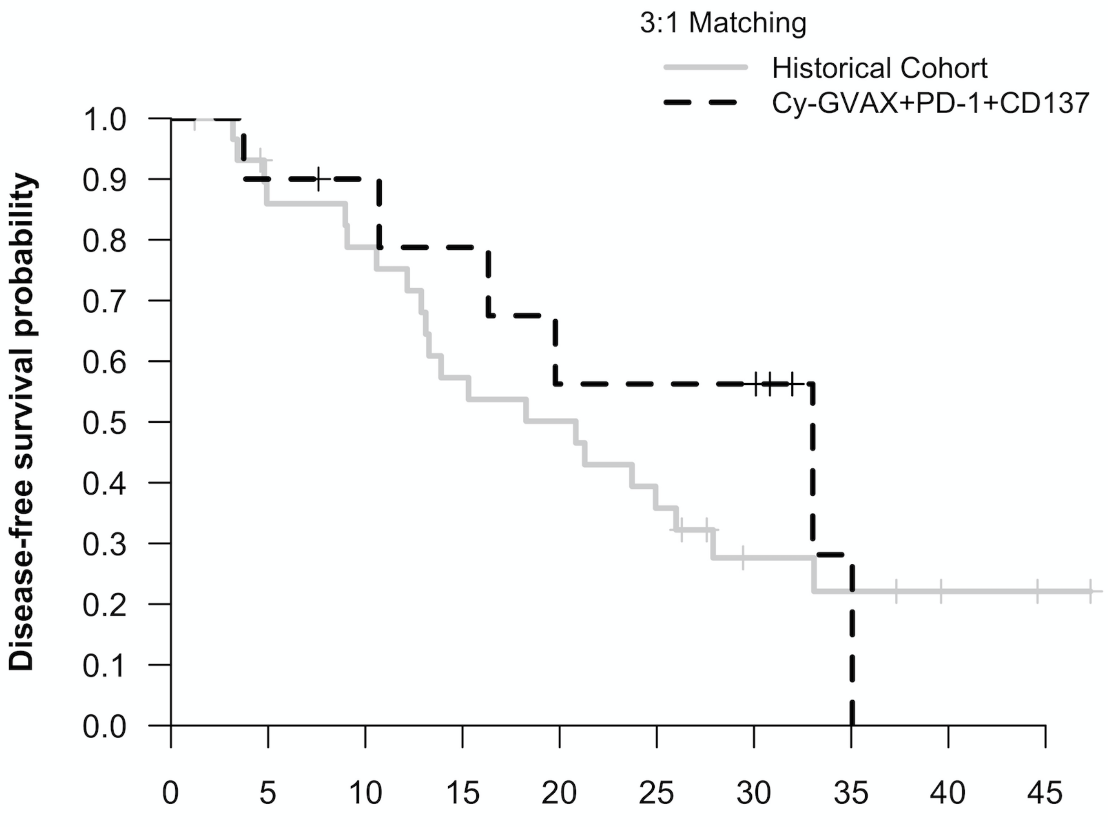

No. at Risk	Time (months)									
Historical Cohort	30	24	22	16	14	10	5	4	2	1
Cy-GVAX+PD-1+CD137	10	9	8	7	5	5	5	1	0	0

Supplementary Figure 8: Disease-Free Survival Kaplan-Meier Curve comparing J1568 Arm C and Matched Historical Control Cohort

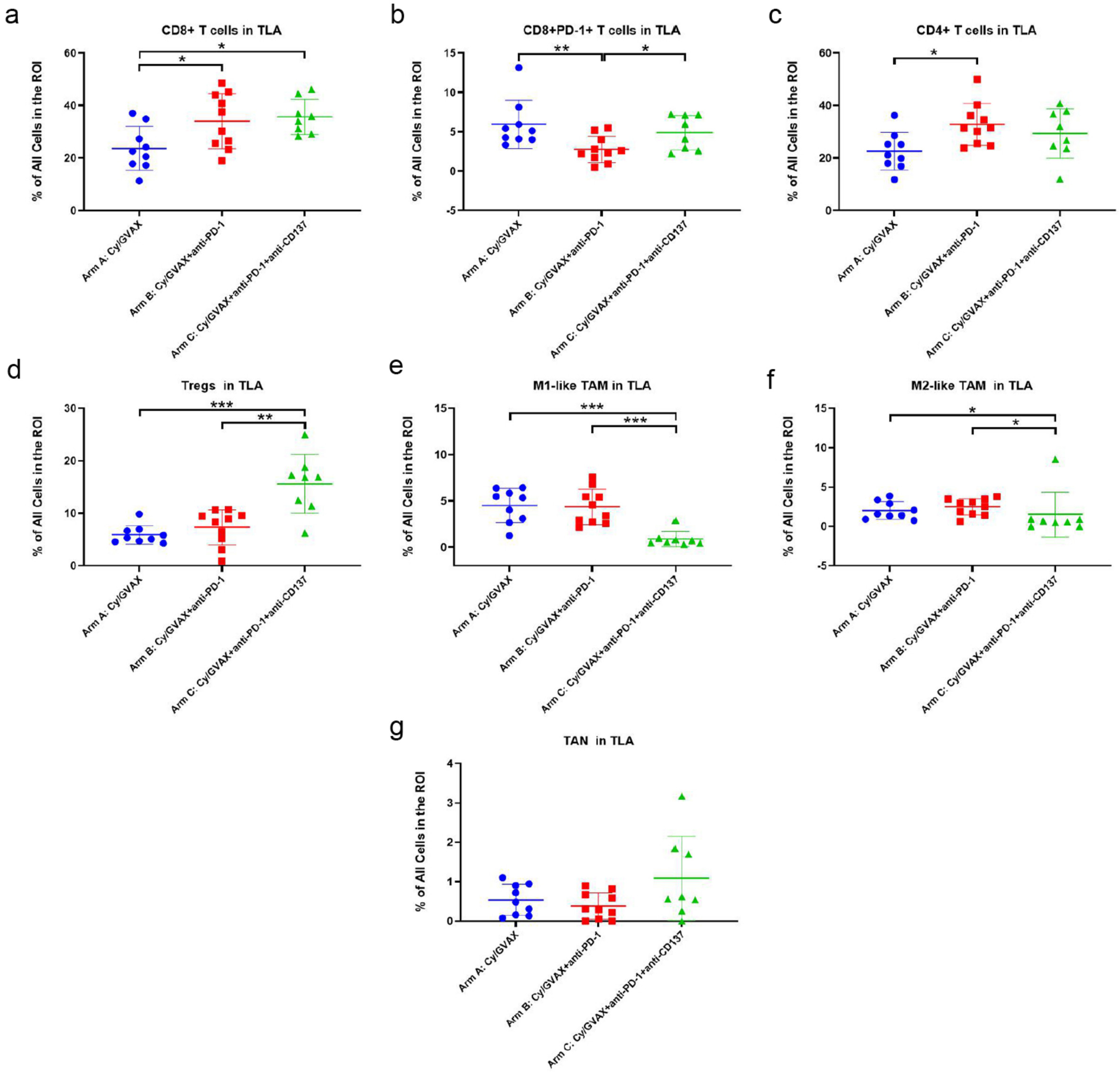
a
CD8+ T cells in TLA
b
CD8+PD-1+ T cells in TLA
c
CD4+ T cells in TLA
d
Tregs in TLA
e
M1-like TAM in TLA
f
M2-like TAM in TLA
g
TAN in TLA
% of All Cells in the ROI
Arm A: Cy/GVAX
Arm B: Cy/GVAX+anti-PD-1
Arm C: Cy/GVAX+anti-PD-1+anti-CD137

Supplementary Figure 9: Comparison of the Densities of Multiple Immune Cell Subtypes between Treatment Arms. [a] CD45+CD3+CD8+ T cells. GVAX (Arm A) vs GVAX+PD-1+CD137 (Arm C): p=0.0111; GVAX (Arm A) vs. GVAX+PD-1 (Arm B): p=0.0279. [b] CD45+CD3+CD8+PD-1+ T cells. GVAX (Arm A) vs. GVAX+PD-1 (Arm B): p=0.0076, GVAX+PD-1 (Arm B) vs. GVAX+PD-1+CD137 (Arm C): p=0.0434. [c] CD45+CD3+CD4+ T cells. GVAX (Arm A) vs. GVAX+PD-1 (Arm B): p=0.0133. [d] CD45+CD3+CD4+Foxp3+ T cells. GVAX (Arm A) vs GVAX+PD-1+CD137 (Arm C): p=0.0006, GVAX+PD-1 (Arm B) vs. GVAX+PD-1+CD137 (Arm C): p=0.0021. [e] CD45+CD3-CSF-1R+CD68+CD163- M1-like macrophages. GVAX (Arm A) vs GVAX+PD-1+CD137 (Arm C): p=0.0003, GVAX+PD-1 (Arm B) vs. GVAX+PD-1+CD137 (Arm C): p=0.0003. [f] CD45+CD3-CSF-1R+CD68+CD163+ M2-like macrophages. GVAX (Arm A) vs GVAX+PD-1+CD137 (Arm C): p=0.0149, GVAX+PD-1 (Arm B) vs. GVAX+PD-1+CD137 (Arm C): p=0.0152. [g] CD45+CD3-CD66b+ neutrophils. Treatment arms as indicated. [a-g] Arm A: n=9; Arm B: n=10; Arm C: n= 8. All data shown as the mean ± SD. Two-sided Mann Whitney tests were performed; p values were shown: *<0.05; **<0.01; ***<0.001; if not shown, non-significance. Treg, regulatory T cell; TAM, tumor associated macrophage; TAN, tumor associated neutrophil. Multiplex IHC analysis was repeated twice with consistent results.

Supplementary Table 1: Cox Proportional Hazard Model (Univariate) for Disease-Free and Overall Survival Tumor Tissue Covariates

		DFS		OS	
	**Frequency (%)**	**HR (95%CI)**	***p***	**HR (95%CI)**	***p***
**CD3+CD8+CD137+ T cells Density [ab]**					
≤0.41%[d]	12 (52.2)	Ref	-	Ref	-
>0.41%[d]	11 (47.8)	**0.30 (0.11-0.86)**	**0.026**	*0.61 (0.22-1.70)*	*0.349*
**CD3+CD8+GZMB+ T cells Density [bc]**					
≤2.1%[d]	14 (50.0)	Ref	-	Ref	-
>2.1%[d]	14 (50.0)	0.62 (0.26-1.50)	0.291	1.12 (0.45-2.76)	0.813
**CD3+CD8+CD137+GZMB+ T cells Density [ab]**					
≤0.01%[d]	13 (56.5)	Ref		Ref	
>0.01%[d]	10 (43.5)	0.41 (0.14-1.17)	0.095	0.41(0.13-1.29)	0.127

[a] The multiplex immunohistochemistry (mIHC) workflow was able to be performed on surgical specimens from 23 study patient: n=7 (Arm A), n=8(Arm B), n= 8 (Arm C); [b] Reflects an averaged proportion of T cell subtype within evaluated ROIs/TLAs per specimen. This was chosen instead of absolute numbers to reflect the proportion of this cell type of interest and to normalize comparisons between ROIs/TLAs within and across resected samples; [c] The multiplex immunohistochemistry (mIHC) workflow was able to be performed on surgical specimens from 28 study patient: n=10 (Arm A), n=10 (Arm B), n= 8 (Arm C); [d] Grouped by median across groups (above and below).

Supplementary Table 2: Cox Proportional Hazard Model for Disease-Free Survival for Clinical Covariates (Efficacy Cohort [N=40])

**Survival Association**		**Disease Free Survival**			
	**Frequency (%)**	**HR (95%CI) Univariate**	***p***	**HR (95%CI) Multivariate**	***p***
**Treatment Arm**					
Cy-GVAX	16 (40)	Ref		Ref	-
Cy-GVAX+PD1	14 (35)	1.09 (0.50-2.40)	0.829	1.33 (0.56-3.14)	0.517
Cy-GVAX+PD1+CD137	10 (25)	0.55 (0.21-1.49)	0.242	0.64 (0.19-2.19)	0.478
**Age at Surgery**					
<65 years	16 (40.0)	Ref	-	Ref	**-**
≥65 years	24 (60.0)	1.08 (0.53-2.20)	0.834	1.09 (0.51-2.35)	0.822
**Resection Status**					
R0	37 (92.5)	Ref	-		
R1	3 (7.5)	1.09 (0.26-4.62)	0.905		
**Lymph Node Involvement**					
No	12 (30)	Ref	-		
Yes	28 (70)	**2.44 (0.99-5.97)**	**0.052**	2.21 (0.88-5.53)	0.091
**Adjuvant SOC Chemotherapy Tx**					
Non-FOLFIRINOX regimen	24 (64.9)	Ref	-	Ref	-
(m)FOLFIRINOX	13 (35.1)	0.84 (0.38-1.86)	0.66	1.08 (0.40-2.93)	0.881

Supplementary Table 3: Cox Proportional Hazard Model for Overall Survival for Clinical Covariates (Efficacy Cohort [N=40])

**Covariate (Level)**	**Overall Survival**				
	**Frequency (%)**	**HR (95%CI) Univariate**	***p***	**HR (95%CI) Multivariate**	***p***
**Treatment Arm**					
Cy-GVAX	16 (40)	Ref	-	Ref	-
Cy-GVAX+PD1	14 (35)	1.11 (0.47-2.63)	0.813	1.84 (0.68-4.97)	0.231
Cy-GVAX+PD1+CD137	10 (25)	0.59 (0.18-1.91)	0.377	0.75 (0.18-3.10)	0.692
**Age at Surgery**					
<65 years	16 (40.0)	Ref	**-**	Ref	-
≥65 years	24 (60.0)	2.00 (0.88-4.57)	*0.100*	2.48 (0.97-6.35)	*0.059*
**Resection Status**					
R0	37 (92.5)	Ref	-		
R1	3 (7.5)	1.14 (0.27-4.88)	0.861		
**Lymph Node Involvement**					
No	12 (30)	Ref	**-**		
Yes	28 (70)	**2.88 (1.06-7.77)**	**0.037**	**2.92 (1.02-8.32)**	***0.045***
**Adjuvant SOC Chemotherapy Tx**					
Non-FOLFIRINOX regimen	24 (64.9)	Ref	-	Ref	-
(m)FOLFIRINOX	13 (35.1)	1.15 (0.46-2.88)	0.767	1.26 (0.41-3.87)	0.683

Supplementary Table 4: J1568 Arm C and Historical Control Patient Baseline Characteristics

	**Historical Cohort** ***Unmatched*** **(n=48)**	**Arm C (n=10)**	**Historical Cohort** ***Matched (3:1)*** **(n=30)**
**Age at Surgery**			
Median (Min, Max)	70.0 [45.0, 90.0]	70.0 [46.0, 83.0]	68.5 [51.0, 81.0]
<65 years	16 (33.3%)	4 (40.0%)	13 (43.3%)
≥65 years	32 (66.7%)	6 (60.0%)	17 (56.7%)
**Lymph Node Involvement**			
No	7 (14.6%)	3 (30.0%)	6 (20.0%)
Yes	41 (85.4%)	**7 (70.0%)**	24 (80.0%)
**Adjuvant SOC Chemotherapy Tx**			
Non-FOLFIRINOX regimen	14.0 (29.2%)	3 (30.0%)	9 (30.0%)
(m)FOLFIRINOX	34.0 (70.8%)	7 (70.0%)	21 (70.0%)

Note: Matching based on adjuvant chemotherapy regimen, age at surgery, and nodal disease status

Supplementary Table 5: Disease-Free Survival Comparisons Between J1568 Arm C and Matched Historical Control Cohort

			Disease-Free Survival (months)		
**Group**	**Median follow up (mo) [min, max]**	**Events**	**Median (95% CI)**	**Stratified HR (95% CI)**	**p-value**
**Matched Historical Control (3:1) (n=30)**	30.1 [4.80, 51.2]	21	20.83 (13.11, 33.08)	Ref	-
**Cy-GVAX PD1 CD137** (n=10)	30.5 [9.59, 38.9]	6	33.02 (16.33, NR)	0.72 (0.29-1.80)	0.480

Note: Cox regression, stratified by adjuvant chemotherapy ([m]FOLFIRINOX vs non-[m]FOLFIRINOX), was used to compare historical cohort vs study Arm C. Matching based on adjuvant chemotherapy regimen, age at surgery, and nodal disease status, Survival measured from date of surgery in both groups.

Supplementary Table 6: Summary of disease-free survival (DFS) and overall survival (OS) outcomes of J1568 in context of previous vaccine, immunotherapy, and historical benchmark adjuvant therapy trials in resected pancreatic adenocarcinoma.

**Study**	**n**	**Enrollment Period**	**Median DFS months (95% CI)**	**Median OS months (95% CI)**
J1568[a] Arm (NCT02451982) NA/Adj Cy-GVAX + Adj SOC	16	2016-2018[c]	13.90 (5.59, NR)	23.59 (13.27, NR)
J1568[a] (NCT02451982) NA/Adj Cy-GVAX+PD1+ Adj SOC	14	2016-2018[c]	14.98 (7.95, 44.09)	27.01 (20.76, NR)
J1568[a] (NCT02451982) NA/Adj GVAX(cy)+PD1+CD137+ Adj SOC	10	2019-2021	33.51 (16.76, NR)	35.55 (17.74, NR)
J0810[a] (NCT00727441) NA/Adj GVAX(+/-cy) + Adj SOC	66	2008-2015	12.51(9.74, 17.93)	19.4 (16.9, 28.5)
J9988[a] (NCT00084383) Adj GVAX + Adj SOC	60	2001-2004	17.27(14.5, 22.8)	24.8 (21.2, 31.6)
CITN11-01[a] (NCT02588443) NA/Adj CD40 + NA/Adj GnP	16	2015-2018	13.8 (2.9-24.8)<br>9.8 (0.4-19.2)	23.4 (18.0-28.8)<br>23.4 (9.1-37.6)
ESPAC-4[b]				
Gemcitabine + Capecitabine	364	2008-2014	13.9 (12.1, 16.6)	28 (23.5, 31.5)
Gemcitabine	366		13.1(11.6, 15.3)	25.5(22.7, 27.9)
PRODIGE-24[b]				
FOLFIRINOX	247	2012-2016	21.6 (17.7, 27.6)	53.5 (43.4, 58.4)
Gemcitabine	246		12.8 (11.7, 15.2)	35.0 (28.7 to 43.9)
APACT[b]				
Gem+Nab-Paclitaxel	432	2014-2018	19.4	41.8
Gemcitabine	434		18.8	36.2

[a] Measured survival beginning day of first study treatment; [b] Measured survival beginning day of trial randomization; [c] Includes one patient enrolled in 2021; Abbreviations: Adj=Adjuvant; CD40= anti-CD40 mAb agonist (Selicrelumab); CD137= anti-CD137 mAb agonist (Urelumab); Cy=Cyclophosphamide; Gem= Gemcitabine; GnP= Gemcitabine + Nab-Paclitaxel; GVAX= human granulocyte macrophage-colony stimulating factor (GM-CSF)-secreting whole-cell pancreatic cancer vaccine; NA=Neoadjuvant; PD1= anti-PD-1 mAb antagonist (Nivolumab); SOC= Standard of care chemotherapy or chemoradiation therapy

Supplementary Table 7: The multiplex IHC panel of myeloid and lymphoid markers.

Primary Ab	Hematoxylin	CD68	PD-1	Granzyme B	CD163
Clone	N/A	PG-M1	NAT105	EP230	10D6
Vendor	Dako	Abcam	Abcam	Sigma-Aldrich	Invitrogen
Cat#	S3301	ab783	ab52587	262R-1	MA5-11458
Concentration	N/A	1:50	1:50	1:100	1:100
Reaction	1 min	30 min	120 min	30 min	30 min
Secondary Ab	N/A	Anti-mouse	Anti-mouse	Anti-rabbit	Anti-mouse
Reaction		RT, 30 min	RT, 30 min	RT, 30 min	RT, 30 min
AEC reaction	N/A	40 min	180 min	120 min	30 min

Primary Ab	CD137	CD3	CD66b	CD45	CSF1R
Clone	BBK-2	SP7	G10F5	HI30	SP211
Vendor	Invitrogen	Invitrogen	Novus Biologicals	BD Bioscience	Abcam
Cat#	MA5-13739	MA5-14524	NB100-77808	555480	ab183316
Concentration	1:50	1:150	1:600	1:100	1:150
Reaction	120min	30 min	30 min	30 min	30 min
Secondary Ab	Anti-mouse	Anti-rabbit	Anti-mouse	Anti-mouse	Anti-rabbit
Reaction	RT, 30 min	RT, 30 min	RT, 30 min	RT, 30 min	RT, 30 min
AEC reaction	60min	100 min	30 min	30 min	20 min

Primary Ab	FOXP3	CD4	CD8	TIGIT	EpCAM
Clone	236A/E7	4B12	C8/144B	BLR047F	E144
Vendor	eBioscience	Invitrogen	eBioscience	Abcam	Abcam
Cat#	14-4777-82	MA5-12259	14-0085-82	ab243903	ab32392
Concentration	1:40	1:25	1:100	1:100	1:500
Reaction	30 min	30 min	30 min	60 min	60 min
Secondary Ab	Anti-mouse	Anti-mouse	Anti-mouse	Anti-rabbit	Anti-rabbit
Reaction	RT, 30 min	RT, 30 min	RT, 30 min	RT, 30 min	RT, 30 min
AEC reaction	120 min	30 min	30 min	25 min	25 min

Note: All antibodies were obtained commercially. These antibodies were tested and validated for their use in human samples by the respective supplier and by us via immunohistochemistry on the basis of the correct staining pattern. All antibodies had validation statement provided on the website of the manufacturer

A Platform Study of Combination Immunotherapy for the Neoadjuvant and Adjuvant Treatment of Patients with Surgically Resectable Adenocarcinoma of the Pancreas

Johns Hopkins Protocol #: J1568, IRB00050517
BMS Protocol #: CA209-423
ClinicalTrials.gov ID: NCT02451982

Principal Investigator: Lei Zheng, M.D., Ph.D.
[redacted]

IND Sponsor: Elizabeth Jaffee, M.D. [redacted] [redacted]
IND#: BB IND 27861

ClinicalTrials.gov ID: NCT02451982

Research Facility: Sidney Kimmel Comprehensive Cancer Center at Johns Hopkins
The Harry and Jeanette Weinberg Building
401 North Broadway, Baltimore, Maryland 21231-2410

Research Facility: The Johns Hopkins Oncology Center
600 N. Wolfe Street, Baltimore, MD 21287-7509

IRB: Johns Hopkins Medicine- Institutional Review Board
1620 McElderry Street, Reed Hall, Suite B-130
Baltimore, MD 21205-1911

Version:

Original/ Version 1/ April 16, 2015
Version 1.1/ May 14, 2015
Version 2/ July 20, 2015
Version 2.1/August 10, 2015
Version 2.2/September 30, 2015
Version 2.3/November 3, 2015
Version 3.1/December 1, 2015
Version 4.1/February 22, 2016
Version 5.1/June 24, 2016
Version 5.2/August 22, 2016
Version 5.3/October 11, 2016
Version 6/August 22, 2017
Version 7/December 18, 2017
Version 8/September 14, 2018
Version 8.1/October 18, 2018
Version 9/November 7, 2018
Version 10/August 15, 2019
Version 11/November 5, 2019
Version 12/February 20, 2020
Version 13/July 6, 2020
Version 14/September 30, 2020
Version 15/May 18, 2021
Version 15.1/September 27, 2021
Version 15.2 / January 21, 2022
Version 16.0 / February 23, 2022
Version 17.0 / July 8, 2022

Table of Contents

1.0 Synopsis 4

2.0 Background and Rationale 8

2.1 Introduction 8

2.2 Rationale for the GM-CSF-modified allogeneic tumor cell vaccine in pancreatic cancer immunotherapy. 8

2.3 Results of a Phase I study of an allogeneic GM-CSF-secreting tumor vaccine in patients with resected pancreatic cancer treated at Johns Hopkins Medicine (JHMI). 8

2.4 Results of a follow-up phase II study of the GM-CSF-secreting pancreatic tumor vaccine 9

2.5 Phase II Study of the GM-CSF allogeneic vaccine alone and given in sequence with immune modulating doses of IV Cyclophosphamide in subjects with advanced pancreatic cancer 10

2.6 Clinical study of ipilimumab vs. GVAX + ipilimumab for treatment of advanced unresectable PDA 11

2.7 The J0810 study of neoadjuvant and adjuvant GM-CSF allogeneic vaccine alone and given in sequence with immune modulating doses of IV or oral Cyclophosphamide in subjects with resectable pancreatic cancer 12

2.8 The Rationale of PD-1 blockade and/or CD137 agonist in combination with vaccine therapy for cancer immunotherapy 14

2.9 Prior Clinical and Preclinical Studies on Nivolumab, Urelumab, and BMS-986253 18

2.10 Rationale of Eligibility Criteria for Study Entry 20

3.0 Study Design and Treatment Plan 21

3.1 Study Design Overview 21

3.2 Study Plan Overview 21

3.3 Study population 27

3.4 Production and Administration of Study Drugs 32

3.5 End of Treatment visit 41

3.6 Follow-up phase 42

3.7 Treatment Discontinuation and Off study 42

3.8 Collection samples for correlative studies 43

3.9 Evaluation for safety and anticipated toxicities 44

4.0 Statistical Considerations 49

4.1 Sample size justification 49

4.2. Analysis of the primary efficacy endpoint 52

4.3 Analysis of safety and toxicity measurements 52

4.4. Analysis of OS and DFS 56

4.5 Statistical consideration for immune parameter evaluation 56
4.6 Analysis of Exploratory Endpoint 56
5.0 Response Criteria 57
5.1 Evaluation of clinical activity 57
5.2 Evaluation of immune parameters 57
6.0 Adverse Event Reporting 61
6.1 Definitions 61
6.2 Assessment of Causality 63
6.3 Expectedness 64
6.4 Handling of Expedited Safety Reports 64
6.5 Reporting 64
6.6 Special considerations for AEs that occur during the surgery and postoperative course 68
7.0 Clinical Trial Monitoring 71
8.0 References 73
APPENDIX A: Performance Status Criteria 76
APPENDIX B: Management Algorithms for Toxicities associated with Immune-Oncology (I-O) Therapies 77

1.0 Synopsis

Primary Objectives:

1) Arms A and B: To compare IL17A expression in vaccine-induced lymphoid aggregates between resected PDAs from patients treated with the combination of GVAX with low dose cyclophosphamide (Cy) and anti-PD-1 blockade antibody vs. the treatment of GVAX with low dose Cy alone.
2) Arms B and C: To compare the change of intratumoral CD8+CD137+ cells before and after neoadjuvant therapy between resected PDAs from patients treated with the combination of GVAX with low dose cyclophosphamide (Cy) and anti-PD-1 blockade antibody vs. the combination of GVAX with low dose cyclophosphamide (Cy), anti-PD-1 blockade antibody, and anti-CD137 agonist antibody.
3) Arm D: To assess the changes of intratumoral granzyme B+PD-1+CD137+ cells before and after neoadjuvant therapy from patients treated with the combination of anti-PD-1 blockade antibody and anti-IL8 blockade antibody.
4) Arm D: To assess pathologic response in resected PDAs of patients treated with the combination of anti-PD-1 blockade antibody and anti-IL8 blockade antibody.

Secondary Objectives:

1) To assess the safety of each of the immunotherapy study drug combinations
2) To assess overall survival (OS) of patients treated with each of the study drug combinations
3) To assess disease free survival (DFS) of patients treated with each of the study drug combinations
4) To assess and compare the effects of each of the immunotherapy study drug combinations on PD-L1/PD-1 associated pathways, vaccine-induced immune regulatory signatures, and peripheral and intratumoral antigen specific T cell responses.

Exploratory Objective:

1) To explore the effects of therapy on tumor and peripheral blood and tumor infiltrating immune cells, and to explore potential molecular determinants of response, progression and disease stability

Primary Endpoint(s):

Immunologic response

- Arms A and B, IL17A expression in vaccine-induced lymphoid aggregates
- Arms B and C, the change of intratumoral CD8+CD137+ cells before and after neoadjuvant therapy
- Arm D, the change in intratumoral granzyme B+ PD-1+ CD8+ T cells before and after neoadjuvant therapy
- Arm D: Pathologic response of resected tumors

Secondary Endpoint(s):

Safety
Disease free survival (DFS)
Overall survival (OS)
Immune parameters

Exploratory Endpoint:

Peripheral blood specimens, intratumoral core biopsy specimens, and resection specimens will be studied using a variety of laboratory techniques including but not limited to: immunohistochemistry (IHC), flow cytometry, cytokine/chemokine analysis, CITE-Seq, bulky or single cell RNA-Seq, whole exome sequencing, whole genome sequencing, exosome analysis, methylation analysis of ctDNA, T cell receptor and B cell receptor sequencing, ChIP-seq, ATAC-seq, and MBD-seq

Study Population:

In order to be considered for this study, patients need to meet the following criteria for inclusion:

- Have a newly diagnosed, biopsy-proven (or clinically suspected if the biopsy is not sufficient for diagnosis), surgically resectable, pancreatic adenocarcinoma of the head, neck, or uncinate process of the pancreas and is a candidate for the Whipple procedure.
- Have not received any cancer immunotherapy in the past

Number of Study Subjects:

Up to 76 patients will be enrolled in order to obtain 60 evaluable patients.

Total Evaluable Patients per arm:

Arm A (Cy/GVAX): 17

Arm B (Nivo/Cy/GVAX): 17

Arm C (Urelumab/Nivo/Cy/GVAX): 10

Arm D (BMS-986253/Nivo): 16

Study Design:

This is a single-institutional, randomized, open label platform clinical trial testing various immunotherapy combinations. Patients who have newly diagnosed and surgically resectable PDA and who will undergo the Whipple procedure at JHMI are eligible to participate in this study. Criteria for determining resectability will strictly follow NCCN guidelines.

The study consists of 6 parts. Parts 1-5 constitute the Prime Phase and Part 6 is the Extended Treatment Phase. See **Figure 1** for the treatment schema.

Part 1: Participants will receive one cycle of immunotherapy two weeks prior to undergoing the Whipple procedure.

Part 2: Subjects will undergo the Whipple procedure.

Part 3: Subjects will receive a second cycle of immunotherapy 4-10 weeks following the Whipple procedure (2-4 weeks prior to adjuvant chemoradiation).

Part 4: Subjects will undergo chemoradiation.

Part 5: Subjects will receive 4 additional 28-day cycles of immunotherapy beginning 1-2 months after completing chemoradiation for a total of six cycles (two before chemoradiation and 4 following chemoradiation).

Part 6: the Extended Treatment Phase:

- **Arm A** participants will receive Cy/GVAX every 12 weeks for another 2 doses.
- **Arm B and Arm C** participants will receive nivolumab every 4 weeks for another 6 doses and Cy/GVAX every 12 weeks for another 2 doses.
- **Arm D** participants will receive nivolumab every 4 weeks for another 6 doses.

Subjects are considered evaluable if they have an R0 or R1 resection of their tumors and their tumors are pathologically proved stage I/II adenocarcinoma of the pancreas. To achieve of 60 patients evaluable for the primary immunology endpoint, we estimate that approximately 76 eligible patients need to be consented and enrolled, according to an unevaluable rate of 12.5% in our prior neoadjuvant vaccine study. These patients are also considered evaluable for DSF and OS endpoints. All patients who receive any dose of protocol therapy will be monitored and evaluated for safety endpoints.

Eligible subjects will be randomized to Arms A and B in a 1:1 ratio, stratified up-front by age (≤65, >65), or enrolled directly to Arm C or Arm D. Enrollment to Arm C was completed on 8/25/2020. Enrollment to Arms A and B was closed early as of 11/4/2021 to prioritize enrollment to Arm D and future arms.

Due to the limited availability of Urelumab, enrollment and randomization for Arm A and Arm B was held until the enrollment for Arm C was completed. As of September 30, 2020, urelumab is no longer available and all patients remaining on Arm C after this date will receive the Arm B study regimen (same schedule and doses without urelumab).

The participants will be notified of their assigned arm after they have enrolled in the study. The first two patients in each arm will be enrolled and treated in a staggered fashion. For the first two staggered patients in each arm, the patients will be followed until the time of surgery or for 2 weeks, whichever occurs first following the neoadjuvant immunotherapy, before the next patient will be treated for the neoadjuvant immunotherapy.

Figure 1. Schema of the Treatment Plan

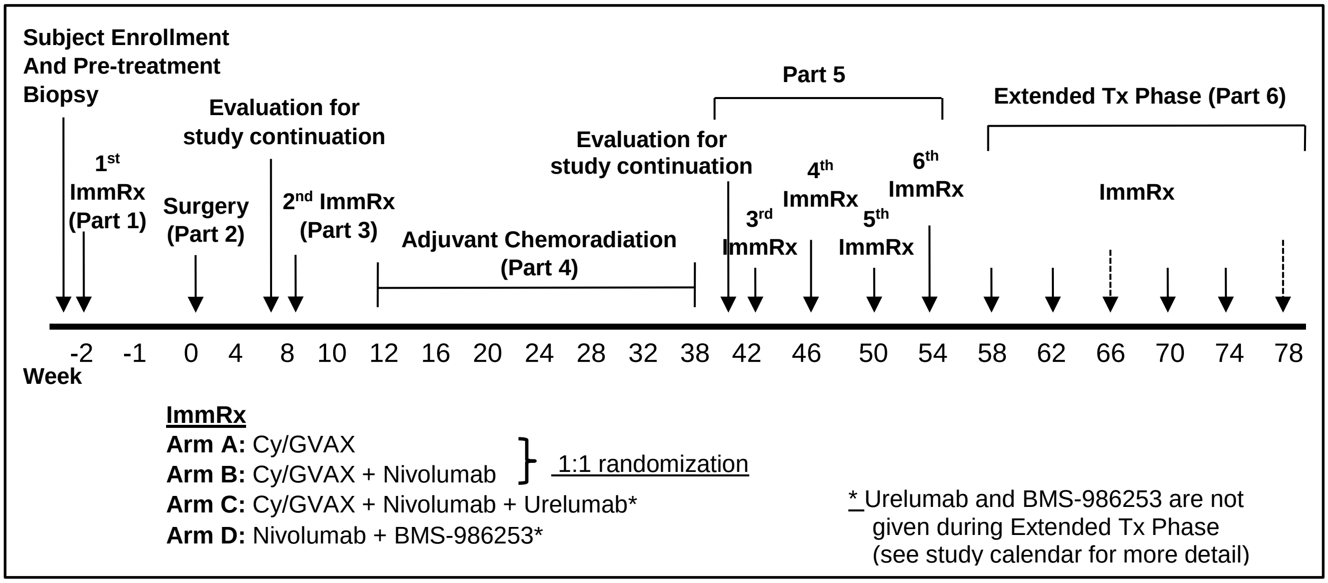

Treatments

Vaccine The vaccine consists of equal numbers (2.5 x 10^8 each) of Panc 6.03pcDNA1GM-CSF and Panc 10.05 pcDNA1GM-CSF combined into a single vaccination. Each of the vaccine components consists of a cultured, irradiated, allogeneic pancreatic tumor cell line that has been genetically modified with a plasmid vector encoding the cDNA for human GM-CSF. [redacted] Each vaccination will consist of six total intradermal injections, two each in the right and left thighs, and two in the non-dominant arm. In the event that the specified limb is contraindicated, the dominant arm may be used**.**

Cy (Cytoxan^R) The single intravenous (IV) dose of 200 mg/m^2 Cy is chosen based on our data showing that this single low IV dose given with a GM-CSF-secreting breast cancer vaccine is equivalent to the repetitive oral metronomic doses of Cy in reducing Treg levels in the PDA TME and facilitating enhanced T cell activation. Cy is a FDA-approved standard chemotherapy agent.

Anti-PD-1 Therapeutic Antibody OPDIVO ® or Nivolumab (BMS-936558; MDX-1106) is a potent and highly-selective monoclonal antibody that binds to the PD-1 receptor and blocks its interaction with PD-L1 and PD-L2, thereby releasing PD-1 pathway-mediated inhibition of the immune response, including anti-tumor immune response. It is FDA-approved for treating metastatic melanoma and squamous non-small cell lung cancer (NSCLC). The current recommended dose of nivolumab is 240 mg every 2 weeks or 480 mg every 4 weeks administered as an intravenous infusion over 30 minutes. In this study, nivolumab will be administered IV over 30 minutes at 480 mg approximately every 4 weeks.

Anti-CD137 agonist antibody Urelumab [redacted]

[redacted]

Anti-IL8 antibody [redacted]

Surgery and Chemoradiation Pancreaticoduodenectomy and chemoradiation is the standard of care and therefore is not part of the study.

2.0 Background and Rationale

2.1 Introduction

Pancreatic ductal adenocarcinoma (PDA) remains the fourth leading cause of cancer mortality in adults. Although 20-30% of patients are eligible for a pancreaticoduodenectomy, the reported median survival is only 13-20 months[1]. The addition of adjuvant radiation and/or chemotherapy has demonstrated limited improvement in survival[2]. New multidisciplinary therapeutic approaches are needed for all stages of this disease.

2.2 Rationale for the GM-CSF-modified allogeneic tumor cell vaccine in pancreatic cancer immunotherapy.

The use of whole-cell vaccines is promising because it delivers a range of peptide antigens without the need for specific knowledge of the relevant target antigens[3-12] ENREF 3 ENREF 3 ENREF 3 ENREF 3 ENREF 3 ENREF 3. Preclinical studies show that GM-CSF is the cytokine most effective in inducing anti-tumor immunity[3-12]. GM-CSF is an important growth and differentiation factor for dendritic cells, which are potent antigen-presenting cells (APCs) that can take up cellular proteins that encode for tumor antigens. The use of allogeneic tumor cells for vaccine development over autologous tumor cells is attractive for several reasons. Autologous tumor cells are unavailable or technically infeasible to produce. In addition, the characterization of tumor-associated antigens in melanoma revealed that most tumors share common antigens regardless of HLA type[3-12]. Furthermore, both preclinical and human data demonstrate that GM-CSF vaccine-induced host derived APCs rather than the tumor cells themselves prime $CD8^+$ T cells[3-12]. Importantly, we previously reported that the allogeneic GM-CSF pancreatic vaccine induced $CD8^+$ mesothelin specific T cells in patients who demonstrated prolonged disease-free and overall survival in phase I[13] and II testing[14]. Thus, the vaccine cells and the host do not have to be HLA compatible to prime effective $CD8^+$ T cell responses against pancreatic tumor antigens.

2.3 Results of a Phase I study of an allogeneic GM-CSF-secreting tumor vaccine in patients with resected pancreatic cancer treated at Johns Hopkins Medicine (JHMI).

This study was the first clinical trial to test the hypothesis that allogeneic GM-CSF secreting pancreatic tumor cell lines can prime a systemic immune response in patients with resected pancreatic adenocarcinoma[9]. Fourteen patients with stage 2 or 3 disease received an initial vaccination 8 weeks following resection. This was a dose escalation study in which patients each

received 10^7, $5X10^7$, 10^8, and $5X10^8$ vaccine cells. Study patients were jointly enrolled in an adjuvant chemoradiation protocol for 6 months, then given 3 additional vaccinations one month apart at the same original dose that they received for the first vaccination. Toxicities were limited to grade I/II local reactions at the vaccine site, and self-limited systemic rashes (**Table 1**). Systemic GM-CSF levels were evaluated as an indirect measure of the longevity of vaccine cells at the immunizing site. GM-CSF levels peaked at 48 hours following vaccination. The vaccine sites were also evaluated as a measure of the local immune reaction to the vaccine. Eleven of 14 patients demonstrated a local inflammatory response, similar to what has been observed in pre-clinical models and autologous GM-CSF vaccine clinical trials. Post-vaccination DTH responses to autologous tumor cells were observed in 1 of 3 patients receiving 10^8 and in 2 of 5 patients receiving $5X10^8$ vaccine cells.

Table 1. Toxicity Events Associated with Phase I Allogeneic Pancreatic Tumor Vaccine

	Grade 1†		Grade 2		Grade 3		Grade 4	
Toxicity*	No. of Vaccine Events	No. of Patients Who Had Toxicity	No. of Vaccine Events	No. of Patients Who Had Toxicity	No. of Vaccine Events	No. of Patients Who Had Toxicity	No. of Vaccine Events	No. of Patients Who Had Toxicity
Local								
Erythema at vaccine site	1^{a}	1	$26^{a,b,c,d}$	12				
Induration at vaccine site			$26^{a,b,c,d}$	12				
Pruritus at vaccine site	1^{b}	1	$18^{a,b,c,d}$	11				
Tenderness at vaccine site			2^{d}	1				
Recall induration at vaccine site	1^{d}	1						
Lymphedema of one extremity	1^{d}	1						
Systemic								
Pruritus, not at vaccine site			$3^{b,d}$	3				
Urticaria			1^{d}	1				
Skin rash			$2^{d,e}$	2				
Joint stiffness/pain	$2^{b,d}$	2						
Fatigue	1^{d}	1						
Other[f]					$1^{c,f}$	1	$1^{c,f}$	1

*Toxicities were graded using the National Cancer Institute's cancer clinical trials common toxicity criteria.

†The data represent the toxicities observed in the 14 patients who were treated with the vaccine. There were a total of 30 assessable vaccine treatments. Footnotes in table are defined as follows: a, occurred after dose level 1 (1×10^7 cells); b, occurred after dose level 2 (5×10^7 cells); c, occurred after dose level 3 (1×10^8 cells); d, occurred after dose level 4 (5×10^8 cells); e, one rash was biopsied to reveal Grover's syndrome; f, after receiving dose level 3 (1×10^8 cells), one patient experienced grade 2 thrombocytopenia, grade 2 elevated AST, grade 3 seizure, grade 3 anemia, grade 3 elevated ALT, and grade 3 elevated bilirubin. The symptoms were attributed to thrombotic thrombocytopenia purpura.

2.4 Results of a follow-up phase II study of the GM-CSF-secreting pancreatic tumor vaccine.

We completed a follow-up adjuvant study (study J9988) in 60 patients (88% lymph node positive) with operable pancreatic cancer[14]. Patients received an initial vaccination 8 weeks after pancreaticoduodenectomy, followed by chemoradiation, and then 4 more immunizations with the vaccine. Primary endpoints of this trial were to: 1) estimate the disease-free and overall survival benefit associated with this treatment; 2) further characterize the toxicities associated with the vaccine; 3) assess the induction of mesothelin-specific T cell responses and correlate with clinical response rates. With a follow-up of median follow up of 25.1 months, median disease-free survival is 17.3 months (95% CI: 13.4 – 19.1) with median survival of 24.8 months (95% CI: 21.2 - 31.6). A non-matched cohort analysis comparing patients to the Johns Hopkins Surgery database treated concurrently with similar adjuvant chemoradiation demonstrates an improvement for immunotherapy treated patients during the first 2 years of treatment.

Based on the analysis, the study concluded that the administration of the GM-CSF allogeneic cancer vaccine is safe and well tolerated. Treatment related side effects were similar to those side effects seen in the phase I study. The most common side effects were vaccine injection site reactions of induration and erythema that were transient in all research participants. In addition, some subjects also had transient vaccine injection site reactions of tenderness and pruritus. The systemic reactions included transient elevation in eosinophil counts, rashes and flu-like symptoms that have included low grade fever, chills, malaise, arthralgias, myalgias, and fatigue. Most patients had a transient elevation in their eosinophil count which demonstrates the bioactivity of GM-CSF. All vaccine related toxicities have been of the same intensity and duration as those observed in the phase I study[9].

2.5 Phase II Study of the GM-CSF allogeneic vaccine alone and given in sequence with immune modulating doses of IV Cyclophosphamide in subjects with advanced (Stage 4) pancreatic cancer

A feasibility study of the GM-CSF allogeneic vaccine administered alone or in sequence with Cyclophosphamide in subjects with advanced pancreatic cancer has been completed[15]. This study was an open label multi-center study sponsored by Cell Genesys, Inc in collaboration with US Oncology. Subjects were enrolled into one of two cohorts: Cohort A- 30 subjects administered a maximum of six doses of the same pancreatic cancer vaccine as described above using the two pancreas cancer cell lines each delivering 2.5 x 10^8 cells intradermally administered at 21 day intervals; Cohort B- 20 subjects administered cyclophosphamide 250 mg/m^2 IV one day prior to vaccine as in Cohort A. The primary objective was to evaluate the safety and induction of immune responses when treated with either vaccine alone or in sequence with cyclophosphamide. Secondary objectives include time to disease progression (TTP), median overall survival (OS), and assessment of the feasibility of detecting mesothelin-specific T cell responses in patients with advanced pancreatic cancer.

From this study, we concluded that the administration of a GM-CSF allogeneic pancreatic cancer vaccine is safe, feasible, and tolerated both alone and when given in sequence with cyclophosphamide. It was well-tolerated by patients with advanced pancreatic cancer, and the majority of these patients had received two or more prior chemotherapy regimens. The median number of vaccines administered was 2 in Cohort A and 3 in Cohort B. Treatment related adverse events reported in > 5% of subjects included local vaccine injection site reactions (100%), fever (14%), rigors (10%) and rash (6%). Grade 3/4 treatment related events identified in only one JHU subject and included leukocytosis, dehydration, and fatigue.

Stable disease was noted in 16.7 % of subjects in Cohort A (vaccination alone) and 40% of subjects in Cohort B (vaccination plus Cytoxan). Median survival in Cohort A and Cohort B were 2.3 months and 4.7 months respectively in a subject population that had received ≥ 2 prior chemotherapy in 12/20 subjects for Cohort B and in 30/50 subjects overall. This compares well with what is reported for first and second line therapy in this patient population. Furthermore, mesothelin-specific T cell responses have been observed in treated patients. Interestingly, unlike patients with resected cancer, mesothelin-specific T cell responses can be detected at baseline, prior to vaccination, in patients with metastatic pancreatic cancer. In addition, there was a trend toward prolonged progression-free survival in those patients who demonstrated persistent mesothelin-specific T cell responses with therapy. These data would suggest that even in metastatic patients, tumor-specific T cells can be detected.

This study represents the first demonstration that integrating immunomodulatory doses of Cy with a GM-CSF-secreting vaccine in patients with advanced pancreatic cancer is safe and feasible to administer. These data suggest that the vaccine given in sequence with Cy results in anti-tumor activity that is at least similar to gemcitabine-containing chemotherapy. In addition, mesothelin-specific $CD8^+$ T cell responses can be detected in stage 4 patients treated with the vaccine and may correlate with time to progression and overall survival. Thus, these findings provide the scientific rationale to continue to test combinations of vaccine with other more potent immune modifying agents.

2.6 Clinical study of ipilimumab vs. GVAX + ipilimumab for treatment of advanced unresectable PDA

The Phase 1b study of ipilimumab (IPI, anti-CTLA-4 blockade antibody) versus GVAX + IPI in advanced PDA represents the first clinical study of a checkpoint inhibitor in combination with a vaccine for PDA[16]. Thirty patients with previously treated PDAC were randomized 1:1 to IPI at 10mg/kg alone (arm 1) or in combination with GVAX (arm 2). Patients received 4 induction doses of IPI or GVAX/IPI at 3-week intervals and then maintenance with the same treatment every 3 months. CA19-9 declines in association with GVAX + IPI treatment were seen for 7/15 patients. In contrast, 0/15 patients receiving IPI alone had CA19-9 declines. Median overall survival (OS) was 3.7 months for arm 1 and 5.7 months for arm 2 (p=0.072). The percentage of patients alive after one year also favored the combination arm (7% vs 27%)[16]. The best RECIST response was stable disease (SD) in two patients in arm 1 and two patients in arm 2. Using the immune-related RECIST criteria (irRC), arm 2 had an additional patient with SD for 81 weeks. Immune-related response criteria (irRC) account for the kinetics of both old and new lesions given the known potential delayed responses with IPI. The quality of the responses in the two arms was different. Patients with SD on arm 1 had continuous disease progression that did not reach the 20% growth cutoff for 7 and 22 weeks. Arm 2 had three SD responses (one patient demonstrated a regression starting at week 14 that was maintained until week 31, another patient's disease stabilized starting at week 22 and was maintained for 81 weeks, and the third SD was maintained for 71 weeks while that patient was on study). The second patient initially received GVAX as a participant in the above mentioned neoadjuvant and adjuvant vaccine study. [redacted] This data, albeit anecdotal, suggests that the combination of checkpoint inhibitor and vaccine therapies may reverse an unfavorable TME that is dominated by immune suppressive signals and allow for the generation of a productive antitumor response. Nevertheless, IPI was associated with high grade including

immune-related adverse events (irAE); thus, a checkpoint inhibitor such as anti-PD-1 therapy that is associated with less frequent irAE and has the same efficacy as IPI has gained much interest.

2.7 The J0810 study of neoadjuvant and adjuvant GM-CSF allogeneic vaccine alone and given in sequence with immune modulating doses of IV or oral Cyclophosphamide in subjects with resectable pancreatic cancer

Between July 2008 and September 2012, 59 patients were enrolled into an ongoing study (NCT00727441, J0810) of an irradiated, allogeneic GM-CSF-secreting pancreatic tumor vaccine (GVAX) administered intradermally either alone or in combination with immune modulatory doses of cyclophosphamide (Cy) as neoadjuvant and adjuvant treatment for patients with resectable PDA. The immune modulatory role of low dose Cy in depleting regulatory T cells were demonstrated in a number of pre-clinical and clinical studies[17-24]. Most of these 59 patients were enrolled during a 24 months active enrollment period. Patients were randomized 1:1:1 to 3 treatment arms[25]. In Arm A, patients received GVAX alone; in Arm B, patients received GVAX plus a single intravenous dose of Cy at 200 mg/m^2 1 day prior to each vaccination; in Arm C, patients received GVAX plus oral Cy at 100 mg once daily for 1 week on and 1 week off _ENREF_36. Up to 6 GVAX treatments were administered and all of the patients remained in their initial treatment arms throughout the duration of the study. All 59 of the patients received the 1st GVAX treatment 2 weeks +/-4 days prior to surgery. Fifty-four patients successfully underwent pancreaticoduodenectomy (the Whipple surgery) and received the 2nd GVAX treatment. Eligible patients must have a mass in the head, neck and uncinate process of the pancreas suspected for adenocarcinoma by a multidisciplinary clinical trial team comprised of radiologists, surgical oncologists and medical oncologists. Biopsy would not be routinely required prior to surgical resection of a mass of pancreas suspected for PDA; therefore, biopsy was also not required at the entry of this vaccine study. Five patients were found intraoperatively to have liver metastases, which were not radiographically identified prior to surgery, and instead underwent a bypass surgery. Among 54 patients who had pancreaticoduodenectomy, 1 patient was found to have ampullary cancer, 1 to have neuroendocrine tumor, 2 to have undifferentiated carcinoma, and 1 to have autoimmune pancreatitis. These patients' preoperative CT scans did not distinguish their disease process from PDA. In addition, 1 patient had grossly residual tumors and another 11 patients had recurrence immediately following the surgery. They were all taken off the study postoperatively. The 39 patients remaining on the study received standard adjuvant chemotherapy and radiation therapy. Patients remaining disease-free following chemoradiation therapy received up to 4 additional PDA GVAX treatments every 4 weeks.

This study demonstrated that it is safe to treat patients suspected to have PDA with pancreatic GVAX in the neoadjuvant setting, including patients who end up not having PDA. The sample size of this study was later increased to 87 in order to generate more preliminary data to support future research directions including the current application; and by far, it has completed the enrollment of 81 of the 87 patients. Thus, patients' clinical outcome data in this study have not matured at the time of submitting this application. The sample size of each treatment arm was not powered for the clinical efficacy analysis. We also do not anticipate that one additional vaccination in the neoadjuvant setting would significantly change patients' clinical outcome comparing to our prior study (clinical study J9988) of treating the patients with vaccines in the adjuvant setting[14]. An interim preliminary analysis did show that the DFS and OS of patients in this neoadjuvant and adjuvant study is similar to our prior J9988 adjuvant vaccine study. More importantly, as described

above and below, this neoadjuvant and adjuvant vaccine study has pioneered the neoadjuvant research approach for cancer immunotherapy and supported the use of the same neoadjuvant approach for the study of the combination of anti-PD-1 therapy and vaccine therapy.
Furthermore, the primary objective of this prior study was to analyze the effects of treatment on the tumor microenvironment (TME). Pathologic examination of resected PDAs revealed the formation of vaccine-induced intratumoral tertiary lymphoid aggregates within two weeks following a single GVAX vaccine treatment, regardless of whether GVAX was combined with Cy or not. Gene microarray analysis of microdissected vaccine-induced lymphoid aggregates identified gene signatures representing five signaling pathways including the NF-kB, Treg/TH17, chemokine, integrin/adhesion, and ubiquitin-dependent proteosome pathways. Gene expression and immunohistochemistry analyses further demonstrated that the Treg pathway is suppressed and the TH17 pathway is enhanced in lymphoid aggregates from patients who survive more than 3 years, in patients who demonstrate vaccine-enhanced mesothelin-specific T cell responses, and in patients with increased Teffector/Treg (CD8/Foxp3) ratios in their tumors. Overall, this study showed for the first time that GVAX-based immunotherapy can convert an immunologically inactive TME into an immunologically active TME; and that GVAX induces the formation of intratumoral tertiary lymphoid aggregates that facilitate a TH17-dominated anti-cancer response within the PDA TME following immunotherapy treatment.

The primary objective is to compare IL17 expression in vaccine-induced lymphoid aggregates between resected PDAs from patients treated with the combination of GVAX/Cy and anti-PD-1 antibody vs. GVAX/Cy alone. Our prior studies showed that higher IL17A expression in lymphoid aggregates was associated with longer OS in patients who received neoadjuvant and adjuvant GVAX (**Figures 4-6, Lutz et al.[25]**). This is the strongest biomarker identified through this prior study. Published studies have suggested that PD-1 blockade enhances TH17 response in patients with melanoma and prostate cancer. Therefore, we hypothesize that anti-PD-1 therapy will enhance IL17A expression in vaccine induced lymphoid aggregates. We are also going to analyze other immune parameters as part of explorative endpoints.

Consistent with the induction of an adaptive immune response, treatment with GVAX induced interferon gamma (IFNγ)-production in Teffs infiltrating PDAs, but also induced the upregulation of immunosuppressive regulatory mechanisms, including upregulation of the PD-1/PD-L1 pathway[25]. At baseline, only a small fraction of PDA epithelial tumor cells express low levels of membranous PD-L1. By contrast, GVAX therapy induced moderate expression of membranous PD-L1 on the epithelial tumor cells, and also induced the infiltration of innate immune cells expressing high levels of PD-L1 into the intratumoral lymphoid aggregates (**Figure 3** below and also **Figure 2** in Lutz et al.[25]). PD-L1 expression may be regulated by oncogenic pathways. However, in most cancers, PD-L1 is induced by cytokines produced by infiltrating immune cells during the induction of an adaptive immune response, such as IFNγ[25]. In melanoma, NSCLC and renal cell carcinoma, PD-L1 expression by tumor cells has been observed in approximately 53-89% of untreated patients' tumors and by tumor infiltrating immune cells in approximately 50-100% of tumors[26]. PD-L1 expression by both tumor cells and tumor infiltrating immune cells in untreated patients with these cancers is associated with PD-1 expression in tumor infiltrating lymphocytes (TILs), more abundant infiltration of immune effector cells, and the presence of

lymphoid aggregates. The high prevalence of immune cell infiltration and PD-L1 expression in these particular malignancies may explain their relatively high response rates to single therapy with anti-PD-1 or anti-PD-L1. By contrast, PDA demonstrates a minimal response to anti-PD-1/PD-L1 single therapies, that is likely due to the absence of immune effector cell infiltration and low PD-L1 expression in vaccine-naïve PDAs. However, we hypothesize that by inducing immune cell infiltration and PD-L1 expression in the TME, GVAX therapy primes the PDA TME for anti-PD-1/PD-L1 therapies. Therefore, in this application, we will test this hypothesis through a novel clinical trial designed to test the combination of GVAX and anti-PD-1 antibody in both neoadjuvant and adjuvant settings in patients with resectable PDA.

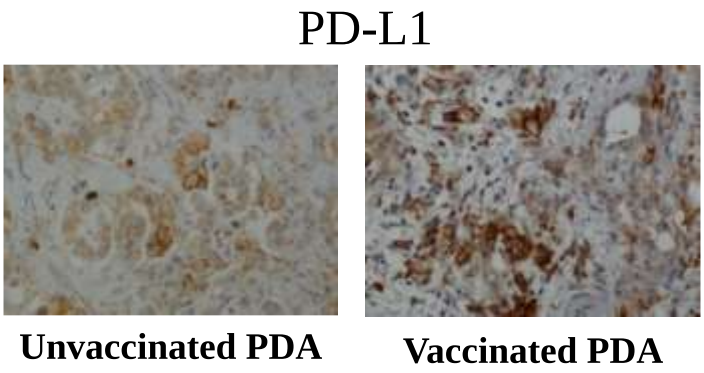

Figure 3. Immunohistochemistry staining of PD-L1 on PDA from patients who received or who did not receive GVAX treatment prior to surgical resection.

The significance of this neoadjuvant and adjuvant vaccine study is that it has pioneered the neoadjuvant treatment approach for evaluating cancer immunotherapy and supports the use of the same neoadjuvant approach to studying the combination of cancer immune checkpoint inhibitor and vaccine therapy.

2.8 The Rationale of PD-1 blockade and/or CD137 agonist in combination with vaccine therapy for cancer immunotherapy

The Rationale of PD-1 blockade in combination with vaccine therapy for cancer immunotherapy

It is clear that the signals generated solely by TCR recognition of antigens are insufficient to activate T cells to an effector state. In fact, when T cells receive the only signal 1 through TCR engagement without additional co-stimulatory signals, they enter an unresponsive or anergic state. The positive regulatory effects of co-stimulatory signals are balanced by the presence of a number of co-inhibitory molecules. Although the binding of B7-1 and B7-2 to their CD28 receptor on T cells provide co-stimulatory signals, they can act as co-inhibitors when they bind to the cytotoxic T lymphocyte antigen 4 (CTLA-4) on T cells. The latter provides a co-inhibitory signal and decreases T cell activation both by outcompeting CD28 for ligand binding and inhibiting the signaling cascade that would be activated through the B7-1/B7-2-CD28 axis. The discovery of

this immune regulatory mechanism establishes the concept of immune checkpoints. CTLA-4 is the prototype of the molecules that govern immune regulation. It is likely that immunologic checkpoints serve two biological purposes[27]. One helps generate and maintain self-tolerance among T cells specific for self-antigens. The other restrains the amplitude of normal T cell responses so that they do not "overshoot" in their natural response to foreign pathogens. The same immunologic checkpoint also gives tumor cells a chance at immune evasion. During tumor development, however, the balance leans toward co-inhibitory signals; and the presence of checkpoints plays a crucial role in the establishment of immune tolerance to tumors.

As discussed above, T cells harbor a natural co-inhibitory axis such as B7-1/B7-2-CTLA-4 that interacts with professional APC systemically. In addition to these systemic signals, T cells also express co-inhibitory signaling pathway that interact with tumor cells and other cells within the tumor's microenvironment. B7-H1 (PD-L1), another member of the B7 family, is an example of a co-inhibitory signal found on both DC and on many mouse and human tumor types[27]. Although resting T cells, B cells, and monocytes do not express B7-H1, they express high level of B7-H1 on their cell surface following activation. In contrast, DCs constitutively express B7-H1. Many types of tumor cells have been shown to have increased expression of B7-H1; and the tumor microenvironment can also stimulate the expression of B7-H1 on regulatory DCs. B7-H1 also has a close homolog, B7-DC (PD-L2), also in the B7 family. Expression of B7-DC appears to be restricted to DCs and monocytes. B7-DC also appears to be a co-inhibitory molecule. Both B7-H1 and B7-DC are ligands of PD-1, which is another inhibitory regulator expressed on the T cell surface[27].

PD-1 shares significant homology with CD28, the receptor of co-stimulatory signals, B7-1 and B7-2[27]. Its expression is induced upon activation of $CD4^+$ and $CD8^+$ T cells, B cells, and monocytes. PD-1 ligation to B7-H1/B7-DC causes inhibition of T cell activation and proliferation, which results in cell cycle arrest without apoptosis. The phenotype of PD-1 knockout mice is characterized by organ-specific autoimmunity. PD-1 is particularly expressed by tumor-associated T cells, a significant fraction of which are regulatory T cells. Studies suggest that these tumor-associated, PD-1 expressing T cells can suppress antitumor immunity. So far, these PD-1 expressing tumor-associated T cells, through co-inhibitory signaling via B7-H1, have been shown to suppress IL-12 production by myeloid DCs, thus counteracting the positive effect of co-stimulatory signals. However, blocking B7-H1 has been shown to enhance myeloid DC-mediated T cell activation, allowing for suppression of growth of ovarian carcinoma xenografts following adoptive transfer of these cells into mice. Administration of monoclonal antibodies (mAb) against PD-1 and B7-H1 has produced CTL-mediated antitumor effects in mice. Therefore, tumor-associated PD-1 expression on T cells represents an additional mechanism of tumor evasion when B7-H1 is expressed by the progressing tumor[27].

PD-1 blockade monoclonal antibodies are therefore promising agent under clinical development that targets an immune checkpoint. This antibody is expected to have a lower toxicity profile than anti-CTLA antibody. This is based on the result of PD-1 knockout mice developing mild strain-dependent, organ-specific autoimmunity, in contract to CTLA-4 knockout mice that develop lethal multi-organ autoimmunity. Second, it may have a relatively specific role in blocking T cell suppression in the tumor microenvironment. In contrast to the CTLA-4 ligands which are systemically expressed on APCs B7-H1, the PD-1 ligand is highly expressed in a variety of human tumors including pancreatic cancer.

Immune checkpoints targeting agents alone are not ideal treatment strategies for pancreatic cancer or perhaps for any other type of cancer. These agents would only provide immune modulation in a non-specific manner without direct activation of the relevant antigen-specific T cells. On the other hand, vaccines activate tumor-specific T cell immunity. If the vaccine approach is combined with immune modulators, the combinatorial therapy may have a synergistic effect on antitumor T cell activation. Indeed, such a synergy is supported by several preclinical studies. On another hand, the safety of cancer vaccines is supported by most studies that have been so far conducted. The combinatorial therapy is not expected to add any toxicity to that already observed with either agent. More importantly, combinatorial synergy with blockade of PD-1 and the vaccination has been demonstrated in the preclinical model.

The combination of anti-PD-1/PD-L1 therapies and vaccine therapy was tested by our group in a physiologically relevant mouse liver metastasis model of PDA. Untreated mouse PDA liver metastases that form following hemisplenic injection of panc02 cells do not express PD-L1. Not surprisingly, anti-PD-1/PD-L1 therapies alone did not significantly alter immune cell infiltration into the TME of the panc02 liver metastases (**Figure 4**). In the contrast, vaccine therapy moderately enhanced the infiltration of T effector cells into TME (**Figure 5**) and also induced PD-L1 expression on Panc02 tumor cells. When the two therapies were combined, anti-tumor effector T cell responses in the TME were significantly enhanced. The combination of vaccine and anti-PD-1/PD-L1 also increased the cure rate of tumor-bearing mice and prolonged the survival of the mice compared to either single therapy alone (**Figure 6**). This unpublished data suggests that vaccine therapy is critical for priming the PDA TME by inducing T effector cell infiltration, but also that additional immune modulation, such as anti-PD-1/PD-L1 therapy, is required to counter adaptive immune resistance mechanisms; and that the combination is necessary to induce an effective anti-tumor immune response. Finally, these preclinical data support the testing of the combination of anti-PD-1 antibody and GVAX for treatment of PDA in a clinical trial.

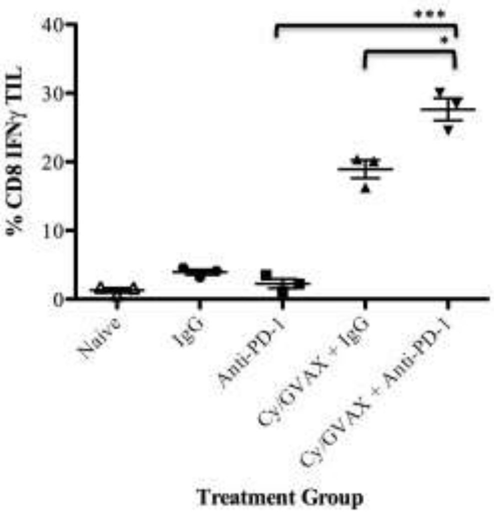

Figure 4. The percentage of IFNγ[+] producing CD8[+] T cells amongst all CD8[+] T cells in tumor infiltrating lymphocytes in the Panc02 liver metastases. Tumor-bearing mice were treated Cy, GVAX or αPD-1/αPD-L1 therapy as indicated.

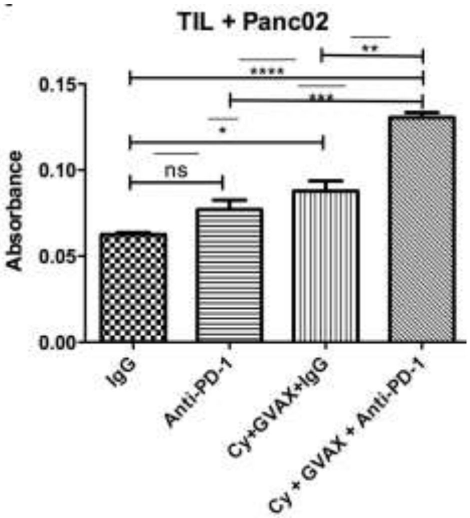

Figure 5. ELISA assays were performed using autologous irradiated Panc02 tumor cells as antigenic targets for CD8[+] T cells isolated from *TILs*. *p<0.05, ** p<0.01, *** p< 0.001,

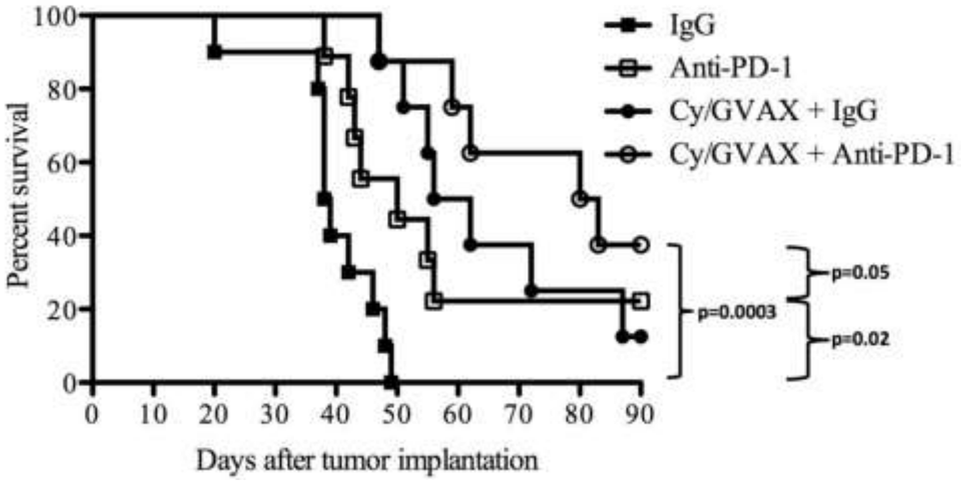

Figure 6. Kaplan-Meier analysis of percentages of mice that remained disease free at day 90 following Panc02 tumor implantation and that were treated with different combinations of Cy, GVAX and the αPD-1 antibody.

The Rationale of PD-1 blockade and CD137 agonist in combination with vaccine therapy for cancer immunotherapy

[redacted]

[redacted]

[redacted]

Rationale for PD-1 blockade in combination with anti-IL-8 for cancer immunotherapy

[redacted]

2.9 Prior Clinical and Preclinical Studies on Nivolumab, Urelumab, and BMS-986253

2.9.1 Prior Clinical and Preclinical Studies on Nivolumab (*OPDIVO®;* BMS-936558; MDX-1106)

Programmed cell death (PD)-L1 expression has been found on a number of tumors, and may be a mechanism by which tumors can directly engage PD-1 to evade an effective anti-tumor immune response. Expression of INF-y by T cells is known to induce PDL-1 expression in tumors. Single-agent nivolumab has anti-tumor activity with a complete response (CR) or partial response (PR) in subjects with NSCLC, melanoma, RCC). The majority of responses were durable and exceeded 6 months[28-31].

The overall safety experience with nivolumab, as a monotherapy or in combination with other therapeutics, is based on experience in more than 15,000 subjects treated to date. For monotherapy, the safety profile is similar across tumor types. The only exception is pulmonary inflammation adverse events (AEs), which may be numerically greater in subjects with NSCLC, because in some cases, it can be difficult to distinguish between nivolumab-related and unrelated causes of pulmonary symptoms and radiographic changes. There is no pattern in the incidence, severity, or causality of AEs to nivolumab dose level.

- Overall, to date, the safety profile of nivolumab was manageable and generally consistent across completed and ongoing trials with nivolumab monotherapy, with no maximum tolerated dose (MTD) reached at any dose tested up to 10 mg/kg.
- Most AEs were low-grade (Grade 1 to Grade 2), with relatively few related high grade (Grade 3 to Grade 4) AEs including acute renal failure, fatigue, diarrhea, pneumonitis, and increased aspartate aminotransferase (AST)/alanine aminotransferase (ALT). Most high-grade events were manageable with use of corticosteroids or hormone replacement therapy (endocrinopathies) as instructed in management guidelines.

2.9.2 Prior Clinical and Preclinical Studies on Urelumab (BMS-663513)

[redacted]

[redacted]

2.9.3 Prior Clinical and Preclinical Studies on anti-IL-8 antibody (BMS-986253)

[redacted]

[redacted]

[redacted]

[redacted]

[redacted]

2.10 Rationale of Eligibility Criteria for Study Entry

The evaluation of the patients for eligibility criteria of study entry is consistent with the standard care of pancreatic cancer. A diagnostic biopsy prior to the Whipple procedure for a patient with a suspected, resectable adenocarcinoma of the pancreas is a standard of care. For this study, a sample of tissue prior to treatment will be required for immunologic analysis comparing pretreatment and post-treatment cancer tissue. Biopsy carries 1-2% risk of causing pancreatitis. The driver mutations in the Kras oncogene occur in the majority (more than 95%) of pancreatic adenocarcinomas. One of the major correlative immunology studies of this clinical trial is to investigate the T cell response to the mutated Kras neoepitopes. Therefore, patients whose resected pancreatic adenocarcinomas carry Kras mutations are considered evaluable for the primary

(immunology) endpoint. Patients whose resected pancreatic adenocarcinomas do not carry Kras mutations will continue the study treatments and will be evaluated for other endpoints if they otherwise meet all the eligibility criteria.

3.0 Study Design and Treatment Plan

3.1 Study Design Overview

This platform trial will evaluate various immunotherapy combinations given in the neo-adjuvant and adjuvant setting in patients with surgically resectable pancreatic ductal adenocarcinoma.

3.2 Study Plan Overview

3.2.1 Part 1: Pre-study screening, consent, randomization, and neoadjuvant immunotherapy (Cycle 1)

Patients who will undergo the pancreaticoduodenectomy at the Johns Hopkins Medicine will be informed about this trial. Patients who are interested in participating will need to have a surgically resectable adenocarcinoma of the head, neck, or uncinate of the pancreas. The pre-surgical staging of disease will be performed by Department of Surgery. Pretreatment biopsy is necessary for enrollment in this study. If the patient is referred to the study with a diagnostic biopsy without a core biopsy specimen available through archive for the research purpose, a research core biopsy will be performed. If the patient does not have a diagnostic biopsy, the research biopsy will be done at the same time as the diagnostic biopsy. If a patient is found to be eligible for this study based on pre-surgical staging and pre-study screening, they will be consented and fully screened for this study. The first combinatorial therapy will be given two weeks +/- 4 days prior to the date of scheduled surgery.

3.2.2 Part 2: Surgery

Patients will undergo the pancreaticoduodenectomy at the Johns Hopkins Medicine. The surgical procedure performed will result in either a R0, R1 or R2 resection as determined by the operating surgeon. A portion of the resected tumor will be collected for tumor tissue banking (see section 3.8.2). The Department of Surgery also has a separate active IRB approved protocol (separate from the pancreatic cancer vaccine studies) for tumor acquisition. Information regarding any surgical therapy will be recorded including the operation performed, whether vascular resection and reconstruction was required, completeness of the resection (R0, R1 or R2), duration of the operation, blood loss, the length of stay, the need for re-admission within 30 days of surgery, and intraoperative and postoperative complications. Pancreaticoduodenectomy is the standard of care to which immunotherapy is added.

3.2.3 Part 3: Post Surgery Immunotherapy (Cycle 2)

Participants will be scheduled for the first post-surgery appointment between 4 and 8 weeks following surgery. Those patients who meet criteria for study continuation (Section 3.3.3) will return between 4 and 10 weeks following surgery to receive their second combinatorial therapy of the same regimen as their first vaccination cycle. Cycle 2 will be 28 days long for Arms A – C and 14 days long for Arm D.

3.2.4 Part 4: Adjuvant Combined Radiation and Chemotherapy

Following the second 28- or 14-day immunotherapy cycle, patients will start a 26-28 week course of adjuvant therapy consisting of combined chemotherapy and local radiation. 5-FU/Capecitabine/Gemcitabine-based chemotherapy and radiation therapy as adjuvant treatment following surgical resection of pancreatic adenocarcinoma are the accepted standard of care as described by a number of pancreas cancer groups including the RTOG, the American College of Surgeons Oncology Group (ACOSOG) and the National Comprehensive Cancer Network (NCCN) pancreatic adenocarcinoma practice guideline expert panel. Therefore, chemotherapy and radiation therapy are part of the standard of care to which immunotherapy is added.

3.2.5 Part 5: Post Chemoradiation Immunotherapy (Cycles 3-6)

Four to eight weeks following the completion of the last cycle of adjuvant radiation and chemotherapy, those patients who meet criteria for study continuation will receive four additional cycles at four-week intervals.

3.2.6 Part 6: Extended Treatment Phase

Following the sixth cycle, the participants will enter the Extended Treatment Phase. The Extended Treatment Phase is composed of two cycles of immunotherapy. Each extended treatment cycle is 12 weeks.

Patients on Arms B/C/D will receive nivolumab every 4 weeks for 3 doses per cycle (6 doses total). Patients on all arms except Arm D will receive one dose of Cy/GVAX per cycle, the first extended treatment vaccine (vaccine 7) will be 12 weeks following the 6th prime vaccination and a second (vaccine 8) 12 weeks later.

As this Extended Treatment Phase was added in an amendment, patients who had already completed 6 vaccinations and an end of treatment visit prior to the amendment's IRB approval remain in follow-up and will not receive the additional extended treatment doses.

3.2.7 Part 7: Follow-up phase

Patients will have an End of Treatment (EOT) visit 4 weeks after their last dose of study drug. Once the EOT visit is completed, subjects will enter the follow-up phase of the study. The follow-up phase will consist of contact with the patient (by visit, phone call, or email) or contact with the patient's local provider (if known) to evaluate disease status and survival once every three months for the first 24 months and then every six months for another 12 months or until the study closes. Patients in Arms B/C/D will be followed for SAEs that occur within 100 days of last dose of (non-GVAX) immunotherapy study drug.

3.2.8 Study Schedule Checklists

Table 2A Study Schedule of Immunotherapy Cycle #1

	Pre-Study	First cycle (Day 0-Day 14)						
	**Day -21 to Day 0**	**0**	**1**	**2-7**	**8**	**9-13**	**14**	**15**
**Eligibility assessment**								
Informed consent	X[1]							
Inclusion/Exclusion	X							
Medical history	X							
Pregnancy test	X[2]							
Performance status	X							
**Safety assessment**								
Vital signs[3]	X	X	X					
Physical exam, weight	X							
Toxicity assessments/ adverse events			X				X[11]	
**Laboratory test**								
Hematology[4]	X				X[11]		X[11]	
Comprehensive[5]	X				X[11]		X[11]	
CA 19-9	X							
Amylase/lipase	X				X[11]		X[11]	
TSH[6]	X						X[11]	
EUS guided pancreatic tumor core biopsy	X							
Peripheral blood draw for PBMC, CTC, plasma, and serum (up to 145 cc) [7]	X						X[11]	
Pathology review[8]	X							
**Efficacy Assessments**								
CT chest/abd/pelvis[9]	X							
**Treatment**								
IV cyclophosphamide (Arms A/B/C only)		X						
BMS-986253 (Arm D only)		X						
Nivolumab (Arm B/C/D only)		X						
Urelumab (Arm C only)		X						
Vaccine (Arms A/B/C only)			X[10]					
Surgery								X

In order to minimize the need for research-only in-person visits, telemedicine visits may be substituted for in-person clinical trial visits or portions of clinical trial visits where determined to be appropriate and where determined by the investigator not to increase the participants risks. Prior to initiating telemedicine for study visits the study team will explain to the participant, what a telemedicine visit entails and confirm that the study participant is in agreement and able to proceed with this method. In the event telemedicine is not deemed feasible, the study visit will proceed as an in-person visit. Telemedicine visits will be conducted using HIPAA compliant method approved by the Health System and within licensing restrictions.

1. To include enrollment in long term follow-up study. Consent may be performed up to 30 days prior to start of study treatment.

2. Pregnancy tests will be administered to WOCBP: a serum pregnancy test is required at screening; urine pregnancy tests are required within a window of up to 4 days prior to nivolumab administration for patients on Arms B/C/D.

3. Height and pulse oximetry measured at screen only. Blood pressure, pulse, and temperature will be measured on Day 0 any time prior to infusion and on Day 1 pre- and post-GVAX administration.

4. Heme-8 with differential including absolute eosinophil count, absolute neutrophils, absolute lymphocytes

5. Comprehensive chemistry panel including electrolytes, BUN, creatinine, AST, ALT, total bilirubin, alkaline phosphatase.

6. Total T3 and free T4 if TSH abnormal

7. Research samples will be collected at the discretion of the PI based on availability of supplies and safety of patient and staff. Detailed instructions for blood collection, processing & storage are provided in the Lab Manual

8. Only if the pathology of the biopsy is available.

9. CT of chest/abd/pelvis with iv contrast as per Institutional standard, e.g. pancreatic protocol at Johns Hopkins.

10. Vaccine given 2 weeks +/- 4 days prior to surgery·

11. Day 8 safety labs may be done within a -1/+3 day window. Day 14 / pre-surgery labs can be done on the day prior to surgery or within prior 3 days.

Table 2B Study Schedule of Immunotherapy Cycle #2

	Pre-Cycle#2	Second cycle							
	Day -14 to Day 0	0	1	2-7	8	9-13	14	15-27	28
**Eligibility assessment**									
Inclusion/Exclusion for study continuation	X								
Medical history	X								
Pregnancy test	X[1]								
Performance status	X								
**Safety assessment**									
Vital signs[2]	X	X	X						
Physical exam, weight	X								
Toxicity assessments/ adverse events			X				X[10]		X[11]
**Laboratory test**									
Hematology[3]	X				X[9]		X[10]		X[11]
Comprehensive[4]	X				X[9]		X[10]		X[11]
CA 19-9	X								
Amylase/lipase	X				X[9]		X[10]		X[11]
TSH[5]	X						X[10]		X[11]
Peripheral blood draw for PBMC, CTC, plasma, and serum (up to 145 cc) [6]	X						X[10]		X[11]
HLA typing (10 cc)	X								
Pathology review	X								
**Efficacy Assessments**									
CT chest/abd/pelvis[7]	X								
**Treatment**									
IV cyclophosphamide (Arms A/B/C only)		X							
BMS-986253 (Arm D only)		X							
Nivolumab (Arms B/C/D only)		X							
Urelumab (Arm C only)[8]		X							
Vaccine (Arms A/B/C only)			X						

In order to minimize the need for research-only in-person visits, telemedicine visits may be substituted for in-person clinical trial visits or portions of clinical trial visits where determined to be appropriate and where determined by the investigator not to increase the participants risks. Prior to initiating telemedicine for study visits the study team will explain to the participant, what a telemedicine visit entails, and confirm that the study participant is in agreement and able to proceed with this method. In the event telemedicine is not deemed feasible, the study visit will proceed as an in-person visit. Telemedicine visits will be conducted using HIPAA compliant method approved by the Health System and within licensing restrictions.

1. For women of childbearing potential. A urine test is required within a window of up to 4 days prior to nivolumab administration.
2. Pulse oximetry measured at Pre-Cycle #2 only. Blood pressure, pulse, and temperature will be measured on Day 0 any time prior to infusion, pre- and post-GVAX on Day 1, and any time prior to infusion on Day 14 (Arm D only).
3. Heme-8 with differential including absolute eosinophil count, absolute neutrophils, absolute lymphocytes
4. Comprehensive chemistry panel including electrolytes, BUN, creatinine, AST, ALT, total bilirubin, alkaline phosphatase
5. Total T3 and free T4 if TSH abnormal
6. Research samples will be collected at the discretion of the PI based on availability of supplies and safety of patient and staff.
7. CT of chest/abd/pelvis with iv contrast as per Institutional standard, e.g. pancreatic protocol at Johns Hopkins
8. No Urelumab will be administered after 9/30/2020. Patients remaining on Arm C after this date will receive the same intervention as Arm B patients.
9. Day 8 safety labs (Arms A/B/C only) may be done within a -1/+3 day window.
10. Day 14 labs and toxicity evaluation (Arm D only) may be done on Day 14 -1/+3 days.
11. Day 28 labs and toxicity evaluation (Arms A-C only) may be done on day 28 +/-3 days.

Table 2C Study Schedule of Immunotherapy Cycles #3 - #6

	Pre-Cycle #3	Pre-Cycle #4-6	Each cycle							EOT[12]
	Day -14 to Day 0	Day -7 to Day 0	0 +/-3	1	2-7	8	9-13	14 +/-2	15-28	
**Eligibility assessment**										
Inclusion/Exclusion[1]	X									
Medical history	X									
Pregnancy test	X[2]	X[2]								
Performance status	X	X								X
**Safety assessment**										
Vital signs[3]	X	X	X	X				X		X
Physical exam, weight	X	X								X
Toxicity assessments/ adverse events		X		X						X
**Laboratory test**										
Hematology[4]	X	X				X[10]		X[11]		X
Comprehensive[5]	X	X				X[10]		X[11]		X
CA 19-9	X									X
Amylase/Lipase	X	X				X[10]		X[11]		X
TSH[6]	X	X								X
Blood draw for PBMC, CTC, plasma, and serum (up to 145 cc)[7]	X									
**Efficacy Assessments**										
CT chest/abd/pelvis[8]	X									X
**Treatment**										
IV cyclophosphamide (Arms A/B/C only)			X							
BMS-986253 (Arm D only)			X					X		
Nivolumab (Arm B/C/D only)			X							
Urelumab (Arm C only)[9]			X							
Vaccine (Arms A/B/C only)				X						

In order to minimize the need for research-only in-person visits, telemedicine visits may be substituted for in-person clinical trial visits or portions of clinical trial visits where determined to be appropriate and where determined by the investigator not to increase the participants risks. Prior to initiating telemedicine for study visits the study team will explain to the participant, what a telemedicine visit entails, and confirm that the study participant is in agreement and able to proceed with this method. In the event telemedicine is not deemed feasible, the study visit will proceed as an in-person visit. Telemedicine visits will be conducted using HIPAA compliant method approved by the Health System and within licensing restrictions.

1. Review eligible criteria for continuation.
2. For women of childbearing potential. A urine test is required within a window of up to 4 days prior to nivolumab administration.
3. Pulse oximetry measured at Pre-Cycle #3 only. Blood pressure, pulse, and temperature will be measured on Day 0 any time prior to infusion, pre- and post-GVAX on Day 1, and any time prior to infusion on Day 14 (Arm D only).
4. Heme-8 with differential including absolute eosinophil count, absolute neutrophils, absolute lymphocytes
5. Comprehensive chemistry panel including electrolytes, BUN, creatinine, AST, ALT, total bilirubin, alkaline phosphatase.

6 Total T3 and free T4 if TSH abnormal

7. Research samples will be collected at the discretion of the PI based on availability of supplies and safety of patient and staff.
8. CT of chest/abd/pelvis with iv contrast as per Institutional standard, e.g. pancreatic protocol at Johns Hopkins
9. No Urelumab will be administered after 9/30/2020. Patients remaining on Arm C after this date will receive the same intervention as Arm B patients
10. Day 8 safety labs (Arms A/B C only) are required for cycle #3 only and may be done within a -1/+3 day window.
11. Day 14 labs (Arm D only) may be done up to 4 days prior to dosing. Amylase/lipase are only required for cycle 3.
12. End of Treatment (EOT) visit will be 28 days (+/-7) from the last dose of study drug. Patients in Arm A will be followed for toxicities through 28 days after the last immunotherapy and patients in Arm B and Arm C will be followed for toxicities through 100 days after the last immunotherapy

Table 2D Study Schedules of Immunotherapy Cycles #7 - #8 (Extended Treatment Phase)

	Each Extended Treatment Cycle (Cycles 7 – 8)								EOT[8]
	Day -7 to Day 0	Arms B/C/D only[7]				All Arms			
		0 +/-7	1-27	28 +/-3	29-55	56 +/-3	57	58-83	
**Eligibility assessment**									
Pregnancy test[1]		X		X		X			
Performance status	X			X[9]		X[9]			X
**Safety assessment**									
Vital signs[2]	X	X		X		X	X		X
Physical exam, weight	X			X[9]		X[9]			X
Toxicity assessments/ adverse events	X			X[9]		X[9]	X		X
**Laboratory test**									
Hematology[3]	X			X[9]		X[9]			X
Comprehensive[4]	X			X[9]		X[9]			X
CA 19-9	X								X
Amylase/Lipase	X			X[9]		X[9]			X
TSH[5]	X			X[9]		X[9]			X
**Efficacy Assessments**									
CT chest/abd/pelvis[6]	X								X
**Treatment**									
IV cyclophosphamide (Arms A/B/C only)						X			
Nivolumab (Arms B/C/D only)		X		X		X			
Vaccine (Arm A/B/C only)							X		

In order to minimize the need for research-only in-person visits, telemedicine visits may be substituted for in-person clinical trial visits or portions of clinical trial visits where determined to be appropriate and where determined by the investigator not to increase the participants risks. Prior to initiating telemedicine for study visits the study team will explain to the participant, what a telemedicine visit entails, and confirm that the study participant is in agreement and able to proceed with this method. In the event telemedicine is not deemed feasible, the study visit will proceed as an in-person visit. Telemedicine visits will be conducted using HIPAA compliant method approved by the Health System and within licensing restrictions.

1. For women of childbearing potential. A urine test is required within a window of up to 4 days prior to nivolumab administration.
2. Blood pressure, pulse, and temperature will be measured on Day 0 any time prior to infusion and pre- and post-GVAX on Day 1
3. Heme-8 with differential including absolute eosinophil count, absolute neutrophils, absolute lymphocytes
4. Comprehensive chemistry panel including electrolytes, BUN, creatinine, AST, ALT, total bilirubin, alkaline phosphatase.
5. Total T3 and free T4 if TSH abnormal
6. CT of chest/abd/pelvis with iv contrast as per Institutional standard, e.g. pancreatic protocol at Johns Hopkins
7. Evaluations do not need to be performed for patients on Arm A, who do not receive treatment until approximately Day 56
8. End of Treatment (EOT) visit will be 28 days (+/-7) from the last dose of study drug. Patients in Arm A will be followed for toxicities through 28 days after the last immunotherapy and patients in Arm B and Arm C will be followed for toxicities through 100 days after the last immunotherapy
9. Can be done within a window of up to 7 days prior to study treatment

3.3 Study population

3.3.1 Eligibility criteria

Eligibility to receive a study drug must be determined prior to the first, second and third cycles by the Principal Investigators or their designee prior to the administration of the research product. If, after the participant has met the eligibility criteria, the participant is reevaluated for other indications either clinically or by laboratory tests, such re-evaluations will not be considered as the re-valuation of eligibility. However, whether or not to proceed with the study treatment is at the discretion of principal investigator or the designee. Decisions still can be made to take the participant off the study based on such re-evaluations. If the eligibility criteria for vaccination are not met the research participant may be re-evaluated if the Principal Investigators anticipates that the research participant may later meet the eligibility criteria. There is no time limit.

3.3.1.1 Staging information

Staging criteria are from the "American Joint Committee on Cancer (AJCC) Staging Criteria for Pancreatic Cancer." Patients with stage ≤ IIb are eligible for this study.

Stage Grouping
Stage Ia T1 N0 M0
Ib T2 N0 M0
Stage IIa T3 N0 M0
Stage IIb T1-3 N1 M0
Stage III T4 Any N M0
Stage IV Any T Any N M1

Primary Tumor (T)
TX Primary tumor cannot be assessed
T0 No evidence of primary tumor
T1 Tumor limited to the pancreas 2 cm or less in greatest dimension
T2 Tumor limited to the pancreas more than 2 cm in greatest dimension
T3 Tumor extends beyond the pancreas but without involvement of the celiac axis or the superior mesenteric artery.
T4 Tumor involves the celiac axis or the superior mesenteric artery (unresectable primary tumor).

Regional Lymph Nodes (N)
NX Regional lymph nodes cannot be assessed
N0 No regional lymph node metastasis
N1 Regional lymph node metastasis

Distant Metastasis (M)
MX Presence of distant metastasis cannot be assessed
M0 No distant metastasis
M1 Distant metastasis

3.3.1.2 Criteria for resectability

Resectability is determined by the study team based on spiral CT with intravenous contrast enhancement or MRI if the subject is allergic to contrast, according to the AHPBA/SSO criteria and the NCCN criteria.

Patients who would be considered borderline resectable per the study team according to the AHPBA/SSO criteria and the NCCN criteria include patients with severe unilateral SMV/portal impingement, tumor abutment on the SMA, GDA encasement up to the origin at the hepatic artery, or colon invasion are not eligible for this study.

The surgery includes both open and laparoscopy Whipple procedure.

3.3.2 Eligibility Criteria for Preoperative Vaccination

Eligibility must be determined at the study entry by the Principal Investigators or their designee. **The patient must have biopsy-proven adenocarcinoma before randomization and starting the study treatment. However, if a biopsy is performed and it is not sufficient for a diagnosis, the patient can be considered to meet eligibility if clinically the tumor is suspected to be adenocarcinoma.** Pretreatment biopsy is necessary for enrollment in this study. If the patient is referred to this clinical trial with a diagnostic core biopsy, a research core biopsy will be performed unless a core biopsy specimen is available for the research purpose through archive. If the patient does not have a diagnostic biopsy, the research biopsy will be done at the same time as the diagnostic biopsy. There is a secondary and third evaluation for study continuation prior to the second and third vaccination cycle, respectively. After the patents are deemed eligible, the results of any evaluation should not be considered for re-evaluation of eligibility. However, such results may be considered as reasons for off-study; and whether or not to proceed with the study treatment according to these results will be determined at the discretion of the clinical judgment of the PI or designee. If the eligibility criteria for vaccination are not met the research participant may be re-evaluated if the Principal Investigators anticipates that the research participant may later meet the eligibility criteria. There is no time limit.

3.3.2.1 Inclusion Criteria:

Research participants must meet the following inclusion criteria for study entry:

1. Age $\geq$ 18 years.
2. Have a newly diagnosed, biopsy-proven adenocarcinoma of the head, neck and uncinate of the pancreas, and is a candidate for a pancreaticoduodenectomy. If the biopsy is not sufficient for diagnosis, the patient can be considered to meet eligibility if the study team agrees that clinically the patient’s tumor is suspected to be adenocarcinoma.
3. Patient’s tumor must be deemed resectable by the study team prior to registration. Borderline resectable patients will be excluded.
4. Ability to understand and willingness to sign a written informed consent document.

5. Agree to undergo a core biopsy of the pancreatic tumor for both research and diagnosis purposes if a prior core biopsy is not performed or the core biopsy specimen is not available for the research purpose of this study.

6. Have an Eastern Cooperative Oncology Group (ECOG) performance status of 0 or 1 **(Appendix A)**.

7. Have adequate organ and marrow function as defined below:

   - WBC $\geq$ 2000/μL
   - Neutrophils $\geq$ 1500/μL
   - ALC $\geq$ 500/uL
   - Platelets $\geq 100 \times 10^3$/μL
   - Hemoglobin $\geq$ 9.0 g/dL
   - Serum creatinine $\leq$ 1.5 x ULN or creatinine clearance (CrCl) $\geq$ 40 mL/min (if using the Cockcroft-Gault formula below):

     $$\textit{Female}\ \text{CrCl} = \frac{(140 - \text{age in years}) \times \text{weight in kg} \times 0.85}{72 \times \text{serum creatinine in mg/dL}}$$

     $$\textit{Male}\ \text{CrCl} = \frac{(140 - \text{age in years}) \times \text{weight in kg} \times 1.00}{72 \times \text{serum creatinine in mg/dL}}$$

   - AST and ALT $\leq$ 3.5 x ULN
   - Amylase $\leq$ 2 X ULN

8. Women of childbearing potential (WOCBP) must have a negative serum pregnancy test (minimum sensitivity 25 IU/L or equivalent units of human chorionic gonadotropin [HCG]). WOCBP is defined in **Section 3.10.4.**
   - WOCBP must agree to follow instructions for highly effective method(s) of contraception from the time of enrollment, through the duration of treatment with study drug(s) and through 26 weeks post treatment completion.
   - Men who are sexually active with WOCBP must agree to follow instructions for method(s) of contraception through the duration of treatment with study drug(s) through 31 weeks post-treatment completion.
   - Women who are not of childbearing potential (i.e., who are postmenopausal or surgically sterile) as well as azoospermic men do not require contraception.
   - Women must not be breastfeeding.
   - At least one barrier method of contraception must be employed by all sexually active patients (male and female), regardless of other methods, to prevent the transfer of body fluids.

3.3.2.2 Exclusion criteria:

Research participants with any of the following will be excluded from study entry:

1. History of any autoimmune disease, including but not limited to: Patients with a history of inflammatory bowel disease, including ulcerative colitis and Crohn's Disease, patients with a history of symptomatic disease (e.g., rheumatoid arthritis, systemic progressive sclerosis [scleroderma], systemic lupus erythematosus, autoimmune vasculitis [e.g., Wegener's Granulomatosis]); CNS or motor neuropathy considered of autoimmune origin (e.g., Guillain-Barre Syndrome and Myasthenia Gravis, multiple sclerosis). Patients with Graves or Hashimoto's disease, vitiligo, and type I diabetes mellitus will be allowed.

2. Systemic steroid therapy or immunosuppressive therapy within 14 days before vaccine administration.

3. Major active medical or psychosocial problems that could be exacerbated by the study treatment.

4. Evidence of active infections.

5. Have received any type of cancer immunotherapy including the same pancreatic cancer vaccine.

6. Have received any anti-pancreatic cancer therapy (symptomatic therapies are allowed)

7. Pregnant or breastfeeding.

8. Have been diagnosed with another cancer or myeloproliferative disorder whose natural history or treatment has the potential to interfere with safety or efficacy assessment of this study's investigational drugs.

9. History of severe hypersensitivity reaction to any monoclonal antibody.

10. Patient has a known or suspected hypersensitivity to GM-CSF, hetastarch, corn, dimethyl sulfoxide, fetal bovine serum, trypsin (porcine origin), yeast or any other component of GVAX pancreas vaccine

11. Have received a diagnosis of human immunodeficiency virus (HIV), hepatitis B or hepatitis C (patients who are hepatitis C antibody positive may be enrolled if they are confirmed with negative viral load at screening).

12. Patient has a pulse oximetry of <92% on room air.

13. Patient is on supplemental home oxygen.

Note that hyperbilirubinemia caused by tumor-associated biliary obstruction is reversible and is not associated with hepatic insufficiency. Therefore, we do not include the level of bilirubin as an eligibility criterion similar to the prior J0810 neoadjuvant immunotherapy study[25].

3.3.3 Additional Criteria for Study Continuation

3.3.3.1 Inclusion Criteria for Study Continuation

Research participants must meet the following criteria for post-operative vaccination and for post-chemoradiation vaccination:

1. Have a surgically resected (R0 or R1) AJCC pathologic stage I or stage II adenocarcinoma of the head, neck, or uncinate of the pancreas. (Following study treatment #1, if the patient's tumor is found intraoperatively to be limited to the distal portion (body or tail) of the pancreas and is resected by distal pancreatectomy, the patient may continue to receive study treatments but will be considered non-evaluable for the primary and efficacy endpoints and will be followed for additional endpoints.) The patients with an R2 resection will not be eligible for the continuation of the study. Patients with intraoperative findings of metastatic disease will not be eligible for the continuation of the study.

2. ECOG performance 0-1

3. Adequate marrow reserve with ANC >/= 1500/mm^3, platelet count >/= 100,000/mm^3, and hemoglobin >/= 9 gm/dl

4. Adequate hepatic function with a total Bilirubin ≤ 1.5 x ULN (except subjects with Gilbert Syndrome, who can have total bilirubin ≤ 2.0 × ULN), AST and ALT ≤ 2X upper level of normal, alk phos ≤ 5X upper level of normal

5. Adequate renal function with serum creatinine ≤ 2 mg/dl

6. Have an absolute lymphocyte count ≥ 500/mm^3

7. Women of childbearing potential (WOCBP) must have a negative pregnancy test (minimum sensitivity 25 IU/L or equivalent units of human chorionic gonadotropin [HCG]). WOCBP is defined in **Section 3.10.4.**

   - WOCBP must agree to follow instructions for method(s) of contraception from the time of enrollment, through the duration of treatment with study drugs, and through 26 weeks post treatment completion.
   - Men who are sexually active with WOCBP must agree to follow instructions for method(s) of contraception for the duration of treatment with study drugs, through 31 weeks post-treatment completion.
   - At least one barrier method of contraception must be employed by all sexually active patients (male and female), regardless of other methods, to prevent the transfer of body fluids.

3.3.3.2 Exclusion Criteria for Study Continuation

Research participants with any of the following will be excluded from study continuation:

1. Radiographic evidence of pancreatic cancer recurrence.
2. Not able to receive the vaccination within 10 weeks following the surgery secondary to a delayed recovery from the surgery
3. Major active medical or psychosocial problems that could be exacerbated by this treatment.
4. History of any autoimmune disease, including but not limited to: Patients with a history of inflammatory bowel disease, including ulcerative colitis and Crohn's Disease, patients with a history of symptomatic disease (e.g., rheumatoid arthritis, systemic progressive sclerosis [scleroderma], systemic lupus erythematosus, autoimmune vasculitis [e.g., Wegener's Granulomatosis]); CNS or motor neuropathy considered of autoimmune origin (e.g., Guillain-Barre Syndrome and Myasthenia Gravis, multiple sclerosis). Patients with Graves or Hashimoto's disease, vitiligo, and type I diabetes mellitus will be allowed.
5. Systemic steroid therapy or immunosuppressive therapy within 14 days before vaccine administration.
6. Evidence of active infections.
7. Pregnant or breastfeeding.
8. Have been diagnosed with another cancer or myeloproliferative disorder whose natural history or treatment has the potential to interfere with the safety or efficacy assessment of this study's investigational drugs.
9. History of severe hypersensitivity reaction to any monoclonal antibody.
10. Patient has a known or suspected hypersensitivity to GM-CSF, hetastarch, corn, dimethyl sulfoxide, fetal bovine serum, trypsin (porcine origin), yeast or any other component of GVAX pancreas vaccine
11. Have received a diagnosis of human immunodeficiency virus (HIV), hepatitis B or hepatitis C (patients who are hepatitis C antibody positive may be enrolled if they are confirmed with negative viral load at screening).
12. Patient has a pulse oximetry of <92% on room air.
13. Patient is on supplemental home oxygen.

3.4 Production and Administration of Study Drugs

3.4.1 Vaccine

[redacted]

[redacted]

[redacted]

[redacted]

Special considerations for vaccination

Systemic steroid therapy or immunosuppressive therapy cannot be given within 14 days before each vaccine administration. However, study-related treatments may be given after short-term steroid use (⩽ 4 days) with prior approval by the protocol chair and IND sponsor.

Management of toxicities of vaccination

Local vaccine site reaction may be treated with topical applications of aloe vera or vitamin E gel or lotion. Significant local inflammation that is causing the research participant severe pain or is interfering with the activities of daily living may be treated with cold packs and oral analgesics. Local toxicities of pruritus at the vaccine sites and systemic pruritus may be treated with topical or oral diphenhydramine hydrochloride (Benadryl) or topical aloe vera. If oral diphenhydramine hydrochloride is used the recommended dose shall be 25-50 mg every four to six hours as needed for pruritus, not to exceed 300 mg/day. Cases of local ulceration should be manageable with local wound care, with or without antibiotics. Severe local inflammation or significant clinical autoimmunity will be managed on a case by case basis.

3.4.2 Nivolumab

3.4.2.1 Preparation

[redacted]

[redacted]

[redacted]

3.4.2.2 Storage of Infusion Solution

[redacted]

3.4.2.3 Administration

[redacted]

DOSAGE FORMS AND STRENGTHS

[redacted]

3.4.3 Urelumab

3.4.3.1 Preparation

[redacted]

[redacted]

3.4.3.2 Storage of Infusion Solution

[redacted]

3.4.3.3 Administration

[redacted]

DOSAGE FORMS AND STRENGTHS

[redacted]

3.4.4 BMS-986253 (anti-IL8 antibody)

3.4.4.1 Description and Preparation

[redacted]

[redacted]

[redacted]

3.4.4.2 Storage of Infusion Solution

[redacted]

3.4.4.3 Administration

[redacted]

[redacted]

DOSAGE FORMS AND STRENGTHS

[redacted]

3.4.5 Cyclophosphamide

Cyclophosphamide will be administrated at 200 mg/m^2 intravenously over 30 minutes +/- 10 minutes. For subjects on Arm B, cyclophosphamide should be administrated first and patients should be observed for 30 minutes before administration of nivolumab.

Management of the toxicity of low dose cyclophosphamide

For patients with grades 3 and 4 leukopenia, neutropenia, or thrombocytopenia who are due to receive IV cyclophosphamide, both the IV cyclophosphamide and vaccine will be held until the patient's counts return to grade 2 or above. The patients will be taken off the study if their counts do not return to grade 2 or above within 6 months during vaccination cycles following adjuvant chemoradiation or as specified elsewhere (section 3.5.4) in the protocol prior to chemoradiation. Prophylactic doses of trimethoprim-sulfamethoxazole (Bactrim) will be recommended to all patients with sustained grade 3 or 4 lymphopenia or neutropenia lasting more than a month at the discretion of the treating physician. If the patient is allergic to trimethoprim-sulfamethoxazole, dapsone will be recommended as an alternative.

Patients receiving cyclophosphamide who develop hematuria or polyuria will undergo urinalysis and cytology. If hemorrhagic cystitis is identified, oral or IV cyclophosphamide will be discontinued and appropriate treatments will be initiated as per standard of care. Such patients may receive their scheduled vaccination at the discretion of the study team and appropriate surveillance will be performed for transitional cell carcinoma of the bladder.

3.4.6 Management of Infusion Reactions

Nivolumab infusion related reactions are common and are described in the investigator's brochure for nivolumab. Infusion reactions to study drug may manifest with fever, chills, rigors, headache, rash, pruritis, arthralgias, hypo- or hypertension, bronchospasm, or other symptoms.

All Grade 3 or 4 infusion reactions should be reported as an SAE if criteria are met. Infusion reactions should be graded according to NCI CTCAE 5.0 guidelines.

The following AE terms, regardless of grade, are considered ECIs and should be reported to the Sponsor within 24 hours of the event

– Allergic reaction

– Anaphylaxis

– Cytokine release syndrome

– Serum sickness

– Infusion reactions

– Infusion-like reactions

Please note, the AE should be reported regardless of etiology.

Treatment of Study Drug Related Infusion Reactions

Treatment recommendations are provided below and may be modified based on local treatment standards and guidelines as appropriate:

For Grade 1 symptoms: (Mild reaction; infusion interruption not indicated; intervention not indicated)

Remain at bedside and monitor subject until recovery from symptoms. The following prophylactic premedications are recommended for future infusions: diphenhydramine 50 mg (or equivalent) and/or paracetamol 325 to 1000 mg (acetaminophen) at least 30 minutes before additional nivolumab administrations.

For Grade 2 symptoms: (Moderate reaction requires therapy or infusion interruption but responds promptly to symptomatic treatment [e.g., antihistamines, non-steroidal anti-inflammatory drugs, narcotics, corticosteroids, bronchodilators, IV fluids]; prophylactic medications indicated for 24 hours).

Stop the study drug infusion, begin an IV infusion of normal saline, and treat the subject with diphenhydramine 50 mg IV (or equivalent) and/or paracetamol 325 to 1000 mg (acetaminophen); remain at bedside and monitor subject until resolution of symptoms. Corticosteroid or bronchodilator therapy may also be administered as appropriate. If the infusion is interrupted, then

restart the infusion at 50% of the original infusion rate when symptoms resolve; if no further complications ensue after 30 minutes, the rate may be increased to 100% of the original infusion rate. Monitor subject closely. If symptoms recur then no further nivolumab will be administered at that visit. Administer diphenhydramine 50 mg IV, and remain at bedside and monitor the subject until resolution of symptoms. The amount of study drug infused must be recorded on the electronic case report form (eCRF). The following prophylactic premedications are recommended for future infusions: diphenhydramine 50 mg (or equivalent) and/or paracetamol 325 to 1000 mg (acetaminophen) should be administered at least 30 minutes before additional nivolumab administrations. If necessary, corticosteroids (recommended dose: up to 25 mg of IV hydrocortisone or equivalent) may be used.

For Grade 3 or Grade 4 symptoms: (Severe reaction, Grade 3: prolonged [i.e., not rapidly responsive to symptomatic medication and/or brief interruption of infusion]; recurrence of symptoms following initial improvement; hospitalization indicated for other clinical sequelae [e.g., renal impairment, pulmonary infiltrates]). Grade 4: (life threatening; pressor or ventilatory support indicated).

Immediately discontinue infusion of study drug. Begin an IV infusion of normal saline, and treat the subject as follows. Recommend bronchodilators, epinephrine 0.2 to 1 mg of a 1:1,000 solution for subcutaneous administration or 0.1 to 0.25 mg of a 1:10,000 solution injected slowly for IV administration, and/or diphenhydramine 50 mg IV with methylprednisolone 100 mg IV (or equivalent), as needed. Subject should be monitored until the investigator is comfortable that the symptoms will not recur. Study drug will be permanently discontinued. Investigators should follow their institutional guidelines for the treatment of anaphylaxis. Remain at bedside and monitor subject until recovery from symptoms. In the case of late-occurring hypersensitivity symptoms (e.g., appearance of a localized or generalized pruritis within 1 week after treatment), symptomatic treatment may be given (e.g., oral antihistamine, or corticosteroids).

3.4.7 Management of Study Drug-Related Adverse Events

3.4.7.1 Dosing delays

Dose reduction or dose increase of study drugs will not be permitted.

Systemically active steroids can be used but should be reported to the protocol chair and IND sponsor. Extended steroid treatment (> 4 days) must be completed at least 14 days prior to resuming study-related treatments. Study-related treatments may resume after short-term steroid use (≤ 4 days) with prior approval by the protocol chair and IND sponsor.

If the start of a cycle is delayed, all study drug administration will also be delayed accordingly until the subject meets the eligibility criteria for the study treatment..

If a delay occurs between Day 0 and 1 in a cycle:

- Infusion reactions must resolve to baseline prior to administration of GVAX.
- Resume Day 1 treatment schedule (GVAX) and assessments without repeating Day 0 study treatments (Cy and/or nivolumab and/or urelumab) if all Day 0 study treatments have been given and if the delay is within 72 hours.

- If only a portion of Day 0 treatments have been given, and the delay is ≤ 72 hours, only the remaining portion of Day 0 treatments will be given on the new Day 0, followed by GVAX on the new Day 1.
- If the delay is longer than 72 hours, repeat all Day 0 and Day 1 study treatments and assessments with a minimum of 1 week from the previous Day 0 treatment. This includes steroid treatment requiring at least a 14 day washout prior to resuming study-related treatments. Cy and GVAX will be also delayed until subjects may resume treatment with nivolumab and/or urelumab.

Day 0 study drug administration should be delayed for the following:

- Any grade ≥ 2 non-skin, treatment-related AE, with the following exceptions:
    - Grade 2 drug-related fatigue or laboratory abnormalities do not require a treatment delay
    - Grade 2 hypothyroidism or thyroiditis
- Any grade ≥3 skin treatment-related AE
- Any ≥ grade 3 treatment-related laboratory abnormality, with the following exceptions for asymptomatic amylase or lipase:
    - Grade 3 or 4 amylase or lipase abnormalities that are not associated with symptoms or clinical manifestations, or radiographic signs of pancreatitis do not require a dose delay. It is recommended to consult with the Principal Investigator for grade 3 amylase or lipase abnormalities.
    - Isolated grade 3 or 4 electrolyte imbalances/abnormalities that are not associated with clinical sequelae and are corrected with supplementation/appropriate management
- Any AE, laboratory abnormality, or intercurrent illness which, in the judgment of the investigator, warrants delaying the dose of study medication.

In order to standardize the management of AEs for all subjects, drug-related toxicity management will follow **Appendix B.**

Subjects may resume treatment with nivolumab and urelumab when the treatment-related AE(s) resolve to grade ≤1 or baseline value, with the following exceptions:

- Subjects may resume treatment in the presence of grade 2 fatigue.
- Subjects who have not experienced a Grade 3 drug-related skin AE may resume treatment in the presence of Grade 2 skin adverse event
- Subjects may resume treatment in the presence of grade 2 AST/ALT OR grade 1 total bilirubin. Subjects with combined grade 2 AST/ALT AND total bilirubin values meeting discontinuation parameters below (**Section 3.4.2.5.2**) should have treatment permanently discontinued.
- Treatment-related pulmonary toxicity, diarrhea, or colitis must have resolved to baseline before treatment is resumed.
- Treatment-related endocrinopathies adequately controlled with only physiologic hormone replacement may resume treatment, which include grade 2 hypothyroidism and thyroiditis.

Neutropenia management for the combination of urelumab and nivolumab:

[redacted]

3.4.7.2 Permanent discontinuation of study drug should be considered for any of the following unacceptable toxicities:

- Severe or life-threatening related AEs, including, but not limited to, any of the following (the IND Sponsor and BMS must be notified in the event of these AEs):
- Any grade 2 treatment-related uveitis, eye pain, or blurred vision that does not respond to topical therapy and does not improve to Grade 1 severity within 2 weeks of starting therapy
- Any grade 3 non-skin, drug-related AE lasting > 7 days, with the following exceptions:
    - Grade 3 treatment-related uveitis, pneumonitis, bronchospasm, diarrhea, colitis, neurologic toxicity, hypersensitivity reaction, or infusion reaction (applies to nivolumab and/or urelumab and/or BMS-986253 only) of any duration requires discontinuation
    - Grade 3 treatment-related laboratory abnormalities do not require treatment discontinuation except:
        - Grade 3 treatment-related thrombocytopenia that is associated with bleeding requires discontinuation
        - Any treatment-related liver function test (LFT) abnormality that meets the following criteria require discontinuation:
            - AST or ALT > 8 x ULN
            - Total bilirubin > 5 × ULN
            - Concurrent AST or ALT > 3 × ULN **and** total bilirubin > 2 × ULN
- Any grade 4 treatment-related AE or laboratory abnormality, except for the following events which do not require discontinuation:
    - Grade 4 amylase or lipase abnormalities that are not associated with symptoms or clinical manifestations, or radiographic signs of pancreatitis. It is recommended to consult with the Principal Investigator for grade 4 amylase or lipase abnormalities.
    - Isolated grade 4 electrolyte imbalances/abnormalities that are not associated with clinical sequelae and are corrected with supplementation/appropriate management.
    - Grade 4 lymphopenia.

- Grade 4 neutropenia that resolves to grade 1 within 7 days of administration of G-CSF

- Any dosing interruption lasting > 6 weeks with the following exceptions:
    - Dosing interruptions to allow for prolonged steroid tapers to manage drug-related adverse events are allowed. Prior to re-initiating treatment in a subject with a dosing interruption lasting > 6 weeks, the Principal Investigator must be consulted. Tumor assessments should continue as per protocol even if dosing is interrupted.
    - Dosing interruptions > 6 weeks that occur for non-drug-related reasons may be allowed if approved by the Principal Investigator. Prior to re-initiating treatment in a subject with a dosing interruption lasting > 6 weeks, the Principal Investigator must be consulted. Tumor assessments should continue as per protocol even if dosing is interrupted.

- Any AE, laboratory abnormality, or intercurrent illness which, in the judgment of the Investigator, presents a substantial clinical risk to the subject with continued study drug dosing.

In order to standardize the management of AEs for all subjects, and since it would be impossible to distinguish between AEs related to each of the various investigational study drugs, we will use the treatment management algorithms for nivolumab included in **Appendix B**. Additional AE treatment management algorithms included in the nivolumab Investigator's Brochure (IB) might be considered for individual cases.

Subjects on Arms containing GVAX that are required to permanently stop treatment with the Day 0 study drugs due to toxicity may stay on study and receive CY/GVAX pancreas vaccine once the nivolumab-related toxicity(s) has resolved to a grade 1.

3.5 End of Treatment visit

All patients will have an End of Treatment visit 28 days (+/- 7 days) after their last dose of immunotherapy on study. This may be after completing all 8 cycles or earlier if the patient is taken off study early for any reason. Patients in Arm A will be followed for toxicities through 28 days after the last immunotherapy; all other patients will be followed for toxicities through 100 days after the last immunotherapy.

The following evaluations will be performed at the off study visit:

1. History and Physical exam with ECOG performance
2. Assessment of toxicities (may include evaluations made by local healthcare provider)
3. Assessment of vaccine sites. This will include: number of sites that have erythema, induration, pruritus, and tenderness; and measurement of induration and erythema of largest vaccine site.

4. Heme-8 with differential, including absolute eosinophil count, absolute neutrophils, absolute lymphocytes

5. Comprehensive panel including electrolytes, BUN, creatinine, AST, ALT, alkaline phosphatase, total bilirubin, lipase, amylase, and TSH (and total T3 and free T4 if TSH abnormal).

6. CA19-9

7. CT scan abdomen/pelvis and chest. If done within 30 days the CT scans do not need to be repeated. (If allergic to CT scan contrast, obtain MRI).

3.6 Follow-up phase

Patients are considered to enter the follow-up phase at the End of Treatment visit (28 days +/-7 days after their last dose of immunotherapy on study. The subjects will be followed for overall survival every 3 months (+/- 2 weeks) for the first 24 months and then every 6 months (+/- 4 weeks) for another 12 months. Survival follow-up will then continue every 6 months (+/- 4 weeks) until 1) death, 2) withdrawal of consent to follow-up or 3) study closure.

All subjects who discontinued study treatment without disease progression should continue to be monitored for disease status by radiologic imaging. Imaging may be done by the patient's local provider and requested by the study team. Timing of these follow-up scans should ideally match the timing of survival follow-up assessments noted above, though they will not be considered deviations if local providers order scans more or less frequently. Disease monitoring should continue to be assessed as noted above until, 1) start of a new antineoplastic therapy (information of the new cancer therapy will be collected), 2) disease progression, 3) death, 4) withdrawal of consent to follow-up, or 5) study closure, whichever occurs first.

Information regarding other cancer therapies after end of study treatment may also be collected. Follow-up assessments may be made by phone, email, visit, or record collection.

Subjects who are discontinued from the study treatment due to an unacceptable drug-related AE will also be monitored for safety until the resolution of the AE to ≤ grade 1 or stabilization or until initiation of a new therapy for their cancer, whichever occurs first.

All subjects will be followed for at least 4 weeks after their last dose of study drug for the development of AEs. SAEs that occur within 100 days of the last nivolumab, urelumab, or BMS-986253 for patients in Arms B/C/D, and within 28 days of the Cy/GVAX treatment for patients in Arm A, and before initiation of a new antineoplastic treatment (whichever comes first) should also be followed and recorded.

3.7 Treatment Discontinuation and Off study

The patient may be discontinued from treatment in the following instances:

1. Patient withdraws consent and refuses future treatment (note: unless patient specifically withdrawals consent to future follow-up, they will be considered off treatment, but in follow-up)

2. Patient is noncompliant with study treatment

3. Patient is lost to follow-up

4. Concurrent illness develops that would preclude objective clinical assessments

5. Patient becomes pregnant

6. Disease progression

7. The incidence or severity of adverse events in this study denotes potential untoward health risk to the patient.

8. Patient receives non-study immunotherapy, chemotherapy, radiotherapy, gene therapy, biologic therapy, or other investigational therapy for the treatment of pancreatic cancer.

9. Patient experiences an unacceptable toxicity attributed to the study drug(s). Unacceptable toxicities are defined in section 3.4.7.2

Patients that are discontinued from treatment should have an End Of Treatment visit (see Section 3.5) and are will be considered in follow-up (see Section 3.6) until 1) death, 2) study closure, 3) withdrawal of consent from future follow-up, or 4) if they have completed the 3 year follow-up, at which point patients will be considered off study.

At the conclusion of the study, all remaining subjects that received at least 1 dose of GVAX will be offered enrollment in a long-term follow-up study and continue to be evaluated for survival and clinical and immunological responses. Per the FDA requirement for patients treated with genetically modified products, all research participants will be encouraged to enroll in a long-term follow-up protocol, following their completion of all interventional studies. These patients will be followed for disease progression, survival and potential long term toxicity of gene therapy in an existing protocol entitled "Long term follow-up of patients who received lethally irradiated allogeneic pancreatic tumor cells transfected with the GM-CSF gene (IRB # 02-10-14-03, SKCCC J0248)".

3.8 Collection samples for correlative studies

3.8.1 Leukapheresis or Blood Collection

All research participants will undergo a standard leukapheresis or approximately 145 cc of blood drawn before and after the first two vaccinations, and before the third vaccination. Blood may be used for isolation of PBMC (peripheral blood mononuclear cells), plasma, or serum.

Any research participant demonstrating an interesting immunological response may be asked to undergo additional leukapheresis or blood draw for research purposes. This may include physical responses thought to be related to the vaccine (including, but not limited to vaccine site flares) or interesting laboratory responses (including, but not limited to mesothelin-specific CD8+T cell responses). There will be at least one month between additional leukapheresis procedures. Prior to the leukapheresis, subjects will be evaluated by the Hematopoietic and Therapeutic Support (HATS) Center to determine if their vascular access appears to be adequate for the leukapheresis procedure. The quantity of these samples is necessary for monitoring the quantitative change of peripheral lymphocytes including PD-1+ cells and functional analysis of T cell immune response

following each vaccination. Detailed instructions for blood collections, processing and storage are provided in the Lab Manual.

3.8.2 Tumor tissue banking

A portion of the resected tumor will be collected for tumor banking. If the participant has undergone a core biopsy of tumor at the time of initial diagnosis, we will collect a portion of available biopsy sample for tumor banking; otherwise, research biopsy specimens will also be collected for tumor bank. The tumor samples will be processed and stored as per standard for the subsequent analyses including those of tumor antigens and infiltrating immune cells. Methods of analyses include immunohistochemistry and quantitative real-time PCR, etc. Frozen tumor samples may also be used to generate autologous tumor cell lines and xenograft tumor models. Tumor tissue specimens will be used for cancer biology and immunology studies.

Results from the sequencing studies will not be released to the patients. These studies are for research purposes only and are not using a clinically validated platform.

3.9 Evaluation for safety and anticipated toxicities

3.9.1 Safety and anticipated toxicities of vaccine

Severe toxicities are unlikely, based on information from previous GM-CSF gene vaccine studies completed here at Johns Hopkins Medicine. This includes the Phase I and II pancreatic tumor vaccine trials. In experiments involving over 400 mice, use of irradiated GM-CSF secreting tumor cells caused only reversible lymphadenopathy and reversible subcutaneous swelling; no ulcerations were seen. In our first phase I trial in patients with renal cell carcinoma, only local erythema and swelling were seen following intradermal injections of cell doses up to 4×10^7 GM-CSF modified vaccine cells, and up to 4×10^8 unmodified vaccine cells. At the highest dose level, we predict that initially 45 mcg of total GM-CSF will be secreted locally per 24 hours, a level that will diminish as tumor cells are killed by invading inflammatory cells. To support hematologic recovery in oncology patients after intensive chemotherapy, subcutaneous or intravenous doses of GM-CSF between 5 and 10 μg/kg/day are commonly used (350-700 mcg total for a 70 kg individual). At this dose range the following side effects are commonly seen: local or generalized skin rashes, bone pain (attributed to stimulation of hematopoietic progenitors), fever, and malaise. Although patients in the initial Phase I study of the allogeneic tumor vaccine had normal bone marrow function, leukocytosis and toxic levels of serum GM-CSF did not occur with the 10 fold lower dose of GM-CSF. The maximum serum GM-CSF level obtained was 14.0 pg/ml with dose level four at 48 hours after the first pancreatic tumor vaccine. The plasmid used to transfect the GM-CSF gene is safe. In contrast to retroviral vectors, it lacks the coding sequences that would allow replication and the generation of helper virus. This plasmid containing the GM-CSF gene has been sequenced following vector construction to confirm its insertion, orientation, and the lack of mutations. In addition, this vector has been confirmed to produce GM-CSF.

The risk of generating autoimmune reactions is unknown but is believed to be small. The pancreas would be the most likely organ to be involved. Pancreatitis and loss of pancreatic function can be supported by the use of exogenous pancreatic enzymes and insulin injections if needed. Other organs that may share tissue specific antigens might also be involved, such as the salivary glands

and other gastrointestinal organs. In the Phase I and II studies there were no evidence of autoimmune reactions. Every patient who has received the vaccine will be evaluated for toxicity. The research participant will be taken off-study if unacceptable adverse events are experienced. Possible toxicities include local swelling, induration, or ulceration at the site of the vaccine, systemic toxicities from paracrine secretion of GM-CSF, and induction of autoimmunity. The risk of generating autoimmune reactions might be increased by combination of nivolumab. The Phase I study of nivolumab suggest that the incidence of nivolumab-related autoimmune reaction is low. Therefore, we anticipate that such a risk is still small even if cyclophosphamide is combined with the vaccine.

3.9.2 Safety and anticipated toxicities of single injection of low dose cyclophosphamide

The dose of cyclophosphamide studied in this trial is below that in common use for the adjuvant therapy of e.g. breast cancer (typically 600 mg/m^2 of cyclophosphamide). Therefore, we anticipate that the risk of toxicity related to the use of single dose of cyclophosphamide at 200 mg/m^2 is quite small. Based on the toxicity studies of higher dose of cyclophosphamide in adjuvant therapy of breast cancer, fatigue, alopecia, nausea, vomiting, and mild cytopenias are likely to be the most common toxicities of cyclophosphamide. With standard dose cyclophosphamide, a rare incidence of leukemia or myelodysplastic syndrome as a late toxicity was reported.

The pharmacokinetics of cyclophosphamide is not significantly altered in the presence of hepatic insufficiency and/or hyperbilirubinemia. No dosage adjustments are currently recommended as a standard care. Therefore, it is anticipated to be safe to give cyclophosphamide in the setting of hyperbilirubinemia caused by extrahepatic biliary obstruction which is a common presentation of patients who has a newly diagnosed pancreatic cancer. In addition, hyperbilirubinemia caused by tumor-associated biliary obstruction is reversible and is not associated with hepatic insufficiency. Therefore, we do not include the level of bilirubin as an eligibility criteria.

Long-term daily use of cyclophosphamide may have different toxicity profiles from higher dose of cyclophosphamide given intravenous every 2-3 weeks. Nonetheless, animal studies still suggest that continuous administration of metronomic dose of cyclophosphamide is significantly less toxic than the maximum tolerated dose of cyclophosphamide given intermittently for 3 doses. The toxicities of metronomic cyclophosphamide in humans can be anticipated based on the previously published studies. Orlando et al. reported in patients with metastatic breast cancer that prolonged treatment of metronomic cyclophosphamide at 50 mg/day for a median duration of treatment of 20.4 months was well tolerated and side-effects were mild. The most frequently encountered toxicity was grade 1-2 leukopenia, which was observed in 54% of the 63 cases. Increases in transaminase values were registered in 12 cases, and one patient had grade 3 toxicity. Other side-effects included one patient with grade-3 thrombocytopenia, five with grade 1-2 anemia, ten with grade-1 and one with grade-2 nausea/vomiting, and small percentages of patients with grade 1-2 mucositis, gastric pain, diarrhea, fever, infection, asthenia, etc. It should be noted that patients in this study were also given metronomic dose of methotrexate which likely resulted in some of the side effect profiles[34]. Our J0810 study and other published studies reported similar toxicity profiles with prolonged use of immune modulatory doses of cyclophosphamide[15,23]. Long term use of metronomic cyclophosphamide has the potential to be associated with leucopenia, thrombocytopenia, and infection which may have an impact on surgical outcomes. However, none of these side-effects have been reported after short-term use of low dose cyclophosphamide[25]. The

single intravenous dose of cyclophosphamide has been given prior to vaccination in patients with advanced pancreatic cancer[15]. This did not reveal any of the previously mentioned side-effects. Taken together, an immune modulatory dose of cyclophosphamide given prior to the preoperative vaccination is not anticipated to cause side-effects that would potentially delay the surgery or affect outcomes of the surgery. Nonetheless, whether the combination of cyclophosphamide, nivolumab, and GVAX will have an impact on the surgery will be closely monitored as one of endpoints of this study.

3.9.3 Safety and anticipated toxicities of Nivolumab, Urelumab, and BMS-986253

Possible toxicities may be anticipated based on:

1. The observations in the phase I/II/III studies of nivolumab, urelumab, and BMS-986253.

2. General experience with other FDA-approved monoclonal antibodies (see Introduction and Rationale);

3. Observations in preclinical models of PD-1 blockade or deficiency, as well as preclinical assessment of nivolumab (see Introduction and Rationale);

4. Observations in clinical studies with other investigational agents that target the costimulatory T-cell pathway, such as ipilimumab (MDX-010), an antibody that blocks CTLA-4, and such as other anti-PD-1 or PD-L1 antibodies. Given the intended mechanism of action of nivolumab, particular attention will be given to events that may follow enhanced T-cell activation such as dermatitis, pneumonitis, and colitis. In addition to standard monitoring of clinical adverse events, hematology and chemistry parameters, additional oversight will follow the possible emergence of immune-mediated effects. Patients with a history of active autoimmune disease are excluded from this study, and results from a panel of autoimmune serological studies and a panel of pituitary function tests will be compared between baseline and the end of treatment. In addition, troponin and CPK levels will be followed. Symptom-directed workup of emergent adverse events will include an evaluation of autoimmune etiologies.

All the patients enrolled in this study will be evaluated for toxicity. Patients will be evaluated on day 1 by a research nurse following the study drug administration to monitor for local and systemic toxicities. The research participant may be contacted by phone or e-mail or the information may be obtained from their local health care providers. The research participant will be advised to call the research nurse and/or the principal investigator if there are any new toxicities, concerns or questions.

We anticipate that similar low toxicity profiles will be associated with the first vaccination given preoperatively. The anticipated toxicity associated with the vaccination is not anticipated to delay the surgery or affect the outcome of the surgery. The toxicity events associated with the first vaccination will be monitored.

3.9.4 WOCBP, Contraception, Use in Pregnancy, Use in Nursing

A WOCBP is defined as any female who has experienced menarche and who has not undergone surgical sterilization (hysterectomy or bilateral oophorectomy) and is not postmenopausal. Menopause is defined as 12 months of amenorrhea in a woman over age 45 years in the absence

of other biological or physiological causes. In addition, women under the age of 55 years must have a serum follicle stimulating hormone (FSH) level > 40mIU/mL to confirm menopause.*

*Women treated with hormone replacement therapy (HRT) are likely to have artificially suppressed FSH levels and may require a washout period in order to obtain a physiologic FSH level. The duration of the washout period is a function of the type of HRT used. The duration of the washout period below are suggested guidelines and the investigators should use their judgment in checking serum FSH levels. If the serum FSH level is >40 mIU/ml at any time during the washout period, the woman can be considered postmenopausal.

- 1 week minimum for vaginal hormonal products (rings, creams, gels)
- 4 week minimum for transdermal products
- 8 week minimum for oral products

3.9.4.1 Contraception

The investigational agents used in this protocol may have adverse effects on a fetus *in utero*. Furthermore, it is not known if the investigational agents have transient adverse effects on the composition of sperm. Non-pregnant, non-breast-feeding women may be enrolled if they are considered highly unlikely to conceive. Highly unlikely to conceive is defined as 1) surgically sterilized, or 2) postmenopausal (a woman who is ≥45 years of age and has not had menses for greater than 2 years will be considered postmenopausal), or 3) amenorrheaic for <2 years without a hysterectomy and oophorectomy and with a documented FSH value in the postmenopausal range, or 4) not heterosexually active for the duration of the study, or 5) heterosexually active and willing to use highly effective methods of birth control (which is also required for the female partners of male subjects). WOCBP enrolled in this study must agree to use an adequate method of contraception starting with Visit 1 through 26 weeks after the last dose of study drug. Male subjects enrolled in this study must also agree to use an adequate method of contraception starting with Visit 1 through 31 weeks after the last dose of study drug.

Investigators shall counsel WOCBP and male subjects who are sexually active with WOCBP on the importance of pregnancy prevention and the implications of an unexpected pregnancy Investigators shall advise WOCBP and male subjects who are sexually active with WOCBP on the use of highly effective methods of contraception. Highly effective methods of contraception have a failure rate of < 1% per year when used consistently and correctly.

HIGHLY EFFECTIVE METHODS OF CONTRACEPTION

- Hormonal methods of contraception including combined oral contraceptive pills, vaginal ring, injectables, implants, and intrauterine devices (IUDs) such as Mirena by WOCBP subject or male subject’s WOCBP partner.
- Nonhormonal IUDs, such as ParaGard
- Tubal ligation
- Vasectomy
- Complete abstinence*

*Complete abstinence is defined as complete avoidance of heterosexual intercourse and is an acceptable form of contraception for all study drugs. Abstinence is only acceptable when this is in line with the preferred and usual lifestyle of the subject. Subjects who choose complete abstinence are not required to use a second method of contraception, but female subjects must continue to have pregnancy tests. Acceptable alternate methods of highly effective contraception must be discussed in the event that the subject chooses to forego complete abstinence.

LESS EFFECTIVE METHODS OF CONTRACEPTION

- Diaphragm with spermicide
- Cervical cap with spermicide
- Vaginal sponge
- Male condom with or without spermicide*
- Progestin only pills by WOCBP subject or male subject's WOCBP partner
- Female condom*

*A male and female condom must not be used together

UNACCEPTABLE METHODS OF CONTRACEPTION

- Periodic abstinence (e.g., calendar, ovulation, symptothermal, post-ovulation methods)
- Withdrawal
- Spermicide only
- Lactation amenorrhea method

Subjects should be informed that taking the study medication may involve unknown risks to the fetus (unborn baby) if pregnancy were to occur during the study. In order to participate in the study they must adhere to the contraception requirement (described above) for the duration of the study. If there is any question that a subject will not reliably comply with the requirements for contraception, that subject should not be entered into the study.

3.9.4.2 Use in Pregnancy

The investigational agents used in this protocol may have adverse effects on a fetus; therefore, women with a positive pregnancy test at screening will not be eligible for enrollment. If a subject inadvertently becomes pregnant while on treatment, the subject will immediately be removed from the study. The site will contact the subject at least monthly and document the subject's status until the pregnancy has been completed or terminated.

The investigator must immediately notify the IND Sponsor and BMS of any pregnancy and complete and forward a Pregnancy Surveillance Form to the IND Sponsor within 24 hours and in accordance with the SAE reporting procedures described below. Any pregnancy that occurs in a female partner of a male study participant should be reported to the IND Sponsor and BMS.

Protocol required procedures for study discontinuation and follow-up must be performed on the subject unless contraindicated by pregnancy (e.g., x-ray studies). Other appropriate pregnancy follow-up procedures should be considered if indicated.

Follow-up information regarding the course of the pregnancy, including perinatal and neonatal outcome and where applicable, offspring information must be reported on the Pregnancy Surveillance Form.

3.9.4.3 Use in Nursing Women

Since many drugs are excreted in human milk, and because of the potential for serious adverse reactions in the nursing infant, subjects who are breast-feeding are not eligible for enrollment.

3.9.4.4 All Subjects (Male and Female)

All sexually active patients must use at least a barrier method (i.e., condom) to prevent transmission of body fluids.

4.0 Statistical Considerations

4.1 Sample size justification

Arm A and Arm B

This is a single-institutional, randomized (for Arm A and Arm B), open label clinical trial. The primary objective for Arm A and Arm B is to compare IL17A expression in vaccine-induced lymphoid aggregates between resected PDAs from patients treated with the combination of GVAX with low dose cyclophosphamide (Cy) and anti-PD-1 antibody vs. the treatment of GVAX with low dose Cy alone. Our targeted accrual goal is 34 evaluable patients, all of whom will be randomized at a 1:1 ratio to treatment Arms A and B. Subjects are considered evaluable if they have an R0 or R1 resection of their tumors and their tumors are pathologically proved stage I/II adenocarcinoma of the pancreas. To achieve our accrual goal of 34 patients for evaluation of primary immunology endpoint, we estimate that approximately 42 eligible patients need to be consented and recruited into the study, according to an unevaluable rate in our prior J0810 neoadjuvant vaccine study and considering approximately 5% of PDAC do not carry K-ras mutations.

The primary efficacy endpoint of this study is IL17A expression level measured in the intratumoral vaccine-induced lymphoid aggregates in the surgical resected PDAs. Our prior J0810 neoadjuvant vaccine study shows that patients who received Cy/GVAX had an IL17A expression level of 1.00 ± 0.57 pg/ml (mean ± standard deviation) in their vaccine-induced lymphoid aggregates. An estimate of the effect size for comparing the combined use of GVAX with low dose Cy and anti-PD-1 antibody (Arm B) and the treatment of GVAX with low dose Cy alone (Arm A) is not available. Originally, we powered our study to detect an increase of 0.50 pg/ml in IL17A among patients in arm B when compared to Arm A. The expected difference between the two treatment arms is smaller than 1 standard deviation hence is relatively conservative. With the originally planned total sample size of 25 patients per arm and an evaluable rate of approximately 87.5%, where we assume that anti-PD-1 antibody is uncorrelated with the evaluability of a patient, calculation of statistical power using a unpaired t-test with a common standard deviation of 0.57 and two-sided 5% Type I error rate indicates that the study there will be at least 81% probability

of detecting an increase of 0.50 pg/ml in IL17A expression level in Arm B (Nivolumab+ Cy/GVAX) vs Arm A (Cy/GVAX alone). Power calculation also indicates that the study is expected to have 90% power to detect an increase of 1 standard deviation (0.57 pg/ml) in IL17A expression level.

In order to check the effect size assumption in our original study design, we did a preliminary analysis with TH17 cells in the surgical resection tumor specimens following the neoadjuvant treatment in the first 19 patients (10 in Arm B and 9 in Arm A) and found that TH17 density in Arm B is 2.2 fold higher than that in Arm A. The difference between Arm A and Arm B is larger than what was projected when we determined the sample size originally. Based on this preliminary data, we assume the coefficient of variation of 0.9, 17 patients per arm gives 82% power to detect a 2.2-fold difference between Arm A and B, using a two-sample t-test with two-sided significance level of 0.05. Therefore, we have revised our accrual goal to 17 evaluable in each Arm A and Arm B. We expect to randomize up to 42 patients to achieve these many evaluable patients.

After opening the study, the study team will be in discussion with the sponsor about the possibility of adding an expended cohort for further studying the treatment effect on clinical outcomes. The decision will be made based on safety and feasibility of study treatments, as well as resources and external information, and it will not be determined based on data analysis. A protocol amendment will be submitted to FDA and IRB if the study team decides to expand the study and/or change the primary endpoint. Sample size re-estimation for the expanded cohort will be performed by the study statistician based on the pooled data. If the decision is not to expand the study, the study statistician will be unblinded to patient treatment assignment and the above primary endpoint can be analyzed after all the evaluable subjects have underwent the surgical resection. The analysis of clinical efficacy, safety and other immunologic endpoints will be carried out after all patients completed their eligible vaccinations. The final analysis of overall survival and disease-free survival will be performed after the last enrolled patient being followed for one year.

Arm C

[redacted]

Arms D

[redacted]

[redacted]

[redacted]

Sample size is not powered to compare between Arm D and Arm A/B/C; however, we will compare T cell responses and other immune cell parameters through multiplex immunohistochemistry for explorative purpose. No formal inference supporting analyses requiring any adjustment to statistical significance level for multiplicity will be performed.

4.2. Analysis of the primary efficacy endpoint

Formalin-fixed, paraffin-embedded (FFPE) tissue slides from surgical specimen of PDAs will be used for immunohistochemistry (IHC) staining of IL17A. PD-1, CD137, and granzyme B. IHC analysis and signal intensity per area of lymphoid aggregates will be quantified using Image Analysis Software described previously[25].

Arms A and B: A stratified t-test at two-sided 5% level of significance will be used to compare the IL17A expression in vaccine-induced lymphoid aggregates between resected PDAs from patients treated with the combination of GVAX with low dose cyclophosphamide (Cy) and anti-PD-1 antibody vs. the treatment of GVAX with low dose Cy alone vs. the combination of GVAX, Cy, anti-PD-1 antibody and anti-CD137 agonist antibody. Data will be tested for non-normality and outliers by the Shapiro-Wilk and Grub's tests, respectively. If the normality assumption is violated, a potential transformation will be sought.

Arms B and C: The change of intratumoral CD8+CD137+ cells before and after neoadjuvant therapy will be compared between patients treated with the combination of GVAX with low dose cyclophosphamide (Cy), anti-PD-1 antibody and anti-CD137 agonist antibody vs. the combination of GVAX, Cy, anti-PD-1 antibody, and vs. the treatment of GVAX with low dose Cy alone, using t-test.

Arm D: Pathologic response rate and immune response rate will be estimated as proportions along with 95% confidence interval. The density of granzyme $B^+PD\text{-}1^+CD8^+$ T cells will be compared between pre- and post-treatment specimens using paired-sample t-test.

4.3 Analysis of safety and toxicity measurements

4.3.1 Safety evaluation

One major endpoint of this study is safety as measured by local and systemic toxicity. The analysis set comprises all patients who receive any dose of protocol therapy will be included in the analysis. These toxicities will be characterized according to the National Cancer Institute Common Terminology Criteria for Adverse Events, Version 5.0, and can be accessed and downloaded via the website: http://ctep.cancer.gov/reporting. All information will be recorded on case report forms.

We have in place both an internal real time monitoring plan and an external real-time monitoring plan to ensure that toxicities are captured and evaluated in a timely, appropriate, and non-biased manner. The first two patients in each arm will be enrolled and treated in a staggered fashion. For the first two staggered patients in each arm, the patients will be followed until the time of surgery or for 2 weeks, whichever occurs first following the neoadjuvant immunotherapy, before the next patient will be treated for the neoadjuvant immunotherapy. Patients will be monitored at regular intervals for a broad range of toxicities, including enhanced inflammatory related complications prior to surgery, delayed surgery related healing, dermatologic toxicity at the vaccine site, GM-CSF-related toxicity, Cy-related toxicity, and immune-related adverse events (irAEs). The combination of pancreaticoduodenectomy and chemoradiation is the standard of care and therefore is not part of the protocol. Although the surgery and chemoradiation are not officially part of this

study, serious adverse events will be collected and reviewed to ensure that the vaccine or the study drug combination is not contributing to additional surgery related adverse events or postoperative complications.

AE data will be listed individually and will be tabulated by type, grade, and relatedness both overall and by organ class. In addition to raw counts, the rates of AEs will be calculated based (number of events/length of follow-up).

Surgical complications will be defined based on Clavien-Dindo classification[36]. At the time when 29 patients were randomized and underwent surgery, the protocol was amended to include stopping rules for post-operative complications. The monitoring rule will focus on Grade IIIa surgical complication or above, which is beyond what may be expected for chemotherapy or resection without immunotherapy, and that may be attributable to the immunotherapy drugs (any component of Cy/GVAX/Nivolumab/urelumab/BMS-986253). If the risk of Grade IIIa or higher surgical complications appears to be greater than 40%, the study will temporarily be halted pending feasibility evaluations. The study may resume after discussion between the principal investigator and the IND sponsor if the surgical co-investigators do not deem there is an association of an increased rate of high grade postoperative complication with any of the study treatments. Specifically, we apply a Bayesian monitoring rule that suspends the enrollment if the posterior probability of risk being greater than 40% is 0.5 or higher. The previous study showed 26% Grade IIIa or above post-operative complications in patients receiving neoadjuvant stereotactic body radiation therapy or chemoradiation therapy[37]. Thus, a Beta (2.5, 5.5) prior, representing the prior guess of a post-operative complications (Grade IIIa or above) rate of 31%, will be used. Starting from the 30th randomized patient, surgical complications will be monitored continuously according to the protocol amendment. **Table 1** shows the number of post-operative complications (Grade IIIa or above) that would need to be observed in order to trigger the stopping guidelines throughout the course of the trial, starting from the 30th patients.

Table 1. The number of post-operative complications (Grade IIIa or above) needed to trigger stopping guidelines throughout the course of the study.

Number of Patients	Number of post-operative complications (Grade IIIa or above) needed to trigger re-evaluation
30	13
31-33	14
34-35	15
36-38	16
39-40	17
41-43	18
44-45	19
46-48	20
49-50	21
51-53	22
54-55	23

Number of Patients	Number of post-operative complications (Grade IIIa or above) needed to trigger re-evaluation
56-58	24
59-60	25
61-63	26
64-65	27
66-68	28
69-70	29
71-73	30
74-75	31
76-78	32

The probability of trigger the stopping was assessed for a range of true underlying post-operative complications (Grade IIIa or above) rates using simulations with 5,000 replicates (**Table 2**).

Table 2. The operating characteristics of the stopping rule based on 5000 simulations.

True rate of post-operative complications (Grade IIIa or above)	% time study stops
10%	<0.1%
15%	<0.1%
20%	0.5%
25%	3.8%
30%	14.8%
35%	38.8%
40%	70.5%
45%	90.4%
50%	98.2%

For each arm, we will tabulate the number, type and degree of toxicities for each cycle of immunotherapy. In addition, we will estimate the proportion of individuals who have an unacceptable toxicity from Day 0 until the day prior to the surgery for the first immunotherapy cycle and within the first 28 days of immunotherapy for each of the following cycles with an exact 95% confidence interval. We will use count, proportion with a 95% exact confidence interval, and Poisson rate with a robust 95% confidence interval to summarize toxicity per patient (total number of treated patients as the denominator), and per treatment cycles (total number of vaccines as the denominator). The primary safety endpoint is the proportion of patients experiencing at least one Grade 3/4 non-lymphopenia AE. The AE rates in the two study arms will be compared using Fisher's exact test and Cochran-Mantel-Haenszel test, stratified by age (≤65, >65). It is anticipated that the rate of treatment-related adverse events will be low in both treatment arms. Based on the previous study, the grade 3/4 non-lymphopenia AE rate in Arm A is expected to be close to 0%.

We do not anticipate that addition of Cy/GVAX to nivolumab or urelumab would significantly increase the toxicity. Hence, the grade 3/4 non-lymphopenia AE rate in Arm B is expected to approximate 4%, which was the reported rate of grade 3/4 AE with the nivolumab monotherapy at 3 mg/kg. [redacted] With the planned sample size, the probability of observing at least 1 patient with grade 3/4 AE in Arm B and D is expected to be 64% and 67%, respectively, and the probability of observing at least 2 AEs is only 26% and 29%, respectively. On the other hand, the probability of observing at least 2 patients with grade 3/4 AE in Arm C is 96% and the probability of observing at least 5 patients with grade 3/4 AEs is 49%. The secondary safety endpoint, that is, incidence of delay in surgery and incidence of surgery-related complications, will be summarized using number, percentage, and the corresponding 95% exact confidence intervals, and will be compared between the two treatment arms by using Fisher's exact test and Cochran-Mantel-Haenszel test, stratified by age (≤65, >65). Considering the adenocarcinoma of the pancreas has very poor prognosis, we consider that an overall rate of the combinational therapy-related toxicities of no more than 5% above that observed in the above-described clinical studies of the monotherapy of nivolumab at 480 mg flat dose or those studies of the nivolumab/urelumab or BMS-986253/nivolumab combination will be considered acceptable.

Dermatologic toxicity measurements of the vaccine sites will be performed. Routine skin biopsy will not be performed. If clinically indicated, however, an optional skin biopsy and/or photographs may be obtained if the subject has a rash, or an unusual vaccine reaction.

4.3.2 Additional Feasibility Evaluation

The feasibility of administrating vaccine and nivolumab in the neoadjuvant setting will be assessed from two aspects. From one aspect, we will evaluate unacceptable toxicities as defined in **Section 3.4.7.2** immediately and follow the stopping rules as described in **Section 3.7**. The neoadjuvant treatment will be considered feasible if as described above, any study-related toxicity including those that occur following the neoadjuvant therapy is no more than 5% above that observed in the above-described studies of monotherapy of nivolumab at 480 mg flat dose or those studies of the nivolumab/urelumab or BMS-986253/nivolumab combo.

From another aspect, we will evaluate the surgical data as described below. Because the toxicities related to the study treatment may not be recognized if the same toxicities frequently occur due to the Whipple procedure (see Section 6.5), we will compare the surgical data collected from this study with the historical database at Johns Hopkins once the entire study is completed. We do not expect that the surgery would be delayed by the toxicities related to the study drug treatment. We also do not expect that the study drug administration will influence the resectability of pancreatic tumors. We will assess any unanticipated postponement of surgery and its relations to the administration of study drugs. We will also collect the data on the completeness of the resection (R0, R1 or R2), duration of the operation, blood loss, the length of stay, the need for re-admission within 30 days of surgery, and any intraoperative or postoperative complications. We will compare these data with the historical data of non-vaccinated patients after the study is completed. The neoadjuvant combinatorial immunotherapy will be considered feasible if there is no

statistically significant difference (to suggest adverse clinical outcomes due to the neoadjuvant therapy) between this study and our existing database of non-treated patients, using a one-sample binomial test with the proportion from our existing database as the null proportion. If there is a statistically difference in any of these data, we will determine if any type of toxicities with their grades of severities that are found to be caused by the study drugs either in the previous study or in this study may lead to this difference.

4.4. Analysis of OS and DFS.

The secondary efficacy endpoints are overall survival (OS) and disease free survival (DFS). OS is defined as the time from first dose of study drug to death from any cause. Patients who have not died will be censored at the last date known to be alive. DFS is defined as the time from first dose of study drug until evidence of disease recurrence or death from any cause. For patients who have not progressed, relapsed, or died at the time of analysis, DFS will be censored on the date of last visit where disease progression was evaluable and OS will be censored on the date of last recorded follow-up. For each arm, Kaplan-Meier survival curves will be constructed and the median survival estimates will be calculated with 95% confidence intervals using Greenwood's formula. Survival curves will be compared using log-rank test and multivariable analysis will be considered when appropriate. Because the study sample size is not powered for comparing the OS and DFS and arms are not randomized concurrently, comparisons between treatment arms will be performed for explorative purpose.

4.5 Statistical consideration for immune parameter evaluation

Immune parameters were described in Section 5.2. Continuous variables will be summarized with means or medians and standard deviations. Dichotomous and categorical variables will be summarized using proportions with exact 95% confidence intervals and counts, respectively. Summaries for both pre and post administration of each immunotherapy will be computed. Plots will be used to show the changes in immune response over time both for each individual and for each arm. For each immunotherapy, comparisons in the pre and 28 day post-immunotherapy responses will be compared using paired t-tests (or Wilcoxon signed rank tests if appropriate) for continuous variables and Fisher's exact tests for dichotomous or categorical variables. Associations between immune parameters will be explored graphically (e.g. scatterplots, boxplots) and numerically (e.g. correlations, $\chi 2$ tests). Regression techniques (linear, logistic, linear mixed effects models) will be used to explore the differences between the treatment arms cross-sectionally as well as longitudinally. The associations between immune parameters and clinical outcomes (OS and DFS) will be evaluated using univariate and multivariate Cox regression models. Analyses will be performed using data from all patients and from the subgroup of patients who receive at least three immunotherapies. Sensitivity analyses will be carried out to evaluate the extent to which results of data analyses can be affected by early dropouts. The significance level is set at 0.05 for all tests in this study.

4.6 Analysis of Exploratory Endpoint

Genomic sequencing library construction, whole genome/exome sequencing, whole transcriptome sequencing, microbial sequencing, neoepitope prediction, mutation burden, single-cell profiling, epigenetics, and bioinformatic analysis may be performed either at an on-campus laboratory or at

an off-campus sequencing service. All the samples will be de-identified before sending to any laboratory for sequencing. The BCL, FASTQ files, BAM files and VCF files will be generated and analyzed. Other sequencing assays may be performed on a subset of samples according to specific requirements of collaboration projects.

Genomic sequencing data will be stored and computations conducted using either a JH IT managed subscription of Azure or departmental sever such as the Joint High Performance Computing Exchange (JHPCE).

5.0 Response Criteria

5.1 Evaluation of clinical activity

Most patients will be expected to have only minimal residual disease if they remain to be eligible for this study following the surgery. Therefore, there will be no disease to measure at baseline. Patients will be monitored for disease-free and overall survival. The results from this trial will be compared to the results of our prior neoadjuvant and adjuvant studies, to historical controls seen at the Johns Hopkins Medicine. Patients recently seen at the Johns Hopkins Medicine who can be matched for pathologic stage, surgical intervention, and adjuvant combination chemotherapy and radiation therapy, are the most accurate group of historical controls since our institution has the largest reported experience treating patients with this disease and has recently reported the best survival statistics for current interventions. This study will require that patients undergo standard of care evaluations consisting of abdominal, chest, and pelvis CT scans at Johns Hopkins Medicine at specified intervals to evaluate for local recurrence and distant metastases. In addition, any patient presenting with symptoms will undergo evaluation for metastases. Recurrent disease is defined as evidence of either local or metastatic recurrence by CT scan. The serum tumor marker CA19-9 lacks a sufficient sensitivity and specificity to serve as reliable indicators of response. The CA 19-9 levels will be followed to evaluate whether large and persistent changes might correlate with either in vitro immune responses or with time to clinical recurrence. The methods and frequencies of the follow-up visits utilized by this study are also recommended by the NCCN guideline as standard of care.

5.2 Evaluation of immune parameters

One important goal of the clinical trial is to assess the effects of anti-PD-1 antibody in combination with Cy/GVAX on the PD-L1/PD-1 associated pathways, vaccine-induced immune regulatory signatures, and peripheral and intratumoral antigen specific T cell responses. Specifically, this study will investigate alternate regulatory pathways that may compensate for inhibition of the PD-1/PD-L1 pathway. This study may identify additional signals that need to be targeted for enhancing the efficacy of anti-PD-1 therapy, and/or identify potential resistance mechanisms to anti-PD-1 therapy. Immune regulation signals and immune response parameter within the tumor microenvironment will be tested with tumor specimens obtained from the surgery and those obtained from the biopsy. Systemic immune parameters will be analyzed with regional lymph nodes obtained from the surgery, PBMC, plasma, and serum. All the techniques involved are well-established at Johns Hopkins, and these studies will be performed in close collaboration with appropriate CORE facilities. Assays for evaluation of immune regulation and immune response include, but not limited to the following areas.

Immunohistochemistry IHC of immune parameters relevant to PD-L1/PD-1 associated immune suppressive pathways including PD-L1, PD-1, LAG3, BTLA, TIM3, IDO1, CTLA-4, and Tregs, in immune activation pathway including CD137, OX40, CD40, CD40L, and in cytokines/chemokines or their receptors including CCL2, CCR2, CCL5, CCR5, CCL12, CXCR4 will be performed on FFPE slides of resected PDAs and also on those of pre-treatment core biopsy specimens from both treatment arms. Most of the IHC protocols were previously established (**Supplemental data, Lutz et al.[25]**). The remaining protocols have been established for this current project. We will also examine the densities and distribution of effector T cells including CD8, CD45RO, CXCR3 and $CD69^+$ cells and the expression of T helper cell differentiation markers including Tbet (for Th1), GATA3 (for Th2), RORγT (for Th17), and Foxp3 (for Treg) as previously described. We will evaluate these markers to assess whether anti-PD-1 therapy tips the balance between Teffector, Tregs and different T helper subtypes, and between effector activating and inhibitory immune signals, within post-GVAX lymphoid aggregates. For those markers yielding interesting results, we will select them and perform their IHC on pre-treatment core biopsy specimens,

Polychromatic flow cytometry We will also use polychromatic flow cytometry to quantitatively evaluate the abundance, complexity, phenotype and functional status of tumor infiltrating immune cells. This assay will be performed at the Johns Hopkins Core facilities. The overall focus of the immunomonitoring plan is to quantitate multiple immune cell subtypes ($CD4^+$ and CD8+ T cells, Tregs, myeloid cells, B cells, NK cells) and a variety of well-accepted markers that report the functional status of immune cells. These include the PD-L1/PD-1 relevant immune suppressive markers such as PD-1, CTLA-4, LAG-3, and TIM-3 (markers of T cell inactivation) and immune activation markers such as CD137, OX40, CD40, CD40L, CD69, CD45RO, TNFα, IFNγ and IL-17.

Transcriptional microarray analysis of dissected lymphoid aggregates For explorative purpose, lymphoid aggregates will be dissected as previously described and subjected to microarray analysis of mRNA expression. We will focus on the comparisons between two treatment arms. We will particularly examine the five immune regulatory signatures previously identified in lymphoid aggregates, including the TH17/Treg, NFkB, integrin/adhesion, chemokine/chemokine receptor(e.g. CXCR4, CCL12, CCL2/CCR2, CCL5/CCR5),, and ubiquitin-proteasome pathways (**see Figure S7&S8 in Lutz et al.[25]**), which were found to be associated with favorable responses: longer survival and enhanced peripheral mesothelin specific T cell response (measured by ELISPOT assays as described below) following the neoadjuvant vaccine therapy. The results of this microarray analysis may tell us what signatures are modulated by PD-1 blockade, and identify potential targets for manipulating the TME to promote more potent responses and antitumor activity.

Peripheral Antigen Specific T Cell Response Peripheral mesothelin-specific T cell responses will be measured as an established parameter of immune response to treatment with GVAX. Post-vaccination induction of mesothelin-specific T cell responses in PBL will be measured as previously described and correlated with OS. $CD8^+$ T cells will be enriched by negative selection

using Dynal Dynabeads. HLA-typing will be performed by the Johns Hopkins Immunodiagnostics CORE facility. We will use the same methods we have reported on to assay mesothelin-specific T cell responses in patients with HLA-A1, A2, A3, and A24 alleles. We also previously reported our prediction and validation methods for new peptides. Quantification of mesothelin-specific T cell responses and changes in T cell repertoire will be performed using IFNγ ELISPOT assays. CD8+ T cells specific to each MHC class I restricted mesothelin peptide will be individually quantified in each PBL sample. T2 cells, that naturally express HLA-A2, will be used as antigen-presenting cells (APCs) to present epitopes. We have genetically modified T2 cells to express HLA-A1, A3, and A24. The CEF (CMV, EBV, influenza) pool will be used as positive control peptides; and HIV, renal cell, or melanoma epitopes will be used as negative controls. Similar to our other reported studies, a > 2-fold induction of mesothelin-specific T cell responses will be considered positive. The size of the mesothelin-specific T cell repertoire is considered the total number or percentage of epitopes for which an induction is measured.

Peripheral Tumor Neoepitope-specific T cell Response (including mutated K-ras neoepitope). Through our Next-Generation Sequencing CORE, whole exome sequencing (WES) will be performed on paired patient tumor and normal DNA isolated from FFPE PDA tumor tissue and cryopreserved PBL respectively to identify tumor-specific non-synonymous mutations. RNA sequencing will be performed in parallel to confirm expression of mutant genes identified by WES. Expressed non-synonymous mutations will be screened for candidate neoepitopes using NetMHC HLA-binding predictions and a NetMHC cutoff binding score of 500. Predictions will be made for each tumor-matched HLA class I and class II molecule. We will determine whether the total number of expressed mutations, and/or the total number of predicted neoepitopes for each patient's tumor correlates with improved overall or disease-free survival.

We will use an ELISPOT-based approach similar to the approach we used to define the mesothelin T cell epitopes to validate mutant neoepitopes predicted for HLA-A1 and HLA-A2, including the HLA-A2-binding neoepitopes for the common Kras exon 12 mutations (KrasG12V and G12D). Briefly, wild-type and mutant variant 19 amino acid-long peptides (19mers) spanning each mutation predicted to bind HLA A1 and/or A2, with the mutation site centered, will be pulsed onto T2 APCs and used in ELISPOT assays using autologous pre and post-treatment $CD8^+$ T cells to determine if mutant peptide-specific $CD8^+$ T cell responses are induced by GVAX with or without anti-PD-1 therapy. We will initially use 19mer peptides that can be processed into any possible mutant neoepitope in case the predictions are inaccurate. For each mutant 19mer peptide found to be positive, we will confirm the identity of minimal neoepitopes by repeating the ELISPOT assay using the top predicted 8-11mer candidate epitope(s).

For neoepitopes predicted to have high affinity, HLA multimers will be constructed and used to measure the levels of mutant neoepitope-specific $CD8^+$ T cells in pre and post-vaccination PBL and TIL by FACS; to assess the activation and memory status of mutant neoepitope-specific T cells prior to and following therapy; and to determine whether neoepitope-specific T cells traffic into PDA tumors. TCR repertoire analysis (described below) will also be performed on HLA multimer FACS sorted neoepitope-specific T cells isolated from PBL and TIL to assess the diversity and clonality of the peripheral and intratumoral mutant neoepitope-specific T cell

repertoire, and to identify unique CDR3's expressed by neoepitope-specific T cell receptors. These CDR3's will be used as unique tags for tracking neoepitope-specific T cell clonal frequencies in blood and tissues.

Human PDA tumors typically express 45 non-synonymous mutations. We will initially focus on mutations predicted to generate high affinity neoepitopes (NetMHC scores <500). We will focus especially on mutations that have a positive differential agretopic index (DAI; the difference between the predicted wild-type and mutant affinities for a particular HLA), meaning that the mutant version would be predicted to bind with higher affinity than the wild-type analogs. A recent study suggests that DAI may be more accurate than HLA binding predictions alone for predicting neoepitopes. We will compare both approaches for neoepitope prediction. Most studies focus only on neoepitopes predicted to bind with high affinity. However, we and others have identified T cell epitopes with NetMHC-binding scores up to 10,000 that would not be predicted to bind. Therefore, to avoid excluding this type of epitope, we will also screen 19mer peptides spanning mutations with poor binding predictions for HLA-A1 and A2 (NetMHC scores >500 and <10,000) in our ELISPOT assays, especially when T cell responses to high affinity neoepitopes are not detected, or when no high affinity neoepitopes are predicted for a patient's tumor. The results of these studies will help determine how rare low-affinity neoepitopes are; and also help determine whether a mutation can be ruled out as a candidate based on its predicted HLA-binding or DAI scores.

Intratumoral Antigen Specific T cell Response. Tumor antigen-specific T cells that traffic into the tumor are the most relevant T cells to study when evaluating antitumor immune responses. Although our previous data have shown that mesothelin-specific T cell responses that are induced in PBL are associated with improved survival, GVAX targets multiple antigens. In addition, the typical numbers of TIL that are recovered from resected PDA are not sufficient for mesothelin specific T cell analysis by ELISPOT, after TILs have been prioritized for other analyses. Therefore, rather than focusing specifically on mesothelin-specific T cells in the TME, we will use a more comprehensive approach and examine the TCR repertoire in PBL and TIL, either directly from FFPE tumor sections, or following isolation, using next-generation sequencing. We will compare TCR repertoires in pre-vaccination vs. post-vaccination PBL and in pre-neoadjuvant vaccine specimens (biopsy) vs. pos-neoadjuvant specimens (resected tumors) from the same patients to identify TCR's expressed by T cells that expand following treatment. We will also compare TCR repertoires in PBL vs TIL from the same patients to identify any T cell clones that are induced or expanded in PBL by vaccine and also enriched in TIL. We will use these data to determine if T cells in PBL and TIL undergo clonal expansion following GVAX treatment, and if so, whether the vaccine-expanded T cell clones traffic to the PDA TME. We will also compare the size and diversity of the intratumoral TCR repertoire between the two treatment arms to determine if particular TCR repertoire patterns correlate with the type of treatment. We will also attempt to identify common TCR sequences present in the TME that are shared between patients.

Plasma Analysis. Plasma samples may be sent to outside institutions (including the University of Pennsylvania) or commercial vendors (including Natera and Singlera) for exosome analysis, whole exome sequencing, and methylation analysis of ctDNA that may predict the response to treatment

and help identify additional targets for anti-cancer treatments. A portion of matched archived tumors will be sent out to the same entities to assist ctDNA analysis.

6.0 Adverse Event Reporting

This study will use the descriptions and grading scales found in the revised CTCAE version 5.0 for AE reporting that can be found at http://ctep.cancer.gov/protocolDevelopment/electronic_applications/ctc.htm.

Information about all AEs, whether volunteered by the subject, discovered by investigator questioning, or detected through physical examination, laboratory test or other means, will be collected, recorded, and followed as appropriate.

All AEs experienced by subjects will be collected and reported from the first dose of the investigational agent, throughout the study, and for 28 days after the last dose of investigational agent except for during standard of care treatment periods as explained in the Section 6.6.1. All SAEs will be collected for 100 days after the last nivolumab, urelumab, and/or BMS-986253 treatment for patients in Arm B or 28 days after the last Cy/GVAX for patients in Arm A, or until initiation of a new anti-cancer treatment, whichever occurs first.

Subjects who have an ongoing AE related to the study procedures and/or medication(s) may continue to be periodically contacted by a member of the study staff until the event is resolved or determined to be irreversible by the investigator.

In order to standardize the management of AEs for all subjects, treatment management algorithms are included in **Appendix B.**

Laboratory abnormalities: Laboratory abnormalities present at the screening visit will be recorded as pre-treatment signs and symptoms. After study treatment administration, all grade 3 and 4 clinical laboratory results that represent an increase in severity from baseline will be reported as AEs. A grade 1 or 2 clinical laboratory abnormality should be reported as an AE only if it is considered clinically significant by the investigator, meets the definition of an SAE, required interruption or discontinuation of study drug, or requires specific corrective therapy. Whenever possible, the CTCAE term should be used (anemia vs low hemoglobin, etc.).

6.1 Definitions

6.1.1 Adverse Event

An AE is defined as any undesirable sign, symptom or medical condition occurring after starting the study drug (or therapy) even if the event is not considered to be related to the study. An undesirable medical condition can be symptoms (e.g., nausea, chest pain), signs (e.g., tachycardia, enlarged liver) or the abnormal results of an investigation (e.g., laboratory findings, electrocardiogram). Medical conditions/diseases present before starting the study treatment are only considered AEs if they worsen after starting the study treatment (any procedures specified in the protocol). AEs occurring before starting the

study treatment but after signing the informed consent form will not be recorded. Abnormal laboratory values or test results constitute AEs only if they induce clinical signs or symptoms or require therapy.

6.1.2 Serious Adverse Event

A SAE is an undesirable sign, symptom or medical condition which:

- Results in death
- Is life threatening (defined as an event in which the subject was at risk of death at the time of the event; it does not refer to an event which hypothetically might have caused death if it were more severe)
- Requires inpatient hospitalization or causes prolongation of existing hospitalization (see note below for exceptions) for >24 hours
- Results in persistent or significant disability/incapacity
- Is a congenital anomaly/birth defect (note: reports of congenital anomalies/birth defects must also be reported on the Pregnancy Supplemental Form)
- Is an important medical event (defined as a medical event(s) that may not be immediately life-threatening or result in death or hospitalization but, based upon appropriate medical and scientific judgment, may jeopardize the subject or may require intervention [e.g., medical, surgical] to prevent one of the other serious outcomes listed in the definition above.). Examples of such events include, but are not limited to, intensive treatment in an emergency room or at home for allergic bronchospasm; blood dyscrasias or convulsions that do not result in hospitalization.)
- Potential drug induced liver injury (DILI) is also considered an important medical event.
- Hemophagocytic lymphohistiocytosis
- Suspected transmission of an infectious agent (e.g., pathogenic or nonpathogenic) via the study drug is an SAE.
- Is a new cancer (that is not a condition of the study)
- Is associated with an overdose
- Is a pregnancy

Events **not** considered to be SAEs are hospitalizations for:

- Admissions as per protocol for a planned medical/surgical procedure or to facilitate a procedure
- Elective surgery, planned prior to signing consent
- Routine health assessment requiring admission for baseline/trending of health status (e.g., routine colonoscopy)
- Medical/surgical admission for purpose other than remedying ill health state and was planned prior to entry into the study. Appropriate documentation is required in these cases.
- Admission encountered for another life circumstance that carries no bearing on health status and requires no medical/surgical intervention (e.g., lack of housing, economic inadequacy, care-giver respite, family circumstances, administrative).
- Admissions for monitoring of treatment-related infusion reactions that do not otherwise meet the criteria for a SAE.

- Admission for administration of anticancer therapy in the absence of any other SAEs

6.2 Assessment of Causality

The relationship of an AE to the administration of the study drug is to be assessed by the investigator according to the following definitions:

- No (unrelated, not related, no relation): The time course between the administration of study drug and the occurrence or worsening of the adverse event rules out a causal relationship and another cause (concomitant drugs, therapies, complications, etc.) is suspected.
- Yes (related): The time course between the administration of study drug and the occurrence or worsening of the adverse event is consistent with a causal relationship and no other cause (concomitant drugs, therapies, complications, etc.) can be identified.

The following factors should also be considered:

- The temporal sequence from study drug administration - The event should occur after the study drug is given. The length of time from study drug exposure to event should be evaluated in the clinical context of the event.
- Resolution of the event with immunosuppressive drugs or corticosteroids
- Positive dechallange or positive rechallenge – the event stops when study drug is stopped and/or recurs after resuming study drug
- Underlying, concomitant, intercurrent diseases - Each report should be evaluated in the context of the natural history and course of the disease being treated and any other disease the subject may have.
- Concomitant medication - The other medications the subject is taking or the treatment the subject receives should be examined to determine whether any of them might be recognized to cause the event in question.
- Known response pattern for this class of study drug - Clinical and/or preclinical data may indicate whether a particular response is likely to be a class effect.
- Exposure to physical and/or mental stresses - The exposure to stress might induce adverse changes in the recipient and provide a logical and better explanation for the event.
- The pharmacology and pharmacokinetics of the study drug - The known pharmacologic properties (absorption, distribution, metabolism, and excretion) of the study drug should be considered.

6.3 Assessment of Grade:

The investigator will make an assessment of grade for each AE and SAE reported during the study, which will be recorded in the CRF. The assessment will be based on the National Cancer Institute's CTCAE (Version 5.0) and graded as shown below:

- Grade 1: Mild; asymptomatic or mild symptoms; clinical or diagnostic observations only; intervention not indicated
- Grade 2: Moderate; minimal, local or noninvasive intervention indicated; limiting age-appropriate instrumental activities of daily living
- Grade 3: Severe or medically significant but not immediately life-threatening;

hospitalization or prolongation of hospitalization indicated; disabling; limiting self-care activities of daily living

- Grade 4: Life-threatening consequences; urgent intervention indicated
- Grade 5: Death related to AE

Any AE that changes in grade during its course will be recorded in the CRF at the highest level experience by the subject.

6.4 Expectedness

Unexpected AE: An AE, which varies in nature, intensity or frequency from information on the investigational drug/agent provided in the product IB, package insert or safety reports. Any AE that is not included in the IB consent is considered "unexpected".

Expected (known) AE: An AE, which has been reported in the IB. An AE is considered "expected", only if it is included in the IB document as a risk.

6.5 Handling of Expedited Safety Reports

In accordance with local regulations, the IND Sponsor will notify investigators of all SAEs that are unexpected (i.e., not previously described in the Investigator's Brochure (IB) and/or package inserts), and related to study drug. BMS will notify investigators of all reported SAEs that are unexpected (not previously described in the IB) and related to nivolumab, urelumab, or BMS-986253. This notification will be in the form of an expedited safety report (ESR) or Suspected Unexpected Serious Adverse Reaction (SUSAR) that is to be emailed or faxed to the investigators and the study coordinators. Upon receiving such notices, the investigator must review and retain the notice with the IB and where required by local regulations, the investigator will submit the ESR or SUSAR to the appropriate IRB. The investigator and IRB will determine if the informed consent requires revision. The investigator should also comply with the IRB procedures for reporting any other safety information.

6.6 Reporting

6.6.1 Adverse Events and Serious Adverse Events

All AEs (both expected and unexpected) will be captured on the appropriate study-specific CRFs with the exception of adverse events that occur during standard of care treatments. After Cycle 1 of immunotherapy, participants undergo standard of care surgery. Surgical complications will be collected in a separate log, but will not be recorded as adverse events unless determined to be related to immunotherapy. Adverse Events will also not be collected between Cycle 2 Day 28 (or Day 14 for Arm D after evaluation of Cycle 2 AEs) and the start of Cycle 3, while subjects are receiving standard of care adjuvant chemotherapy and radiation.

Adverse Events that are routinely collected according to GCP shall be submitted to BMS every three (3) months by the last working day of the third month. The Adverse Event information required to be sent to BMS is noted in an attached 'Bristol-Myers Squibb Early Asset Investigator Sponsored Research (ISR) Import Plan' which describes the method of collection and submission to BMS via the mailbox:

[redacted]

When the file is submitted to BMS, it must be noted whether the file contains all Non-Serious Adverse Events (only adverse events not previously submitted to BMS within the 3 months).

All SAEs will be collected for 100 days after the last nivolumab, urelumab, and/or BMS-986253 treatment for patients in Arms B, C, or D or 28 days after the last Cy/GVAX for patients in Arm A, or until initiation of a new anti-cancer treatment, whichever occurs first.

In addition, all SAEs, regardless of causality to study drug and/or administration device, will be reported promptly to the IND sponsor (fax: [redacted], e-mail: [redacted]) and BMS (fax: [redacted], e-mail: [redacted]) within 24 hours of recognition of the AE. If this falls on a weekend or holiday, an email notification is acceptable but must be followed by an SAE reporting form on the next business day.

The Investigator will reconcile the clinical database SAE cases (case level only) transmitted to BMS Global Pharmacovigilance ([redacted]).

- The Investigator will request from BMS GPV&E, [redacted] the SAE reconciliation report and include the BMS protocol number every 3 months and prior to data base lock or final data summary
- GPV&E will send the investigator the report to verify and confirm all SAEs have been transmitted to BMS GPV&E.
- The data elements listed on the GPV&E reconciliation report will be used for case identification purposes. If the Investigator determines a case was not transmitted to BMS GPV&E, the case should be sent immediately to BMS ([redacted]).

In addition to the Sponsor Investigator's responsibility to report events to their local HA, suspected serious adverse reactions (whether expected or unexpected) shall be reported by BMS to the relevant competent health authorities in all concerned countries according to local regulations (either as expedited and/or in aggregate reports).

In accordance with local regulations, BMS will notify sponsor investigators of all reported SAEs that are suspected (related to the investigational product) and unexpected (i.e., not previously described in the IB). An event meeting these criteria is termed a Suspected, Unexpected Serious Adverse Reaction (SUSAR). Sponsor investigator notification of these events will be in the form of either a SUSAR Report or a Semi-Annual SUSAR Report.

6.6.2 Follow-up of Adverse Events and Serious Adverse Events

After the initial AE or SAE report, the investigator is required to proactively follow each subject and provide further information to the safety department in regards to the subject's

condition.

All AE(s) and SAE(s) will be followed until:

- Resolution
- The condition stabilizes
- The event is otherwise explained
- The subject is lost to follow-up

Once the event is resolved, the appropriate AE or SAE report page will be updated. The investigator will also ensure that the follow-up includes any supplemental information that may explain the causality of the event(s). New or updated information will be recorded on the originally completed AE or SAE report, with all changes signed and dated by the investigator or designee. The updated AE or SAE report will then be signed by the investigator and resubmitted to the IND Sponsor.

6.6.3 Overdose

An overdose is defined as the accidental or intentional administration of any dose of a product that is considered both excessive and medically important. All occurrences of overdose must be reported as SAEs.

6.6.4 Potential Drug Induced Liver Injury (DILI)

Wherever possible, timely confirmation of initial liver-related laboratory abnormalities should occur prior to the reporting of a potential DILI event. All occurrences of potential DILIs, meeting the defined criteria, must be reported as SAEs. Potential drug induced liver injury is defined as:

1) ALT or AST elevation > 3 times upper limit of normal (ULN)
   AND
2) Total bilirubin > 2 times ULN, without initial findings of cholestasis (elevated serum alkaline phosphatase)
   AND
3) No other immediately apparent possible causes of AST/ALT elevation and hyperbilirubinemia, including, but not limited to, viral hepatitis, pre-existing chronic or acute liver disease, or the administration of other drug(s) known to be hepatotoxic.

6.6.5 Pregnancy Reporting

Although pregnancy and lactation are not considered AEs, it is the responsibility of investigators or their designees to report any pregnancy or lactation in a subject (spontaneously reported to them) that occurs during the trial or within 23 weeks after the last dose of nivolumab, urelumab, and/or BMS-986253. The investigator must notify both the IND sponsor and BMS of any pregnancy occurring during this time using the same reporting time frame and methods as an SAE. All subjects who become pregnant must be followed to the completion/termination of the pregnancy. Pregnancy outcomes of spontaneous abortion, missed abortion, benign hydatidiform mole, blighted ovum, fetal death, intrauterine death, miscarriage and stillbirth must be reported as SAEs (Important

Medical Events). If the pregnancy continues to term, the outcome (health of infant) must also be reported to the IND sponsor and BMS.

Pregnancy reporting outlined above also includes the pregnancy of a male subject's female partner that occurs during the trial or within 31 weeks days of the last dose of nivolumab, urelumab, or BMS-986253. In order for the investigator to collect any pregnancy surveillance information from the female partner, the female partner must have provided written consent for disclosure of this information.

6.6.6 Institutional Review Board (IRB) and Institutional Biosafety Committee (IBC)

All SAEs will be reported to the IRB and IBC per JHMI institutional guidelines. Follow-up information will be submitted to the IRB and IBC as soon as relevant information is available.

6.6.7 Food and Drug Administration (FDA)

All reporting to the FDA will be completed by the IND Sponsor.

6.6.7.1 Expedited IND Safety Reports:

7 Calendar-Day Telephone or Fax Report:
The IND Sponsor is required to notify the FDA of any fatal or life-threatening adverse event that is unexpected and assessed by the investigator to be possibly related to the investigational agent. Such reports are to be telephoned or faxed (301-827-9796) to the FDA within 7 calendar days of first learning of the event. Follow-up information will be submitted to the FDA as soon as relevant information is available.

15 Calendar-Day Written Report:
The IND Sponsor is required to notify the FDA of any SAE that is unexpected and related to the investigational agent in a written IND Safety Report.

Written IND Safety Reports should include an Analysis of Similar Events in accordance with regulation 21 CFR § 312.32. All safety reports previously filed with the IND concerning similar events should be analyzed. The new report should contain comments on the significance of the new event in light of the previous, similar reports.

Written IND safety reports with Analysis of Similar Events are to be submitted to the FDA within 15 calendar days of first learning of the event. Follow-up information will be submitted to the FDA as soon as relevant information is available.

6.6.7.2 IND Annual Reports

In accordance with the regulation 21 CFR § 312.33, the IND Sponsor shall within 60 days of the anniversary date that the IND went into effect submit a brief report of the adverse events and progress of the investigation. Please refer to Code of Federal

Regulations, 21 CFR § 312.33 for a list of the elements required for the annual report. All IND annual reports will be submitted to the FDA by the IND Sponsor.

6.6.8 Recombinant DNA Advisory Committee (RAC)

Unexpected SAEs believed to be related to the investigational product(s) will be reported to RAC by email if fatal or life-threatening within 7 calendar days or by written report if related and unexpected to the investigational product(s) within 15 calendar days. SAEs that are unrelated or related and expected with the investigational product (s) will be reported to RAC in the Annual Report. Follow-up information will be submitted to the RAC as soon as relevant information is available.

6.7 Special considerations for adverse events that occur during the surgery and postoperative course

Pancreatic surgery is one of the highest-risk procedures, requires prolonged hospitalization, has significant toxicities and is commonly associated with complications and comorbidities. It is deemed to be a standard of care, therefore, is not part of this research study. From the day of surgery through day 0 of the second immunotherapy, participants will be primarily followed by their primary surgeons for monitoring and managing the complications and comorbidities attributable to the surgery. Table 4 summarizes complications commonly associated with the pancreatic surgery based on several published analyses of more than 1000 patients at Johns Hopkins Medicines and other institutions. A normal, uncomplicated postoperative course is still commonly associated with laboratory abnormalities (grade1/2 AEs and occasionally grade 3 AEs) without needs of therapeutic interventions. At least one complication was associated with 58.5% of patients based on a recent analysis of a consecutive series of 633 patients undergoing pancreaticoduodenectomy at Johns Hopkins Medicine between February 2003 and August 2005. Grade I, II, III complications were common, in 10%, 30%, 13.5% of the patients, respectively. These complications and comorbidities are commonly associated with toxicities and laboratory abnormalities of CTCAE grade 3 and even grade 4. By contrast, Grade IV and V complications are relatively uncommon, in 3.0% and 2.0% of the patients, respectively (Table 5).

Table 4 Complications after Pancreaticoduodenectomy

Anastomotic leak, pancreas	Hypotension, shock	Anastomotic leak, intestinal
Wound infection	Cellulitis	Gastrointestinal bleeding
Delayed gastric emptying	Clostridium difficile colitis	Pleural effusion
Hemorrhage, immediate postoperative or delayed	Congestive heart failure, left ventricular dysfunction	Pneumonitis
		Sepsis
Intra-abdominal abscess	Myocardial infarction	Acute respiratory distress syndrome
Fascial dehiscence or evisceration	Renal failure	
	Apnea or hypoxia	Angina, cardiac ischemia
Supraventricular arrhythmia	Atelectasis	Aspiration
Urinary tract infection	Catheter-related infection	Cardiopulmonary arrest
Anastomotic leak, biliary	Deep venous thrombosis	Catheter-related infection
Pancreatitis	Dehydration	Constipation

Delerium tremens
Fever
Fluid imbalance
Gastroesophageal reflux disease
Congestive heart failure
Ileus
Interstitial pneumonitis and fibrosis
Prolonged intubation
Salivary gland infection
Small bowel obstruction

Table 5 Classification of Surgical Complication Adopted for Pancreatic Surgery

Grade	Definition
I	Any deviation from the normal postoperative course without pharmacologic treatment or surgical, endoscopic, and radiological interventions. Allowed therapeutic regimens are: drugs as antiemetics, antipyretics, analgesics, diuretics, electrolytes, and physiotherapy. This grade also includes wound infections opened at the bedside.
II	Requiring pharmacologic treatment with drugs other than ones allowed for grade I complications. Blood transfusion and total parenteral nutrition* are also included.
III	Requiring surgical, endoscopic, or radiologic intervention
IIIa	Intervention not under general anesthesia
IIIb	Intervention under general anesthesia
IV	Life-threatening complication (including CNS complications)† requiring IC/ICU management
IVa	Single-organ dysfunction (including dialysis)
IVb	Multiorgan dysfunction
V	Death of a patient
Suffix "d"	If the patient suffers from a complication at the time of the discharge, the suffix "d" (for disability) is added to the respective grade of complication (including resection of the pancreatic remnant). This label indicates the need for a follow-up to fully evaluate the complication.

*Note regarding DGE: The insertion of a central line for TPN or nasojejunal tube by endoscopy is a grade IIIa. However, if a central line is still in place or a feeding tube has been inserted at the time of surgery, then a TPN or enteral nutrition is a grade II complication.

†Brain hemorrhage, ischemic stroke, subarachnoidal bleeding, but excluding transient ischemic attacks.

CNS indicates central nervous system; IC, intermediate care; ICU, intensive care unit.

Pancreatic surgery is commonly associated with laboratory abnormalities in blood cell counts, serum electrolytes, liver function, and renal function, etc., with a range of severity from grade 1 to grade 4 by CTCAE criteria. Grade I-III complications are common; therefore, laboratory abnormalities associated with a normal postoperative course or Grade I-III complications are considered to be within the commonly expected range of grades of severity (Table 6). Laboratory abnormalities beyond these commonly expected ranges of severity should be considered uncommon. Although Grade IV and V complications have still occurred, any SAE including

laboratory abnormalities associated with Grade IV/V complications should be considered uncommon.

Table 6 Laboratory abnormalities commonly associated with pancreatic surgery

Lab test Abnormality	Ranges of severity by CTCAE criteria	
Amylase	Elevated	Grade 1-4
Lipase	Elevated	Grade 1-4
Bilirubin	Elevated	Grade 1-4
AST	Elevated	Grade 1-4
ALT	Elevated	Grade 1-4
Albumin	Decreased	Grade 1-3
Glucose	Elevated	Grade 1-4
Glucose	Decreased	Grade 1-4
Alk Phosphatase	Elevated	Grade 1-4
Creatinine	Elevated	Grade 1-3
Glomerular filtration rate	Decreased	Grade 1-3
Bicarbonate	Decreased	Grade 1-4
Acidosis	Increased	Grade 1-4
Alkylosis	Increased	Grade 1-4
CPK	Elevated	Grade 1-4
WBC	Elevated	Not graded by CTCAE
Hemoglobin	Decreased	Grade 1-3
Platelets	Elevated	Not graded by CTCAE
Platelets	Decreased	Grade 1-3
Sodium	Elevated	Grade 1-3
Sodium	Decreased	Grade 1-3
Potassium	Elevated	Grade 1-3
Potassium	Decreased	Grade 1-3
Magnesium	Elevated	Grade 1-3
Magnesium	Decreased	Grade 1-3
Phosphate	Elevated	Grade 1-3
Phosphate	Decreased	Grade 1-4

Calcium	Elevated	Grade 1-3
Calcium	Decreased	Grade 1-4

Therefore, during this period, **first**, the study will be focused on monitoring and reporting the complications with uncommon grades of severity such as Grade IV and Grade V complications by criteria used at Johns Hopkins (Table 5). The severity of any SAEs associated with such grades of complications should be considered uncommon. **Second**, any unusual complication not seen commonly with this operation will be reported. **Third,** the study will also be focused on monitoring and reporting any laboratory abnormality beyond the common ranges of severity (Table 6). **Fourth**, the study will also be focused on monitoring and reporting any type of toxicity not commonly attributable to the surgery or postoperative course. These events will be recorded as described in Section 6.1 and their severities will still be categorized by NCI CTCAE v5.0 criteria. Relationship of these events to the investigational drug will be determined by the principal investigator together with surgical co-investigators of the study team and, if necessary, with primary surgeons, and will be categorized as described in Section 6.2. Reporting of these events will follow the same guidelines described in Sections 6.5 and 6.6

7.0 Clinical Trial Monitoring

The protocol will be internally monitored by Dr. Lei Zheng.

The SKCCC Compliance Monitoring Program will provide external monitoring for JHU-affiliated sites in accordance with SKCCC DSMP (Version 6.0, 02/21/2019). The SMC Subcommittee will determine the level of patient safety risk and level/frequency of monitoring.

Additional data and safety monitoring oversight will also be performed by the SKCCC Safety Monitoring Committee (SMC - as defined in the DSMP) and a Medical Expert Committee (MEC) as detailed below. The purpose of these audits or inspections is to systematically and independently examine all trial-related activities and documents to determine whether these activities were conducted and data were recorded, analyzed, and accurately reported according to the protocol, Good Clinical Practice (GCP), and any applicable regulatory requirements. The Medical Expert Committee (MEC) for this clinical study contains three oncologists (see below) from other disciplines who are not affiliated with this clinical trial protocol. The MEC will review safety data on at least a semi-annual basis. The MEC will provide a written summary of each assessment to the IND Sponsor after each meeting. In turn, the study team will forward these summaries to the JHU SKCCC SMC. The operating plan of the MEC will be as follows:

Meetings will be held at least semi-annually, and potentially more frequently if needed. Meetings will be conducted in-person or via video/teleconference, with a participant sign-in sheet collected at each meeting.

Approximately one week prior to each MEC meeting, the study team will submit the following items to MEC personnel for review and discussion at the meeting (The PI may join the MEC meeting in order to answer any questions the MEC might have):

- A summary of the clinical trial's progress to date;

- The latest IRB-approved consent document;
- A summary of all adverse events, serious adverse events, deaths, and withdrawals to date;

Note that the MEC reserves the right to halt trial accrual or all study activity if, after review, serious safety concerns warrant this action. If the MEC halts study accrual or all study activity, then the study team must notify the JHU SKCCC SMC, JHU IRB, JHU IBC, RAC, and the FDA immediately.

8.0 References

1 Ahlgren, J. D. Chemotherapy for pancreatic carcinoma. *Cancer* **78**, 654-663, doi:10.1002/(SICI)1097-0142(19960801)78:3<654::AID-CNCR46>3.0.CO;2-V (1996).

2 Smeenk, H. G. *et al.* Adjuvant 5-FU-based chemoradiotherapy for patients undergoing R-1/R-2 resections for pancreatic cancer. *Digestive surgery* **22**, 321-328, doi:10.1159/000089250 (2005).

3 Huang, A. Y. *et al.* Role of bone marrow-derived cells in presenting MHC class I-restricted tumor antigens. *Science* **264**, 961-965 (1994).

4 Huang AY, B. A., Pardoll DM, Levitsky HI. In vivo cross-priming of MHC class I-restricted antigens requires the TAP transporter. . *Immunity*, 349-355 (1996).

5 Kawakami, Y. *et al.* Cloning of the gene coding for a shared human melanoma antigen recognized by autologous T cells infiltrating into tumor. *Proceedings of the National Academy of Sciences of the United States of America* **91**, 3515-3519 (1994).

6 Cox, A. L. *et al.* Identification of a peptide recognized by five melanoma-specific human cytotoxic T cell lines. *Science* **264**, 716-719 (1994).

7 Dranoff, G. *et al.* Vaccination with irradiated tumor cells engineered to secrete murine granulocyte-macrophage colony-stimulating factor stimulates potent, specific, and long-lasting anti-tumor immunity. *Proceedings of the National Academy of Sciences of the United States of America* **90**, 3539-3543 (1993).

8 Jaffee, E. M. *et al.* A phase I clinical trial of lethally irradiated allogeneic pancreatic tumor cells transfected with the GM-CSF gene for the treatment of pancreatic adenocarcinoma. *Human gene therapy* **9**, 1951-1971, doi:10.1089/hum.1998.9.13-1951 (1998).

9 Jaffee, E. M. *et al.* Novel allogeneic granulocyte-macrophage colony-stimulating factor-secreting tumor vaccine for pancreatic cancer: a phase I trial of safety and immune activation. *Journal of clinical oncology : official journal of the American Society of Clinical Oncology* **19**, 145-156 (2001).

10 Berns, A. J. *et al.* Phase I study of non-replicating autologous tumor cell injections using cells prepared with or without GM-CSF gene transduction in patients with metastatic renal cell carcinoma. *Human gene therapy* **6**, 347-368, doi:10.1089/hum.1995.6.3-347 (1995).

11 Simons, J. W. *et al.* Bioactivity of autologous irradiated renal cell carcinoma vaccines generated by ex vivo granulocyte-macrophage colony-stimulating factor gene transfer. *Cancer research* **57**, 1537-1546 (1997).

12 Soiffer, R. *et al.* Vaccination with irradiated, autologous melanoma cells engineered to secrete granulocyte-macrophage colony-stimulating factor by adenoviral-mediated gene transfer augments antitumor immunity in patients with metastatic melanoma. *Journal of clinical oncology : official journal of the American Society of Clinical Oncology* **21**, 3343-3350, doi:10.1200/JCO.2003.07.005 (2003).

13 Thomas, A. M. *et al.* Mesothelin-specific CD8(+) T cell responses provide evidence of in vivo cross-priming by antigen-presenting cells in vaccinated pancreatic cancer patients. *The Journal of experimental medicine* **200**, 297-306, doi:10.1084/jem.20031435 jem.20031435 [pii] (2004).

14 Lutz, E. *et al.* A lethally irradiated allogeneic granulocyte-macrophage colony stimulating factor-secreting tumor vaccine for pancreatic adenocarcinoma. A Phase II trial of safety, efficacy, and immune activation. *Ann Surg* **253**, 328-335, doi:10.1097/SLA.0b013e3181fd271c (2011).

15 Laheru, D. *et al.* Allogeneic granulocyte macrophage colony-stimulating factor-secreting tumor immunotherapy alone or in sequence with cyclophosphamide for metastatic pancreatic cancer: a pilot study of safety, feasibility, and immune activation. *Clinical cancer research : an official journal of the American Association for Cancer Research* **14**, 1455-1463, doi:14/5/1455 [pii] 10.1158/1078-0432.CCR-07-0371 (2008).

16 Le, D. T. *et al.* Evaluation of ipilimumab in combination with allogeneic pancreatic tumor cells transfected with a GM-CSF gene in previously treated pancreatic cancer. *J Immunother* **36**, 382-389, doi:10.1097/CJI.0b013e31829fb7a2 (2013).

17 Liyanage, U. K. *et al.* Prevalence of regulatory T cells is increased in peripheral blood and tumor microenvironment of patients with pancreas or breast adenocarcinoma. *J Immunol* **169**, 2756-2761 (2002).

18 Woo, E. Y. *et al.* Regulatory CD4(+)CD25(+) T cells in tumors from patients with early-stage non-small cell lung cancer and late-stage ovarian cancer. *Cancer research* **61**, 4766-4772 (2001).

19 Curiel, T. J. *et al.* Specific recruitment of regulatory T cells in ovarian carcinoma fosters immune privilege and predicts reduced survival. *Nature medicine* **10**, 942-949, doi:10.1038/nm1093 (2004).

20 Ercolini, A. M. *et al.* Recruitment of latent pools of high-avidity CD8(+) T cells to the antitumor immune response. *The Journal of experimental medicine* **201**, 1591-1602, doi:jem.20042167 [pii] 10.1084/jem.20042167 (2005).

21 Taieb, J. *et al.* Chemoimmunotherapy of tumors: cyclophosphamide synergizes with exosome based vaccines. *J Immunol* **176**, 2722-2729 (2006).

22 Hermans, I. F., Chong, T. W., Palmowski, M. J., Harris, A. L. & Cerundolo, V. Synergistic effect of metronomic dosing of cyclophosphamide combined with specific antitumor immunotherapy in a murine melanoma model. *Cancer research* **63**, 8408-8413 (2003).

23 Ghiringhelli, F. *et al.* CD4+CD25+ regulatory T cells suppress tumor immunity but are sensitive to cyclophosphamide which allows immunotherapy of established tumors to be curative. *European journal of immunology* **34**, 336-344, doi:10.1002/eji.200324181 (2004).

24 Lutsiak, M. E. *et al.* Inhibition of CD4(+)25+ T regulatory cell function implicated in enhanced immune response by low-dose cyclophosphamide. *Blood* **105**, 2862-2868, doi:2004-06-2410 [pii] 10.1182/blood-2004-06-2410 (2005).

25 Lutz, E. R. *et al.* Immunotherapy converts nonimmunogenic pancreatic tumors into immunogenic foci of immune regulation. *Cancer immunology research* **2**, 616-631, doi:10.1158/2326-6066.CIR-14-0027 (2014).

26 Taube, J. M. *et al.* Association of PD-1, PD-1 ligands, and other features of the tumor immune microenvironment with response to anti-PD-1 therapy. *Clin Cancer Res* (2014).

27 Drake, C. G., Jaffee, E. & Pardoll, D. M. Mechanisms of immune evasion by tumors. *Advances in immunology* **90**, 51-81, doi:10.1016/S0065-2776(06)90002-9 (2006).

28 Topalian, S. L. *et al.* Safety, activity, and immune correlates of anti-PD-1 antibody in cancer. *The New England journal of medicine* **366**, 2443-2454, doi:10.1056/NEJMoa1200690 (2012).

29 Brahmer, J. R. *et al.* Phase I study of single-agent anti-programmed death-1 (MDX-1106) in refractory solid tumors: safety, clinical activity, pharmacodynamics, and immunologic correlates. *Journal of clinical oncology : official journal of the American Society of Clinical Oncology* **28**, 3167-3175, doi:10.1200/JCO.2009.26.7609 (2010).

30 Brahmer, J. R. *et al.* Safety and activity of anti-PD-L1 antibody in patients with advanced cancer. *The New England journal of medicine* **366**, 2455-2465, doi:10.1056/NEJMoa1200694 (2012).

31 Weber, J. S. *et al.* Safety, efficacy, and biomarkers of nivolumab with vaccine in ipilimumab-refractory or -naive melanoma. *Journal of clinical oncology : official journal of the American Society of Clinical Oncology* **31**, 4311-4318, doi:10.1200/JCO.2013.51.4802 (2013).

32 Schalper, K. A. *et al.* Elevated serum interleukin-8 is associated with enhanced intratumor neutrophils and reduced clinical benefit of immune-checkpoint inhibitors. *Nature medicine* **26**, 688-692, doi:10.1038/s41591-020-0856-x (2020).

33 Yuen, K. C. *et al.* High systemic and tumor-associated IL-8 correlates with reduced clinical benefit of PD-L1 blockade. *Nature medicine* **26**, 693-698, doi:10.1038/s41591-020-0860-1 (2020).

34 Orlando, L. *et al.* Prolonged clinical benefit with metronomic chemotherapy in patients with metastatic breast cancer. *Anti-cancer drugs* **17**, 961-967, doi:10.1097/01.cad.0000224454.46824.fc (2006).

35 Bilusic, M. *et al.* Phase I trial of HuMax-IL8 (BMS-986253), an anti-IL-8 monoclonal antibody, in patients with metastatic or unresectable solid tumors. *J Immunother Cancer* **7**, 240, doi:10.1186/s40425-019-0706-x (2019).

36 Clavien, P. A. *et al.* The Clavien-Dindo classification of surgical complications: five-year experience. *Ann Surg* **250**, 187-196, doi:10.1097/SLA.0b013e3181b13ca2 (2009).

37 Blair, A. B. *et al.* Postoperative complications after resection of borderline resectable and locally advanced pancreatic cancer: The impact of neoadjuvant chemotherapy with conventional radiation or stereotactic body radiation therapy. *Surgery* **163**, 1090-1096, doi:10.1016/j.surg.2017.11.027 (2018).

APPENDIX A: Performance Status Criteria

ECOG Performance Status Scale		Karnofsky Performance Scale	
Grade	Descriptions	Percent	Description
0	Normal activity. Fully active, able to carry on all pre-disease performance without restriction.	100	Normal, no complaints, no evidence of disease.
		90	Able to carry on normal activity; minor signs or symptoms of disease.
1	Symptoms, but ambulatory. Restricted in physically strenuous activity, but ambulatory and able to carry out work of a light or sedentary nature (e.g., light housework, office work).	80	Normal activity with effort; some signs or symptoms of disease.
		70	Cares for self, unable to carry on normal activity or to do active work.
2	In bed <50% of the time. Ambulatory and capable of all self-care, but unable to carry out any work activities. Up and about more than 50% of waking hours.	60	Requires occasional assistance, but is able to care for most of his/her needs.
		50	Requires considerable assistance and frequent medical care.
3	In bed >50% of the time. Capable of only limited self-care, confined to bed or chair more than 50% of waking hours.	40	Disabled, requires special care and assistance.
		30	Severely disabled, hospitalization indicated. Death not imminent.
4	100% bedridden. Completely disabled. Cannot carry on any self-care. Totally confined to bed or chair.	20	Very sick, hospitalization indicated. Death not imminent.
		10	Moribund, fatal processes progressing rapidly.
5	Dead.	0	Dead.

APPENDIX B: Management Algorithms for Toxicities associated with Immune-Oncology (I-O) Therapies

These general guidelines constitute guidance to the Investigator and may be supplemented by discussions with the IND Sponsor.

A general principle is that differential diagnoses should be diligently evaluated according to standard medical practice. Non-inflammatory etiologies should be considered and appropriately treated.

Corticosteroids are a primary therapy for immuno-oncology drug-related adverse events. The oral equivalent of the recommended IV doses may be considered for ambulatory subjects with low-grade toxicity. The lower bioavailability of oral corticosteroids should be taken into account when switching to the equivalent dose of oral corticosteroids.

Consultation with a medical or surgical specialist, especially prior to an invasive diagnostic or therapeutic procedure, is recommended.

The frequency and severity of the related adverse events covered by these algorithms will depend on the immuno-oncology agent or regimen being used.

[redacted]

[redacted]

[redacted]

[redacted]

[redacted]

[redacted]

[redacted]

[redacted]
