# Supplementary material for: A platform trial of neoadjuvant and adjuvant antitumor vaccination alone or in combination with PD-1 antagonist and CD137 agonist antibodies in patients with resectable pancreatic adenocarcinoma
Source: Nat Commun. 2023 Jun 20;14:3650. doi: 10.1038/s41467-023-39196-9 (PMC10281953; doi:10.1038/s41467-023-39196-9)
Supplement: Supplementary file 3 — Description of Additional Supplementary Files [file 41467_2023_39196_MOESM3_ESM.pdf]

Description of Additional Supplementary Files

Supplementary Data 1: Disaggregated data at Individual Patient Level
