# Supplementary material for: A platform trial of neoadjuvant and adjuvant antitumor vaccination alone or in combination with PD-1 antagonist and CD137 agonist antibodies in patients with resectable pancreatic adenocarcinoma
Source: Nat Commun. 2023 Jun 20;14:3650. doi: 10.1038/s41467-023-39196-9 (PMC10281953; doi:10.1038/s41467-023-39196-9)
Supplement: Supplementary file 5 — Reporting Summary [file 41467_2023_39196_MOESM5_ESM.pdf]

nature portfolio

Corresponding author(s): Lei Zheng

Last updated by author(s): 5/30/2023

Reporting Summary

Nature Portfolio wishes to improve the reproducibility of the work that we publish. This form provides structure for consistency and transparency in reporting. For further information on Nature Portfolio policies, see our Editorial Policies and the Editorial Policy Checklist.

Statistics

For all statistical analyses, confirm that the following items are present in the figure legend, table legend, main text, or Methods section.

n/a	Confirmed	
☐	☒	The exact sample size (*n*) for each experimental group/condition, given as a discrete number and unit of measurement
☐	☒	A statement on whether measurements were taken from distinct samples or whether the same sample was measured repeatedly
☐	☒	The statistical test(s) used AND whether they are one- or two-sided<br>*Only common tests should be described solely by name; describe more complex techniques in the Methods section.*
☐	☒	A description of all covariates tested
☐	☒	A description of any assumptions or corrections, such as tests of normality and adjustment for multiple comparisons
☐	☒	A full description of the statistical parameters including central tendency (e.g. means) or other basic estimates (e.g. regression coefficient) AND variation (e.g. standard deviation) or associated estimates of uncertainty (e.g. confidence intervals)
☐	☒	For null hypothesis testing, the test statistic (e.g. *F*, *t*, *r*) with confidence intervals, effect sizes, degrees of freedom and *P* value noted<br>*Give P values as exact values whenever suitable.*
☒	☐	For Bayesian analysis, information on the choice of priors and Markov chain Monte Carlo settings
☒	☐	For hierarchical and complex designs, identification of the appropriate level for tests and full reporting of outcomes
☐	☒	Estimates of effect sizes (e.g. Cohen's *d*, Pearson's *r*), indicating how they were calculated

Our web collection on statistics for biologists contains articles on many of the points above.

Software and code

Policy information about availability of computer code

Data collection	Data was collected using Cellprofiler version 2.1.1, Microsoft Excel (2016) and Microsoft Access (2016)
Data analysis	Statistical analyses were performed using R version 4.2.1, GraphPad Prism v9.3.1. No custom code was used. The analysis used standard functions in the software.

For manuscripts utilizing custom algorithms or software that are central to the research but not yet described in published literature, software must be made available to editors and reviewers. We strongly encourage code deposition in a community repository (e.g. GitHub). See the Nature Portfolio guidelines for submitting code & software for further information.

Data

Policy information about availability of data

All manuscripts must include a data availability statement. This statement should provide the following information, where applicable:

- Accession codes, unique identifiers, or web links for publicly available datasets
- A description of any restrictions on data availability
- For clinical datasets or third party data, please ensure that the statement adheres to our policy

Disaggregated data at individual patient level (Supplementary Data 1) and source data for Figures and Tables (Source data file) are provided. All data except the original multiplex IHC staining images are available within the Article, Supplementary Information or Source Data file. The original multiplex IHC staining images are available from the corresponding author upon request. Additional individual de-identified participant data could be shared upon request from the corresponding author. Additional details of the trial, data, contact information, proposal forms, and review and approval process are available at the following website: https://

clinicaltrials.gov/ct2/show/NCT02451982. No custom code is used. The analysis used standard functions in the software.

Human research participants

Policy information about studies involving human research participants and Sex and Gender in Research.

Reporting on sex and gender	We report sex as a descriptive characteristic in our demographic patient statistics. Findings of this study were not significantly impacted by sex or gender.
Population characteristics	Adult patients (18 years old or older), with newly diagnosed (treatment-naïve), resectable, pancreatic adenocarcinoma with adequate performance status and organ function were considered for this study. All patients, regardless of sex/gender/race, who met trial criteria were offered the trial.
Recruitment	Patient were recruited from Johns Hopkins Sidney Kimmel Cancer Center (Baltimore, MD, USA). Patients who would undergo the pancreaticoduodenectomy at the Johns Hopkins Medicine were informed about this trial. Potential participants were identified during chart review in advance of a routine clinic visit or during a routine clinic visit with a provider. Individuals are then approached by the provider or study team to determine willingness to learn more about a study for which they may be eligible. Discussions regarding study participation took place privately and individuals were provided with the IRB approved consent form and other approval materials (e.g., Patient Handout), as applicable. In addition, potential participants may have been in contact the study team directly (in the form of telephone, email, etc., and may be the result of study advertisement, flier, etc., as applicable). Initial discussions regarding study participation in these cases took place by phone, email, etc., and individuals will be provided with the IRB approved consent form and other approval materials (e.g., Patient Handout), as applicable. In all cases, as much time as is needed was allowed for possible participants to consider study participation; resulting in multiple phone calls, visits, emails, or other communication, as necessary. The referring clinicians informed their patients about the research study. The patients indicated to the referring clinician their willingness to contact or be contacted by the study team. The referring clinician(s) appropriately documented their patients' willingness to be referred to the research study in the medical record. If a patient was found to be eligible for this study based on pre-surgical staging and pre-study screening, they will be consented and fully screened for this study. For individuals who chose to take part, informed consent occurred as per the consent process.
Ethics oversight	The study was approved by the Johns Hopkins Institutional Review Board (IRB) and Institutional Biosafety Committee (IBC), as well as the FDA Center for Biologics Evaluation and Research and the National Institutes of Health Recombinant DNA Advisory Committee (J1568, NCT02451982). Informed written consent was obtained from all patients. The trial was conducted according to the Declaration of Helsinki and the Good Clinical Practice guidelines of the International Conference of Harmonization.

Note that full information on the approval of the study protocol must also be provided in the manuscript.

Field-specific reporting

Please select the one below that is the best fit for your research. If you are not sure, read the appropriate sections before making your selection.

[x] Life sciences [ ] Behavioural & social sciences [ ] Ecological, evolutionary & environmental sciences

For a reference copy of the document with all sections, see nature.com/documents/nr-reporting-summary-flat.pdf

Life sciences study design

All studies must disclose on these points even when the disclosure is negative.

Sample size	This study was powered for its biologic endpoints. The sample size of evaluable subjects for the respective treatment groups (n= 17 [Arm A], n=17 [Arm B], n= 10 [Arm C]) provided an 82% power (based on two-sample t-test on log-transformed values) to detect a 2.2-fold difference in IL17A expression levels in tertiary lymphoid aggregates between resected tumor specimens from Arms A and B after neoadjuvant immunotherapy and an 89% power to detect 3-fold difference in intratumoral CD8+CD137+ cells in Arm C resected PDAs compared to Arm B following neoadjuvant study treatment, with two-sided type I error of 0.05 (Study Protocol). The effect size was projected based on correlative studies with Arm A and B (Li et al. Cancer Cell 2022). Since both primary biologic endpoints - 1) comparing IL17A expression between Arm A and B, and 2) comparing CD137+ T cell density between Arm C and B - were each of respective interest, they were not subjected to the multiple comparison adjustment.
Data exclusions	Participants excluded from safety cohort: Out of the original 55 participants consented, 9 did not enroll: 3 did not have pancreatic ductal adenocarcinoma, 1 had liver function tests that were out of eligibility range, 2 had liver metastases on pre-trial baseline imaging, 1 underwent surgical resection prior to neoadjuvant study treatment, and 1 consented participant deferred trial). Since none of these patients initiated study therapy, there were not included in safety nor efficacy analyses.<br>Participants excluded from efficacy cohort: Following standard surgical intervention, 6 additional patients were excluded from efficacy analysis (pre-planned criteria for study continuation): 1 did not have PDAC on surgical pathology, 1 elected not to proceed with surgery, 4 were found to have stage IV disease intraoperatively (peritoneal and/or liver metastasis that was not appreciated on baseline imaging). These patients, since they had received 1st dose of study therapy (prior to planned surgical intervention) were included in the safety analysis but not included in the efficacy (immunologic and clinical) analyses. Excluded Tumor Specimens: Not all tumors specimens from participants included in the efficacy cohort were analysable. Those without an identifiable ROI that contained epithelial neoplastic cells in the vicinity of TLAs were excluded from the correlative analyses. The cases were excluded from the analysis because no tumor cells were identified in the vicinity of

TLAs within the same ROI, likely due to the tumor pathologic response to the neoadjuvant triple immune combo treatment.
Excluded Historical Control Cohort: We excluded patients who had received neoadjuvant chemotherapy (or [chemo]radiation), had T4 and/or M1 disease, and those who had undergone distal pancreatectomy.

Replication	Multiplex IHC analysis was repeated twice with consistent results. There are no reported findings that are not replicated or cannot be reproduced.
Randomization	Eligible subjects were randomized to Arms A and B in a 1:1 ratio, stratified up-front by age (≤65, >65), or enrolled directly to Arm C. Due to the limited availability of Urelumab, enrollment and randomization for Arm A and Arm B was held until the enrollment for Arm C was completed (8/25/2020). Following this, randomized enrollment to Arms A and B was reinitiated until 11/4/2021 when Arms A/B closed early to prioritize enrollment to future platform trial arms.
Blinding	Data acquisition and analysis was blinded

Reporting for specific materials, systems and methods

We require information from authors about some types of materials, experimental systems and methods used in many studies. Here, indicate whether each material, system or method listed is relevant to your study. If you are not sure if a list item applies to your research, read the appropriate section before selecting a response.

Materials & experimental systems

n/a	Involved in the study	
☐	☒	Antibodies
☒	☐	Eukaryotic cell lines
☒	☐	Palaeontology and archaeology
☒	☐	Animals and other organisms
☐	☒	Clinical data
☒	☐	Dual use research of concern

Methods

n/a	Involved in the study	
☒	☐	ChIP-seq
☒	☐	Flow cytometry
☒	☐	MRI-based neuroimaging

Antibodies

Antibodies used	PD-1 inhibitor antibody (nivolumab, BMS-936558), CD137 agonist antibody (urelumab, BMS-663513). For Antibodies used in multiplex immunohistochemistry please refer to supplementary Table 7 (Table S7).
Validation	All antibodies were obtained commercially. These antibodies were tested and validated for their use in human samples by the respective supplier and by us via immunohistochemistry on the basis of the correct staining pattern. All antibodies had validation statement provided on the website of the manufacturer.

Clinical data

Policy information about clinical studies
All manuscripts should comply with the ICMJEguidelines for publication of clinical research and a completedCONSORT checklist must be included with all submissions.

Clinical trial registration	NCT02451982
Study protocol	Included in supplementary materials
Data collection	The clinical trial was conducted at Johns Hopkins Sidney Kimmel Cancer Center (Baltimore, MD). Patients enrolled on study between March 2016 and August 2021. Data collection continued throughout enrollment & trial duration with follow up through May 25, 2022. Data was stored in a HIPAA & IRB compliant database.
Outcomes	The primary endpoint for study Arms A and B was IL17A expression in vaccine-induced lymphoid aggregates in resected pancreatic ductal adenocarcinoma (PDAs) from patients treated with the combination of Cy-GVAX with or without nivolumab . The primary biologic endpoint for Arm C was CD8+CD137+T-cell density within tumor regions of interests (containing at least one TLA) in surgically resected specimens. This study was powered for the primary biologic endpoints. The sample size, and evaluable subjects for respective Arms (n= 17 [Arm A], n=17 [Arm B], n= 10 [Arm C], provided 82% power to detect a 2.2-fold difference in TLA-IL17A expression levels between patient tumors after neoadjuvant study treatment in resected specimens from Arms A & B, and an 89% power to detect 3-fold difference in intratumoral CD8+CD137+ cells in Arm C resected PDAs compared to Arm B following neoadjuvant study treatment, with two-sided type I error of 0.05. Secondary outcomes included the clinical endpoints of disease-free survival (DFS: time from initiation of study treatment to disease recurrence), overall survival (OS: time from initiation of study treatment to death), and safety.
